# An Organoborate Monoxide Radical

**DOI:** 10.1021/jacs.5c20813

**Published:** 2026-01-21

**Authors:** Shuchang Li, Gan Xu, Yong Luo, Zhen Hua Li, Zhenpin Lu

**Affiliations:** † Department of Chemistry, State Key Laboratory of Marine Pollution, City University of Hong Kong, Kowloon Tong, 999077, Hong Kong SAR, P. R. China; ‡ School of Pharmaceutical Sciences (Shenzhen), 26469 Sun Yat-sen University, Shenzhen 518107, P. R. China; § Department of Chemistry, Fudan University, 200433 Shanghai, P. R. China

## Abstract

The boron monoxide radical has emerged as a fascinating molecule and a short-lived intermediate, previously observed only under matrix isolation conditions. In this study, we report the successful synthesis and characterization of a stable organic boron monoxide radical, achieved through the reaction of a diboron (6) dianion with nitric oxide (NO). This oxygen-centered radical is uniquely functionalized and stabilized by a triaryl-substituted boryl group. Comprehensive characterization was performed using various spectroscopic and structural techniques, including electron paramagnetic resonance (EPR) spectroscopy and single-crystal X-ray diffraction analysis. Remarkably, this oxygen-centered radical is stabilized by the K cation and exhibits significant stability under argon at room temperature, showing no self-dimerization even when heated or exposed to UV light. However, it can dimerize to form peroxide species when the K cation is fully encapsulated. Furthermore, it can mimic transition-metal complexes by mediating NO coupling to form a boryl hyponitrite (N_2_O_2_^2–^) derivative. Finally, this boron monoxide radical also shows promising catalytic potential in Sn–Sn coupling reactions.

## Graphical abstract

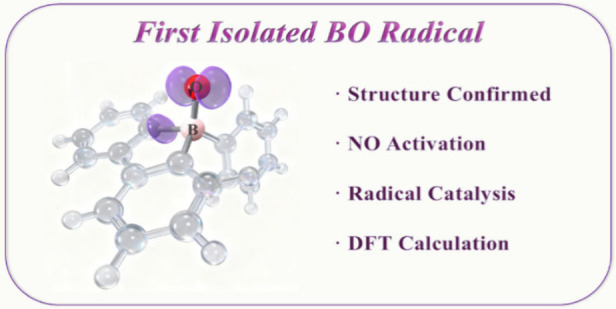

Oxygen-centered radicals play a crucial role in various chemical processes, including combustion, atmospheric chemistry, synthetic chemistry, and biological systems.^1–3^ One of the earliest documented studies on alkoxy radical intermediates dates back to 1911, when Heinrich Wieland proposed the existence of such intermediates during the synthesis of tetraphenyldiphenoxyethane from bis­(triphenylmethyl)­peroxide.^4^ However, their inherent high reactivity often makes them challenging to capture and isolate. In contrast, heteroatom-functionalized oxyl radicals, such as the TEMPO radical (first reported by Lebedev and Kazarnowskii in 1960), exhibit exceptional stability under ambient conditions.^5^ This resilience has established TEMPO as a versatile tool in fundamental research and applied science.^6–8^

In recent decades, organoboron compounds have undergone rapid development across various fields, including small-molecule activation, catalysis, and materials science.^9–23^ Due to its unique electron-deficient character, boron is an excellent choice for creating open-shell species. As a result, numerous boron-containing radical compounds have been reported, demonstrating valuable applications in organic synthesis, polymer chemistry, and materials science.^24–35^ Among these, boron-oxo radicals have been proposed as key intermediates in the well-established Et_3_B/O_2_ radical initiation system, which has been extensively discussed in prior radical chemistry studies.^36,37^ Despite these advancements, isolated boron-oxo radicals have yet to be reported. We envisioned that organoborate monoxide radicals would offer attractive synthetic utility and reveal new reactivity, compared to their carbon and nitrogen analogues.

Early attempts to study boron monoxide radicals mainly focused on the investigation of their structures by using electronic,^38,39^ microwave,^40^ and ESR spectroscopy.^41,42^ Several research groups have attempted to synthesize boron monoxide radicals. To date, however, these species have only been observed under matrix isolation conditions or hypothesized as short-lived intermediates.^43,44^ In this study, we report the first successful synthesis and isolation of a stable organoborate monoxide radical species (Figure 1). This radical represents a novel class of oxygen-centered radicals, and we have further investigated its reactivity to establish its chemical behaviors.

**Figure 1:**
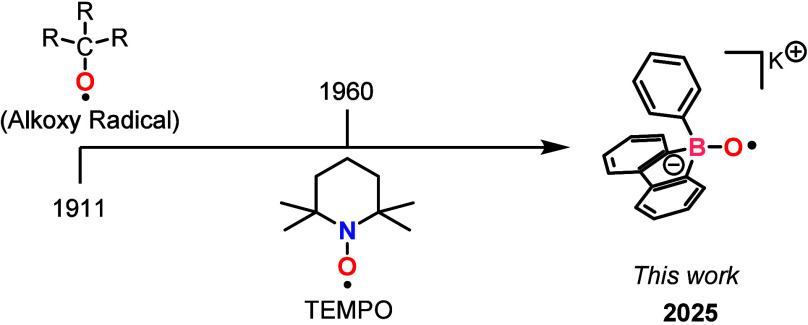
Timeline of the discovery for organic oxygen-centered radicals.

Previously, we demonstrated that the diboron(6) dianion **1** can undergo homolytic B–B cleavage to generate the corresponding boron radical anion.^45^ To further explore its reactivity, the reaction of **1** with NO in a 1:2 ratio was conducted in THF at room temperature, leading to the formation of a new species, **2**. Additionally, N_2_O was traced as the byproduct (see Figure S38). The ^11^B NMR spectrum of **2** displays a singlet at 6.8 ppm. Compound **2** was recrystallized in the presence of crown ether (18-crown-6) from a concentrated THF solution, yielding 47% (Figure 2a). X-ray single-crystal analysis of **2** reveals the formation of a B–O bond at the borafluorene-functionalized boron center, along with the presence of a potassium cation. The B–O bond length (1.468 Å) falls within the typical range for B–O single bonds in alkyl/aryl-substituted boryl ethers (1.35–1.50 Å).^46–49^ However, it is challenging to determine whether the oxygen is solely monosubstituted, as X-ray single-crystal analysis cannot accurately locate the position of hydrogen in the structure. Consequently, the hydroxy-substituted boron anion **3** was also synthesized (Figure 2c). Compound **3** exhibits a signal at 1.56 ppm in the ^11^B NMR, which differs from that of **2** (6.8 ppm). Furthermore, while X-ray single-crystal analysis indicates that compound **3** adopts a framework similar to that of **2**, the B–O bond length in **3** is 1.498 Å (vs 1.468 Å in **2**). It is evident that the B, O, and K atoms are displaced from a plane due to the presence of an OH bond, whereas in **2**, these atoms are coplanar (Figure 2b and 2d). The differences in B–O bond length, alongside the NMR and structural planarity distinctions, provide clear evidence that **2** and **3** are chemically distinct species.

**Figure 2:**
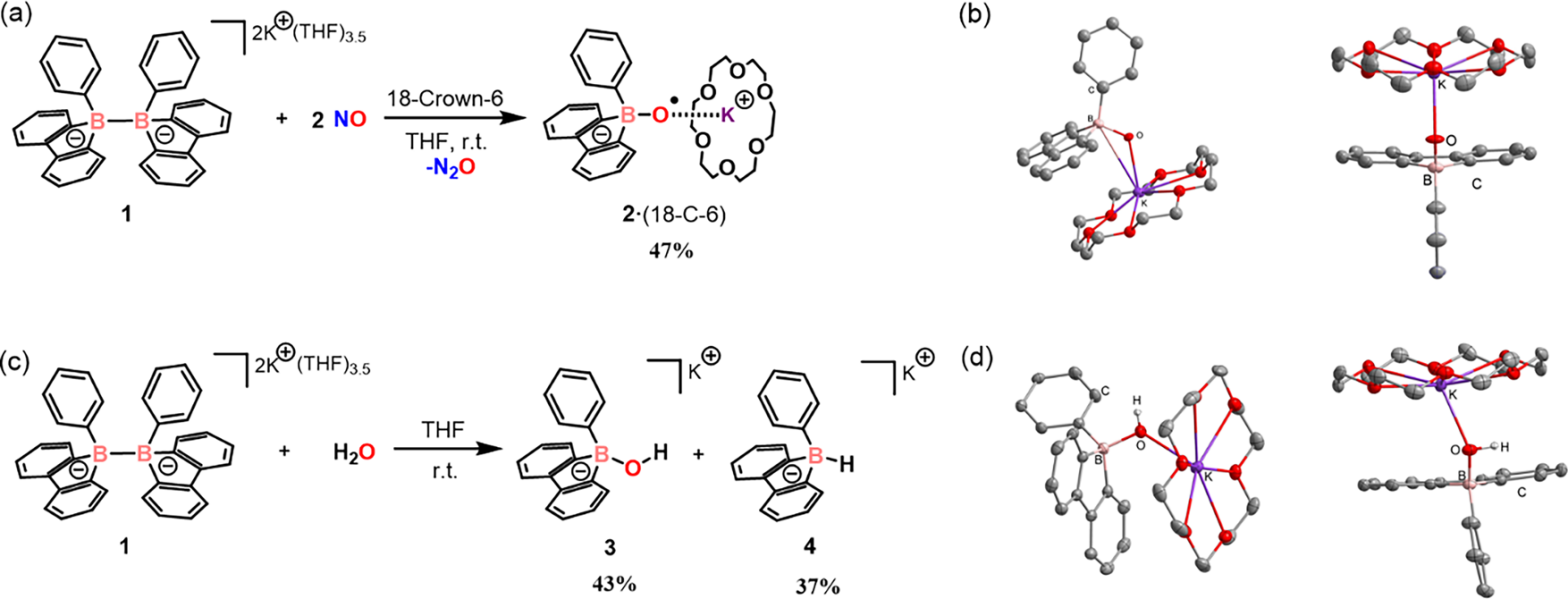
(a) Synthesis of compound **2**·(18-C-6). (b) X-ray single-crystal structure of **2**·(18-C-6) from two different views. (c) Synthesis of compound **3** and **4**. (d) X-ray single-crystal structure of **3**·(18-C-6) from two different views.

The radical character of compound **2** was further characterized using EPR spectra. A signal centered at *g* = 2.000 was observed in the X-band EPR spectra at room temperature (see Figure S40). A simulated spectrum matches well with the experimental data, suggesting the presence of an oxygen-centered radical species. In contrast, compound **3** does not show any signals in the EPR spectra. Additionally, the O–H stretching vibration in compound **3** (3642 cm^–1^) is observed in the IR spectra (see Figure S42), while this signal is absent in compound **2**, indicating that the O–H bond does not exist in **2**.

The geometry of **2** in the THF solution was optimized with the M06-2X/6-31+G­(d,p) method. The results show that it has a BO bond length of 1.437 Å, corresponding to a single B–O bond. Mulliken charge analysis reveals that the spin is primarily localized on the oxygen atom, with a net spin of −0.85 e (Figure 3a). The negative charge, on the other hand, is delocalized on the three aromatic rings, with only −0.33 e on the oxygen atom.

**Figure 3:**
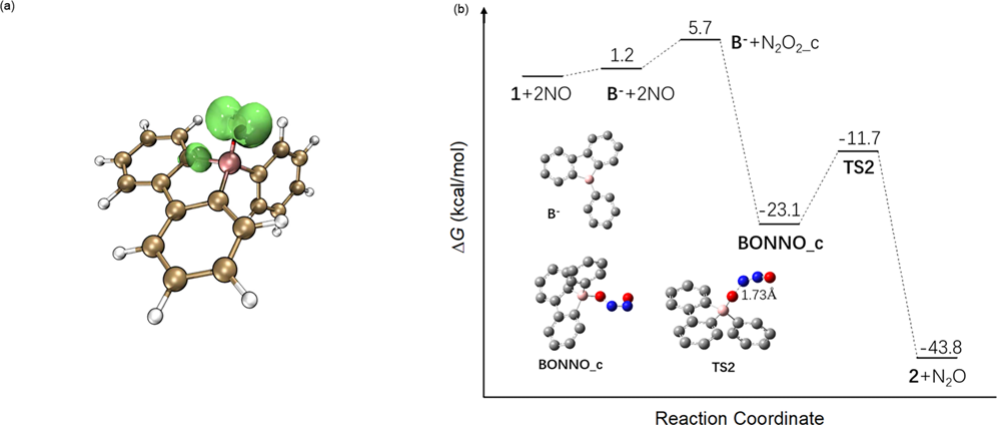
(a) Spin density of the BO radical **2**, isovalue 0.08. (b) Gibbs free-energy profile at 298.15 K for the reaction between **1** and NO to produce **2**.

To elucidate the reaction mechanism, we conducted a computational study on the reaction between **1** and NO to produce **2**, employing the M06-2X/6-311++G­(d,p)//M06-2X/6-31+G­(d,p) method (Figure 3b). NO exists in rapid equilibrium with N_2_O_2_, which has two isomers: one with a trans conformation (**N**_**2**_ **O**_**2**_ **_t**) and the other with a cis conformation (**N**_**2**_ **O**_**2**_ **_c**), with the latter being more stable. Compound **1** and the boron radical anion (**B** ^**–**^ **)** are also in rapid equilibrium. **B** ^**–**^ then reacts barrierlessly with **N**_**2**_ **O**_**2**_ **_c** to produce **BONNO_c**. Subsequently, **BONNO_c** breaks its O–N bond to produce **2** and N_2_O. The highest Gibbs free-energy barrier along the entire reaction pathway is only 11.4 kcal/mol, consistent with the experimental observation that the reaction can proceed readily at room temperature.

## Chemical Stability

Carbon-functionalized oxyl radicals, such as tBuO·, tend to dimerize, forming peroxide derivatives. In contrast, nitrogen-based analogues like TEMPO remain stable as monomers and do not form peroxides. We then sought to investigate the chemical stability of compound **2·(18-C-6)**. When heated in THF at 70 °C or exposed to UV irradiation, compound **2·(18-C-6)** remained unchanged, and no corresponding peroxide species (**5·(18-C-6)**_**2**_) was generated (Figure 4a). Besides, compound **5** can instead be directly synthesized through the reaction of compound **1** with dioxygen (Figure 4b). The formation of the O–O bonded framework has been confirmed through X-ray single-crystal analysis (see Figure S43). Intriguingly, both compounds **2·(18-C-6)** and **5·(18-C-6)**_**2**_ exhibit thermodynamic stability, which is a striking contrast to their carbon and nitrogen analogues, where typically only one species—such as di-*tert*-butyl peroxide or the TEMPO radical (either the oxyl radical or the peroxide)—is thermodynamically stable.

**Figure 4:**
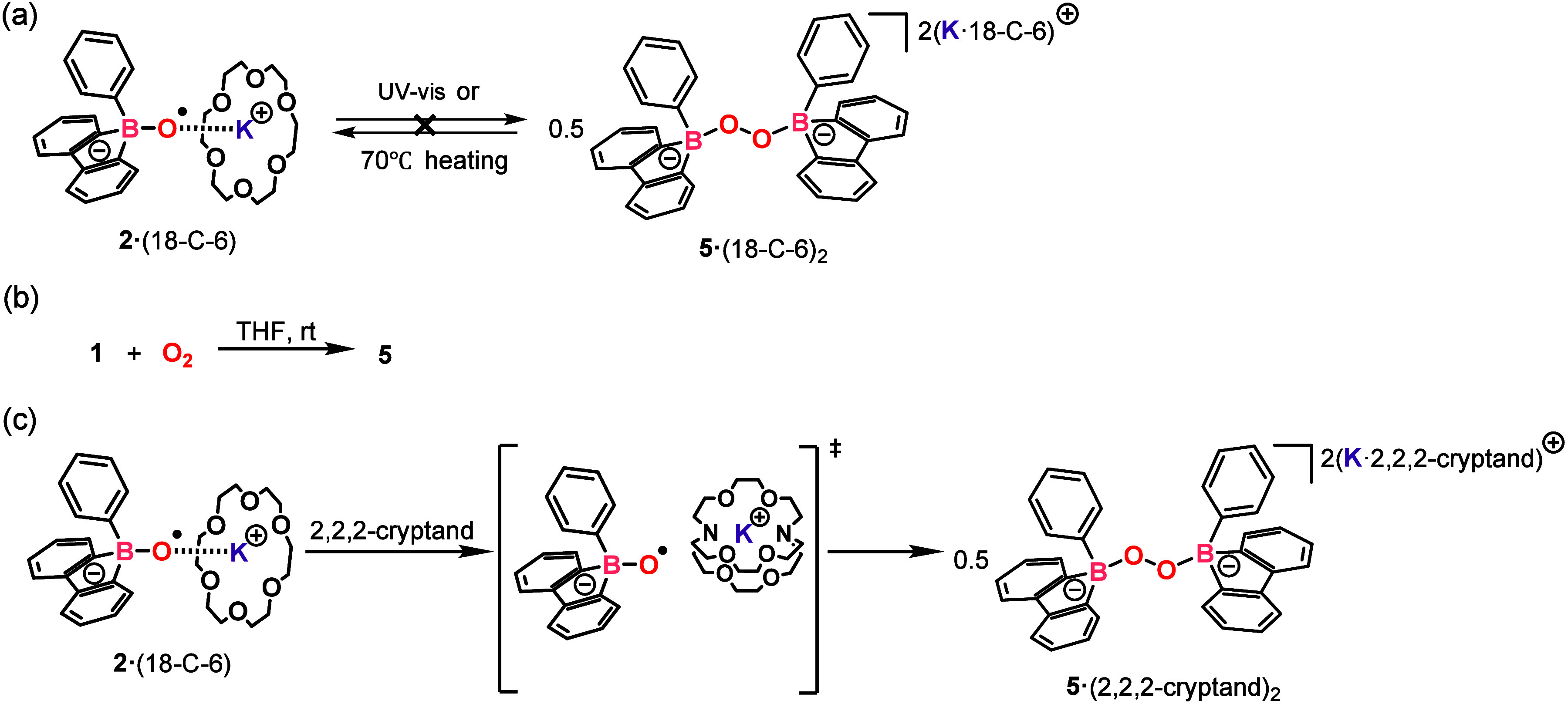
(a) Chemical stability of **2**·(18-C-6). (b) Synthesis of **5** through the reaction between **1** and O_2_. (c) Transformation of **2**·(18-C-6) to **5**·(2,2,2-cryptand)_2_.

To understand the relationship between compound **2** and its dimer, compound **5**, we computed the Gibbs free-energy profile at 298.15 K (Figure 5) for the conversion between **2** and **5** with the M06-2X/6-311++G­(d,p)//M06-2X/6-31+G­(d,p) method. The results indicate that the interconversion between the two species has to overcome a high free-energy barrier, 34.6 kcal/mol from **2** to **5** and 30.6 kcal/mol from **5** to **2**. The high free-energy barrier prevents interconversion between **2** and **5**, and the use of 18-C-6 crown ether was insufficient to overcome this ionization effect. Therefore, 2 equiv of 2,2,2-cryptand were added to the solution of compound **2·(18-C-6)**, leading to the formation of **5·(2,2,2-cryptand)**_**2**_ (Figure 4c). This demonstrates that 2,2,2-cryptand effectively sequesters K^+^, removing the kinetic barrier to the dimerization process.

**Figure 5:**
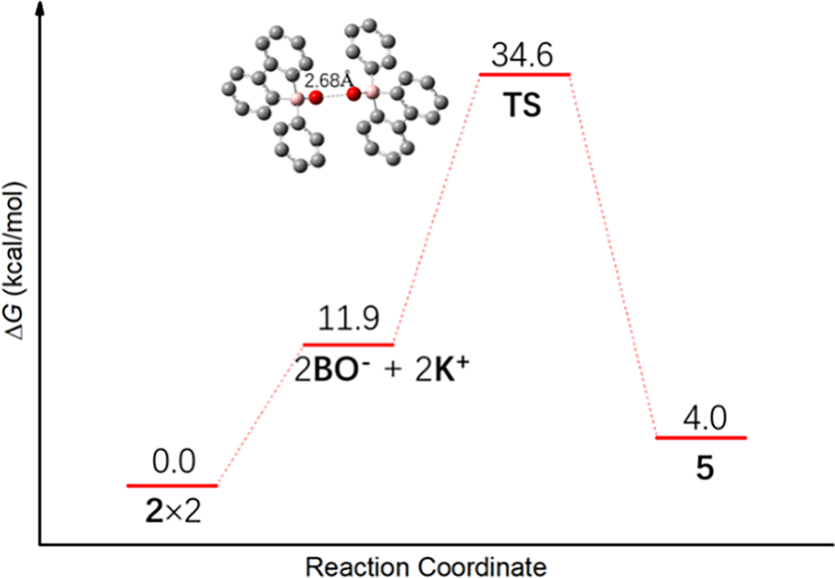
Gibbs free-energy profile at 298.15 K for the conversion between **2** and **5**.

## NO Activation

Nitric oxide (NO) is essential for both atmospheric and physiological processes, with its dimerization to form dinitrogen dioxide (N_2_O_2_) playing a key role in regulating redox reactions. This dimerization significantly influences biological signaling pathways and various environmental processes.^50–52^ Additionally, hyponitrite intermediates can arise from the reductive coupling of two NO molecules via N–N bond formation, which is crucial for biological denitrification. However, these species are often stabilized by transition-metal centers through different binding modes.^53–60^ Previously, Erker and colleagues reported a frustrated Lewis pair-stabilized product featuring an N=N bond resulting from NO coupling.^61^ Nevertheless, the synthesis of hyponitrite derivatives stabilized by main-group elements from NO coupling is extremely rare.

Thus, the reaction of B–O radical **2** and NO was conducted, yielding compound **6a** as the major product (Figure 6). The ^11^B NMR spectrum of compound **6a** exhibits a signal at 4.03 ppm. Its structure has been unambiguously confirmed by X-ray single-crystal analysis (Figure 6), demonstrating the outcome of the NO dimerization product and the formation of a N=N bond. The two BO groups were attached to the N=N bond in a *cis* configuration. The crystals of the trans-isomer, **6b**, can also be obtained and characterized through X-ray single-crystal analysis. However, due to the limited quantity of **6b**, its NMR characterization was hindered. Additionally, we isolated and characterized another minor product, compound **7**. X-ray single-crystal analysis of **7** reveals the formation of a 9-aza-10-boraphenanthrene derivative, similar to that reported by Dewar and colleagues.^62^ The two B,N-fused π systems in **7** are bridged by two oxygen atoms, forming a central six-membered ring containing boron, oxygen, and nitrogen (B, O, N) within its core. Notably, the formation of compound **6a** can also occur through the reaction of compound **1** with an excess of NO. The formation of compounds **6a** and **6b** is proposed to occur via an NO dimerization pathway, supported by the computational study (see Figure S44).

**Figure 6:**
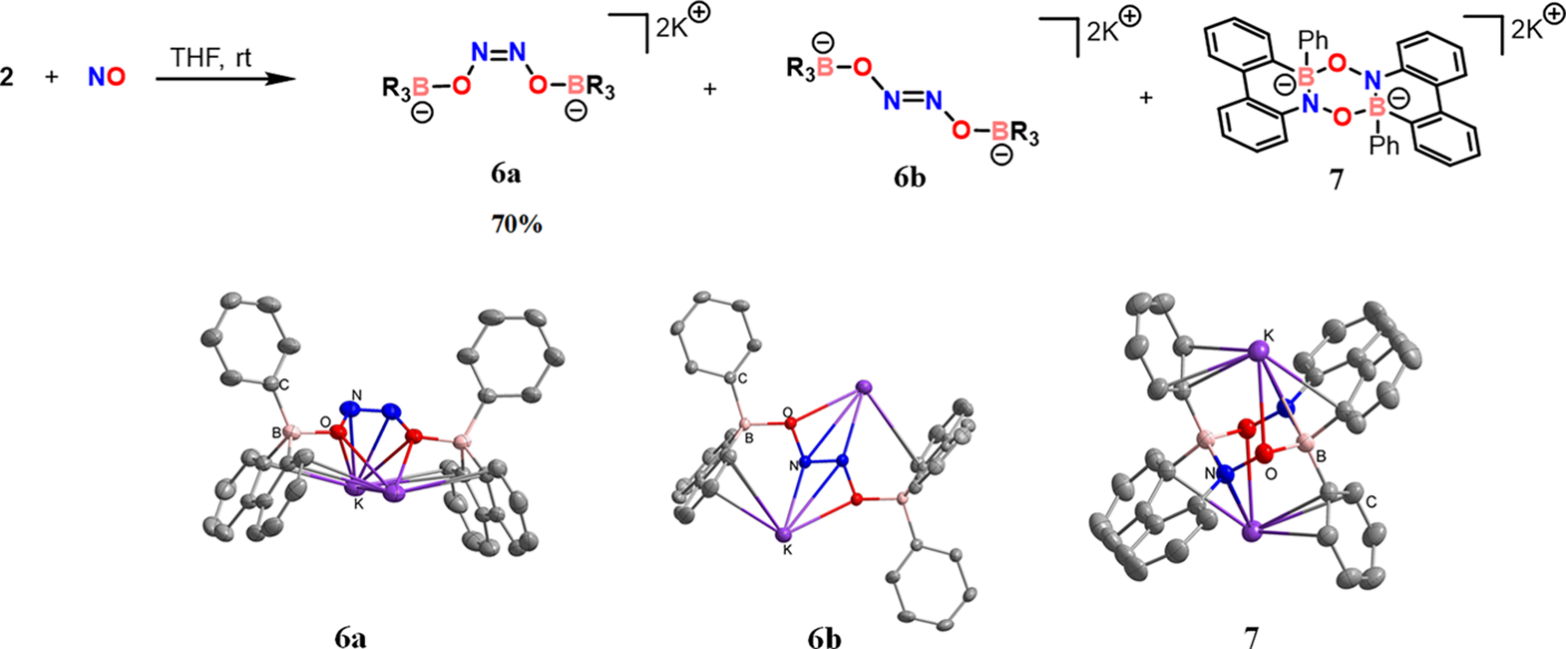
(Top) Reaction of compound **2** and NO and molecular structures of compounds **6a**, **6b**, and **7**. (Bottom) Thermal ellipsoids are set at the 30% probability level; hydrogen atoms and solvents are omitted for clarity.

## Catalysis

Oxygen-centered radicals have been extensively involved in hydrogen atom transfer (HAT) reactions, emerging as some of the most versatile intermediates due to their remarkable O–H bond dissociation energy (BDE).^63^ Consequently, we conducted the reaction of compound **2** with ^*n*^Bu_3_SnH, yielding an Sn–Sn coupled product along with dihydrogen (Figure 7a). As a result, this reaction can also be performed with just 1 mol % of compound **2** as a catalyst, resulting in the Sn–Sn coupled product with an isolated yield of 99% (Figure 7b). Furthermore, compound **3** can also promote such transformation (Figure 7c), suggesting that both compounds **2** and **3** are crucial species within the catalytic cycle (see Scheme S1). Previously, the dehydrogenative coupling of *n*Bu_3_SnH required transition-metal-based catalysts (e.g., Ru, Pt).^64–66^ Notably, the catalytic efficiency of compound **2** not only matches but even surpasses that of these metal-based systems.

**Figure 7:**
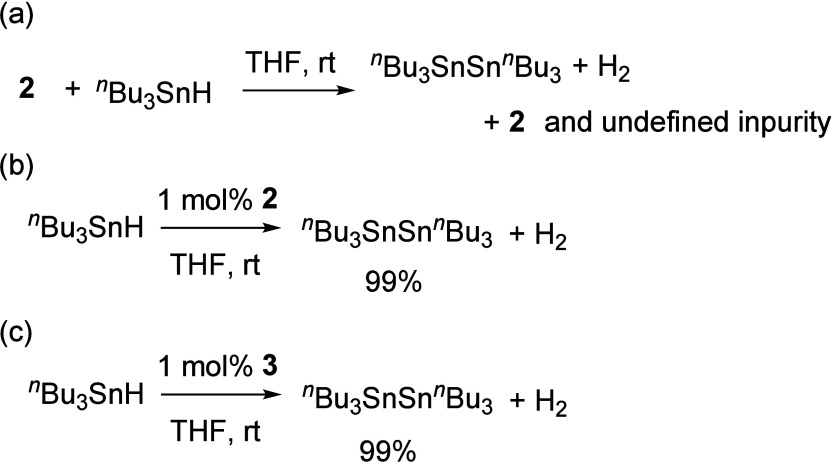
Reactions of compound **2** and *n*Bu_3_SnH and its application in catalytic Sn–Sn coupling.

Over the past century, oxygen-centered radicals have exhibited a diverse range of chemistry and applications across various fields. With the detailed presentation of the structure, synthesis, and reactivity of this boron-oxo radical, we foresee an exciting future for this class of oxyl radical, ripe for further exploration. The unique properties of these radicals may open new avenues in materials science, catalysis, and organic synthesis, potentially leading to innovative applications that could transform existing technologies.

## Supplementary Material



## Notes

Supplementary figures, detailed experimental procedures, and characterization data for all new compounds (PDF)
