# Supplementary material for: An Organoborate Monoxide Radical
Source: J Am Chem Soc. 2026 Jan 21;148(4):3927–32. doi: 10.1021/jacs.5c20813 (PMC12879740; doi:10.1021/jacs.5c20813)
Supplement: Supplementary file 1 [file ja5c20813_si_001.pdf]

Supporting Information for

An Organoborate Monoxide Radical

Shuchang Li,[1] Gan Xu,[1] Yong Luo,[2] Zhen Hua Li,*[3] Zhenpin Lu*[1]

[1] Department of Chemistry, State Key Laboratory of Marine Pollution, City University of Hong Kong, Kowloon Tong, 999077, Hong Kong SAR, P. R. China

[2] School of Pharmaceutical Sciences (Shenzhen), Sun Yat-sen University, Shenzhen, 518107, P. R. China

[3] Department of Chemistry, Fudan University, 200433, Shanghai, China

*Email: lizhenhua@fudan.edu.cn, zhenpilu@cityu.edu.hk

Contents

Synthetic details and characterization data **3**

General Procedures 3

Synthesis of Compound **2**: 4

Synthesis of Compounds **3** and **4**: 5

Synthesis of Compound **5**: 6

Thermodynamic stabilities of Compounds **2·(18-C-6)** and **5·(18-C-6)$_2$**: 7

Transformation of Compound **2·(18-C-6)** to **5·(2,2,2-cryptand)$_2$**: 7

Synthesis of Compound **6a**, **6b**, and **7**: 8

Reaction between **2** and nBu$_3$SnH: 9

Controlled experiment between **3** and nBu$_3$SnH: 9

Proposed mechanism for Sn-Sn Coupling： 10

NMR Spectra: 11

GC-MS, EPR and IR Study: **30**

GC-MS Study 30

EPR Study 31

IR Study 32

X-Ray Single-Crystal Diffraction **33**

Computation details **41**

Reference **67**

Synthetic details and characterization data

General Procedures

All air- and moisture-sensitive reactions were conducted under an inert argon atmosphere using standard Schlenk techniques or a Vigor glovebox equipped with a -35 °C freezer. All reaction tubes and glassware were oven-dried overnight at 180 °C. Chemicals were purchased from Sigma-Aldrich, TCI, Aladdin, and Macklin, and used without further purification. Nitric oxide (NO, Air Liquide 99.99%) was dried over phosphorus pentoxide (P_2O_5) for a total duration of 2 weeks prior to use. SPS-purified tetrahydrofuran (THF), toluene, and n-hexane were dried over 3 Å molecular sieves inside the glovebox. Deuterated solvents (C_6D_6) were degassed via freeze-pump-thaw cycles and stored over 3 Å molecular sieves in the glovebox; THF-d_8 was dried with sodium/potassium (Na/K) alloy prior to use, while deuterated solvents for air-stable products were used without additional treatment.

NMR spectra were obtained on Bruker Avance Neo 400 (NMR-400) and Bruker Avance III HD 600 (NMR-600) spectrometers. NMR samples were prepared under an argon atmosphere and analyzed in J. Young PTFE-valve NMR tubes. Chemical shifts (δ) are presented in ppm, referenced to the residual proton (^{1}H) or carbon (^{13}C{^{1}H}) signals of the respective solvents[1]. B NMR spectra were referenced to an external standard of $BF_3 \cdot OEt_2$ (0 ppm, neat). ^{119}Sn NMR spectra were referenced to an external standard of tetramethyltin (Me_4Sn, 0 ppm), in accordance with IUPAC-recommended referencing protocols for organotin nuclei. Peak assignments for ^{1}H and ^{13}C NMR spectra were confirmed using ^{1}H-^{1}H correlation spectroscopy (COSY), ^{1}H-^{13}C heteronuclear single quantum coherence (HSQC), and ^{1}H-^{13}C heteronuclear multiple bond coherence (HMBC) experiments.

High-resolution mass spectra (HRMS) were acquired on a Sciex X500R Q-TOF mass spectrometer. Elemental analysis was performed by the Analytical Laboratory of the Shanghai Institute of Organic Chemistry, Chinese Academy of Sciences (CAS). Electron paramagnetic resonance (EPR) spectra were recorded using a Bruker EMXPlus-10/12 spectrometer. Solid-state infrared (IR) spectra of Compounds **2** and **3** were collected under an argon atmosphere using a Bruker Alpha Platinum-ATR spectrometer with OPUS software.

Synthesis of Compound 2:

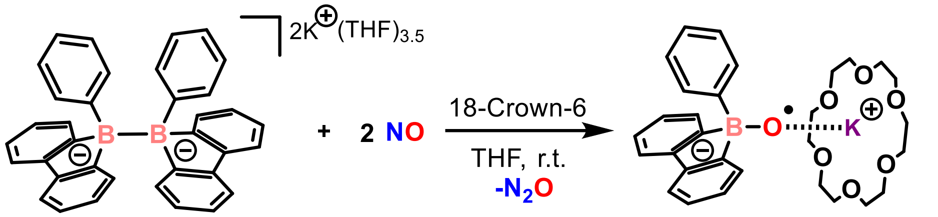

1 **2**·(18-C-6)

47%

The hexaaryl-substituted diboron (6) dianion 1, the starting material, was prepared according to our reported method.[2] A solution of **1** (72.5 mg, 0.086 mmol, 1.0 eq.) in 5 mL of THF was degassed via three consecutive freeze-pump-thaw cycles in a 10 mL Schlenk tube. Dried nitric oxide (NO) (4.2 mL , 0.17 mmol, 2.0 eq.) gas was introduced into the tube using a micro-syringe and a three-way glass stopcock (tee joint) under room temperature. Instantly, the solution's color changed from orange-red to light yellow. The solution was then filtered through Celite on a glass fiber. Hexane was added to wash away the by-products and salts. All volatile components were removed under high vacuum, yielding a white powder as the final product, **2**. Colorless single crystals suitable for X-ray diffraction were obtained from a concentrated THF solution of **2** (with 1.0 eq. of 18-Crown-6) at -35℃. Yield: **2·(18-C-6)**: 22.5 mg, 47%.

Compound 2·(18-Crown-6):

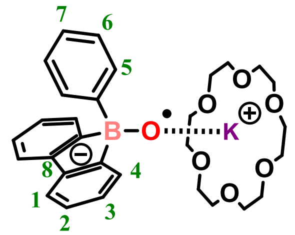

2·(18-C-6)

^{1}H NMR (600 MHz, THF-d_8) δ (ppm) = 7.44 (d, J = 7.5 Hz, 2H, *H*-1), 7.33 - 7.27 (m, 4H, *H*-5, 4), 6.94 (m, 2H, *H*-2), 6.89 - 6.82 (m, 4H, *H*-3, 6), 6.77 (m, 1H, *H*-7), 3.37 (s, 24H, *H* of 18-Crown-6).

^{13}C{^{1}H} NMR (151 MHz, THF-d_8) δ (ppm) = 150.31 (*C*-8), 132.62 (*C*-5), 131.90 (*C*-4), 126.58 (*C*-6), 126.09 (*C*-3), 125.54 (*C*-2), 123.63 (*C*-7), 118.67 (*C*-1), 70.95 (*C*-18-Crown-6).

^{11}B NMR (193 MHz, THF-d_8) δ (ppm) = 6.77.

μ_{eff} = 1.44 μ_B (Evans Method, THF-d_8, 298.5 K)

HRMS($[C_{18}H_{14}BO]^-$): Simulated: 257.1138, Test: 257.1172 (easy to transform to BOH in HRMS system).

Anal. Calcd for **2**·(18-Crown-6)$_{1.2}$: C:63.55; H: 6.88; Found: C:63.18; H:6.86.

Synthesis of Compounds 3 and 4:

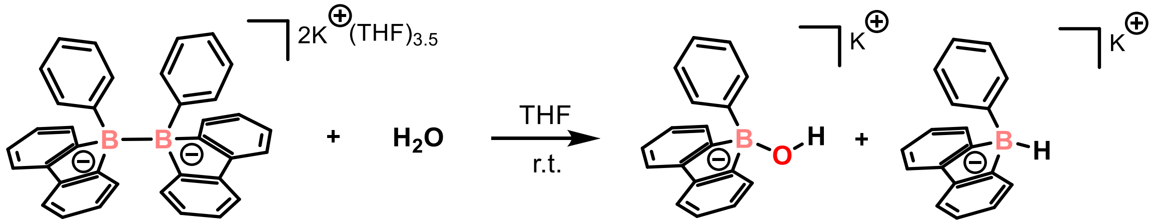

1 **3** **4**

43% **37%**

A 0.001 M H_2O-THF mixed solution was freshly prepared. In a 20 mL vial, **1** (50 mg, 0.059 mmol) was dissolved in 10 mL of THF, and 59 μL of the H_2O-THF (0.059 mmol) was added using a 100 μL pipette. Instantly, the solution changed from orange-red to pale yellow. After stirring overnight, all volatile components were removed under a high vacuum. Compounds **3** and **4** were purified by recrystallization based on slight differences in solubility. Colorless single crystals of **4** suitable for X-ray diffraction were obtained from a concentrated THF solution at -35℃, and crystals of **3** were then isolated from the solution with 1 eq. of 18-crown-6. Isolation yield: Compound **3·(18-C-6)**: 14.0 mg, 43%, Compound **4**: 6.2 mg, 37%.

Compound 3 • (18-Crown-6):

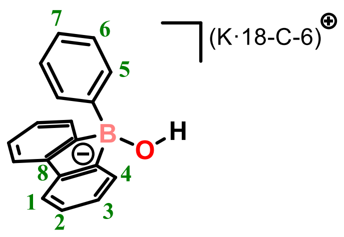

3·(18-C-6)

^{1}**H NMR** (600 MHz, THF-d_8) δ (ppm) = 7.52 - 7.47 (m, J = 7.4 Hz, 2H, *H*-1,3), 7.38 (t, *J* = 6.7 Hz, 2H, *H*-5), 6.87 - 6.82 (m, 6H, *H*-2, 6, 4), 6.75 (t, *J* = 7.3 Hz, 1H, *H*-7), 3.36 (s, 24H, *H*-18-C-6). (Ps. The B-OH proton was not resolved in the ^{1}H NMR spectrum, but was confirmed by the characteristic O-H stretching)
^{13}**C{^{1}H} NMR** (151 MHz, THF-d_8) δ (ppm) = 149.72 (*C*-8), 135.06 (*C*-5), 132.40 (*C*-3), 126.85 (*C*-6), 125.36 (*C*-4), 124.07 (*C*-2), 122.96 (*C*-7), 119.06 (*C*-1), 70.97 (*C*-18-Crown-6).
^{11}**B NMR** (193 MHz, THF-d_8) δ (ppm) = 1.56
IR (ATR, cm^{-1}): 3642 (sp. O-H stretching)
HRMS ($[C_{18}H_{14}BO]^-$): Simulated: 257.1138, Test: 257.1125.

Compound 4:

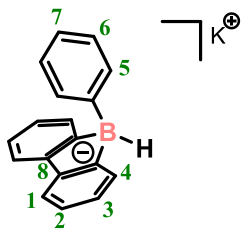

4

^{1}**H NMR** (600 MHz, THF-d_8) δ (ppm) = 7.58 (d, *J* = 7.4 Hz, 2H, *H-1*), 7.53 - 7.48 (m, 2H, *H*-3), 7.36 - 7.30 (m, 2H, *H*-5), 6.99 - 6.94 (m, 2H, *H*-2), 6.92 (t, *J* = 7.1 Hz, 2H, *H*-4), 6.90 - 6.86(m, 2H, *H*-6), 6.75 (t, *J* = 7.3 Hz, 1H, *H*-7), 3.04 (dd, *J* = 154.2, 77.0 Hz, 1H, *B-H*).
^{13}**C{^{1}H} NMR** (151 MHz, THF-d_8) δ (ppm) = 149.72 (*C*-8), 135.06 (*C*-5), 132.40 (*C*-3), 126.85 (*C*-6), 125.36 (*C*-4), 124.07 (*C*-2), 122.96 (*C*-7), 119.06 (*C*-1).
^{11}**B NMR** (193 MHz, THF-d_8) δ (ppm) = -11.55 (d, $J_{B\text{-}H}$ = 77.2 Hz)
HRMS ($[C_{18}H_{14}B]^-$): Simulated: 241.1189, Test: 241.1189.

Synthesis of Compound 5:

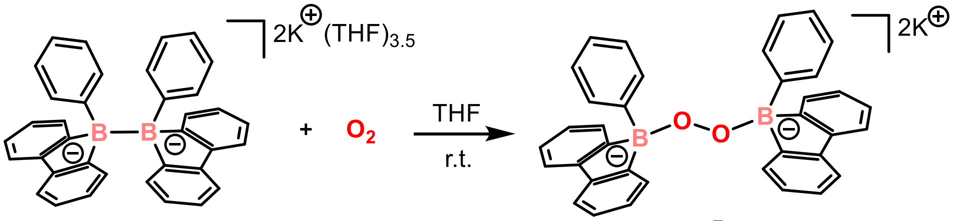

1 **5**

83%

A solution of **1** (50 mg, 0.059 mmol) in 10 mL of THF was degassed via three consecutive freeze-pump-thaw cycles in a 100 mL Schlenk tube. A dried atmosphere of O_2 was introduced to the tube using a three-way glass stopcock (tee joint). Immediately upon exposure to oxygen, the solution's color transformed from intense orange-red to colorless. After stirring for 0.5 hours, the ^{11}B NMR and ^{1}H NMR confirmed complete conversion to compound **5**. Colorless single crystals of **5** suitable for X-ray diffraction were obtained by slow evaporation of a concentrated THF solution containing 2 eq. of 18-crown-6. Isolation yield: Compound **5·(18-C-6)$_2$**: 54.6mg, 79%.

Compound 5·(18-Crown-6)$_2$:

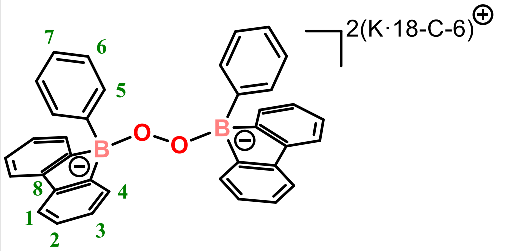

5·(18-C-6)$_2$

^{1}H NMR (600 MHz, THF-*d$_8$*) *δ* (ppm) = 7.43 (d, *J* = 7.2 Hz, 4H, *H*-1), 7.39 - 7.36 (m, 8H, *H*-5, 4), 6.92 - 6.89 (m, 4H, *H*-2), 6.88 - 6.85 (m, 8H, *H*-3, 6), 6.76 - 6.72 (m, 2H, *H*-7), 3.37 (s, 48H, *H*-18-Crown-6).
^{13}C{^{1}H} NMR (151 MHz, THF-*d$_8$*) *δ* (ppm) = 149.97 (*C*-8), 132.92 (*C*-5), 131.48 (*C*-4), 126.62 (*C*-6), 125.89 (*C*-3), 124.88 (*C*-2), 123.42 (*C*-7), 118.64 (*C*-1), 70.97 (*C*-18-Crown-6).
^{11}B NMR (193 MHz, THF-*d$_8$)* *δ* (ppm) = -1.12.
HRMS ($[C_{36}H_{26}B_2O_2]^{2-}$): Simulated: 512.2120, Test: 512.2375.

Thermodynamic stabilities of Compounds 2·(18-C-6) and 5·(18-C-6)$_2$:

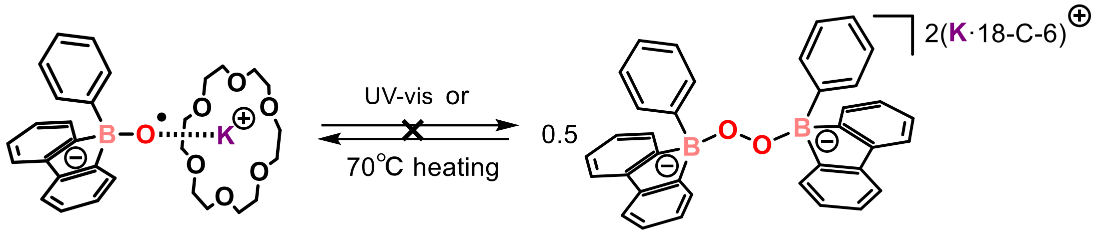

To test the stabilities of compounds **2·(18-C-6)** and **5·(18-C-6)$_2$**, we conducted reactions from both **2·(18-C-6)** and **5·(18-C-6)$_2$** as starting materials under heating at 70 °C and exposure to UV light at 254 nm and 365 nm. However, no corresponding transformations generated.

Transformation of Compound 2·(18-C-6) to 5·(2,2,2-cryptand)$_2$:

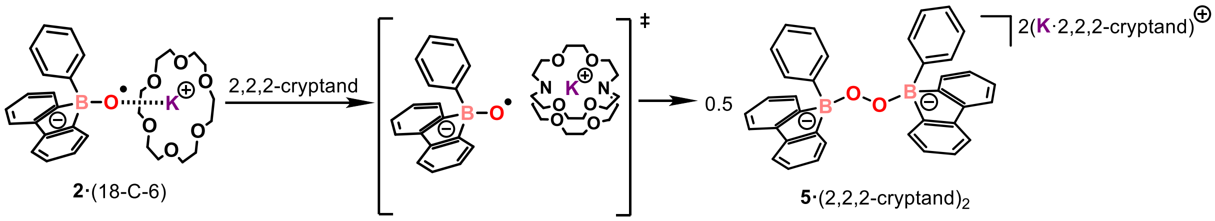

In a 20 mL vial, **2·(18-C-6)** (32.0 mg, 0.057 mmol) was dissolved in 10 mL of THF, 2,2,2-cryptand (43.1mg, 0.114 mmol) was added. After stirring overnight, the ^{11}B NMR showed the full conversion from **2·(18-C-6) to 5·(2,2,2-cryptand)$_2$**. Isolation yield: Compound **5·(2,2,2-cryptand)$_2$**: 34.2mg, 86%.

The controlled experiment was recorded by in situ ^{11}B NMR (Figure S25).

Synthesis of Compound 6a, 6b, and 7:

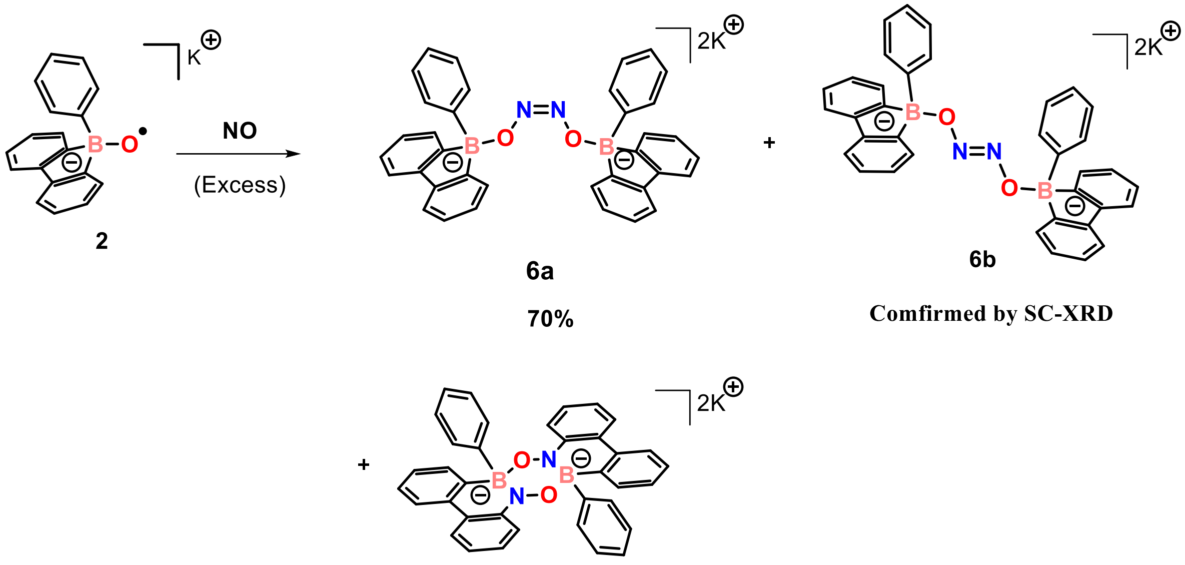

2 6a 70% 6b Comfirmed by SC-XRD

7

Comfirmed by SC-XRD

In a 100 mL Schlenk tube, a solution of **2** (30 mg, 0.100 mmol) in 10 mL of tetrahydrofuran (THF) was subjected to three consecutive freeze-pump-thaw cycles to remove dissolved gases. Subsequently, a dry nitric oxide (NO) atmosphere was introduced into the tube using a three-way glass stopcock (tee joint) under room temperature. Upon exposure to an excess of NO, the color of the solution rapidly changed from yellow to pale yellow. This reaction predominantly yielded a mixture of boronic ester-substituted azo compounds (**6a** as the major product and **6b** as the minor product) along with a small amount of 9-aza-10-boraphenanthrene derivative **7**. Crystals of **6a** were isolated from the mixture by carefully adjusting the solvent polarity through the dropwise addition of hexane. Light yellow single crystals of **6b** and **7,** suitable for X-ray diffraction analysis, were initially obtained by precipitating from a concentrated THF solution at -35 °C. Isolation yield: Compound **6a**, 21.8mg, 70%.

Compound 6a:

^{1}H NMR (600 MHz, THF-*d$_8$*) δ (ppm) = 7.49 (d, *J* = 7.5 Hz, 2H, *H*-1), 7.43 - 7.36 (m, 4H, *H*-5, 6), 6.99 (t, *J* = 7.3 Hz, 2H, *H*-2), 6.94 (t, *J* = 7.5 Hz, 2H, *H*-3), 6.90 (t, *J* = 7.0 Hz, 2H, *H*-4), 6.84 (t, *J* = 6.6 Hz, 1H, *H*-7).

^{13}C{^{1}H} NMR (151 MHz, THF-*d$_8$*) δ (ppm) = 150.12 (*C*-8), 133.57 (*C*-6), 131.61 (*C*-5), 127.21 (*C*-3), 126.20 (*C*-4), 125.97 (*C*-2), 124.47 (*C*-7), 119.13 (*C*-1).

^{11}B NMR (193 MHz, THF-*d$_8$*) δ (ppm) = 4.03.

HRMS($[C_{36}H_{26}B_2O_2N_2]^{2-}+NH_4^{+}$): Simulated: 558.2524, Test: 558.2525.

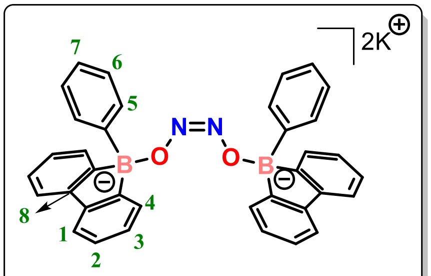

6a

Reaction between 2 and nBu$_3$SnH:

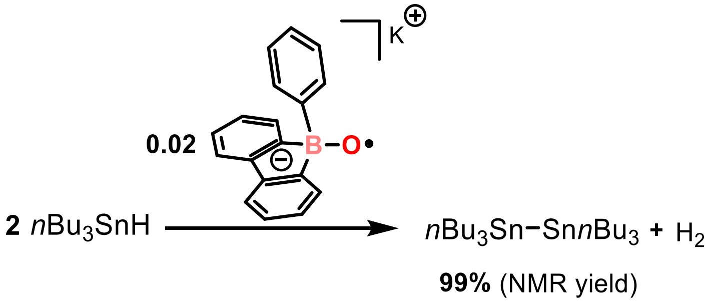

nBu$_3$SnH (800.0 mg, 2.75 mmol) and **2**·(18-Crown-6) (1.6 mg, 0.027 mmol) were dissolved in THF-d_8 (0.8 mL) within a J-Young NMR tube. Once these two species were mixed, dihydrogen bubbles were released immediately. After 2 hours, in situ ^{1}H NMR, ^{13}C NMR, and ^{119}Sn NMR spectra confirmed the complete conversion of nBu$_3$SnH to the coupling product nBu$_3$Sn-SnnBu$_3$, along with the generation of dihydrogen. NMR yield: nBu$_6$Sn$_2$: 99%.

Product: ***n*Bu$_6$Sn$_2$**:

^{1}H NMR (400 MHz, THF-d_8) δ (ppm) = 1.64 - 1.45 (m, 12H), 1.43 - 1.26 (m, 12H), 1.15 - 0.98 (m, 12H), 0.91 (t, J = 7.3 Hz, 18H).

^{13}C{^{1}H} NMR (151 MHz, THF-d_8): δ (ppm) = 31.95, 28.73, 14.42, 11.06.

^{119}Sn{^{1}H} NMR (149 MHz, THF-d_8): δ (ppm) = -83.69.

All spectroscopic data are in accordance with those reported in the literature [3].

Controlled experiment between 3 and nBu$_3$SnH:

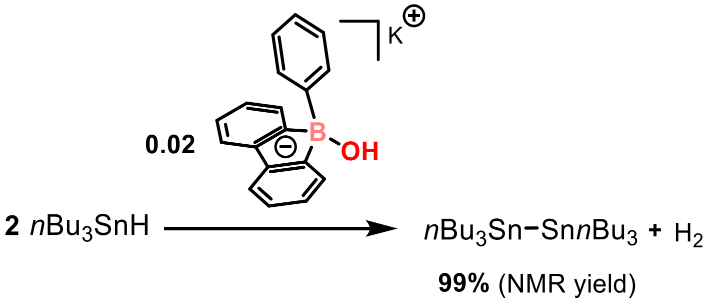

nBu$_3$SnH (800.0 mg, 2.75 mmol) and **3**·(18-Crown-6) (2.2 mg, 0.027 mmol) were dissolved in THF-d_8 (0.8 mL) within a J-Young NMR tube. Once these two species were mixed, dihydrogen bubbles were released immediately. After 2 hours, in situ ^{1}H NMR, ^{13}C NMR, and ^{119}Sn NMR spectra confirmed the complete conversion of nBu$_3$SnH to the coupling product nBu$_3$Sn-SnnBu$_3$, along with the generation of dihydrogen. NMR yield: nBu$_6$Sn$_2$: 99%.

Product: ***n*Bu$_6$Sn$_2$**:

^{1}H NMR (400 MHz, THF-d_8) δ (ppm) = 1.62 - 1.47 (m, 12H), 1.37 - 1.26 (m, 12H), 1.08 - 0.95 (m, 12H), 0.91 (t, J = 7.3 Hz, 18H).

^{13}C{^{1}H} NMR (151 MHz, THF-d_8): δ (ppm) = 31.81, 28.60, 14.30, 11.15.

The product is the same as the reported in the literature[3].

Proposed mechanism for Sn-Sn Coupling：

Based on our experimental observations and control reactions, we propose a plausible radical-mediated pathway for the Sn-Sn coupling, and have supplemented detailed mechanistic insights and supporting data：

The boryloxyl radical compound **2** (BO·) acts as the active catalytic species, and the catalytic cycle may proceed as follows: First, BO· abstracts a hydrogen atom from *n*Bu_3SnH to generate the tributyltin radical (*n*Bu_3Sn·) and boron hydroxide intermediate compound **3** (BOH). Second, two equivalents of *n*Bu_3Sn· undergo homolytic coupling to form the target Sn-Sn product (*n*Bu_3Sn-Sn-*n*Bu_3). Crucially, control experiments confirm that compound **3** (BOH) cannot regenerate compound **2** (BO·) via spontaneous dehydrogenation. Instead, compound **3** must undergo a hydrogen atom transfer (HAT) reaction with another equivalent of nBu_3SnH to regenerate the active radical **2**, concurrently producing a new *n*Bu_3Sn· radical. The H_2 gas observed as bubbles during the experiment originates from the combination of the two hydrogen atoms abstracted from *n*Bu_3SnH in these steps.

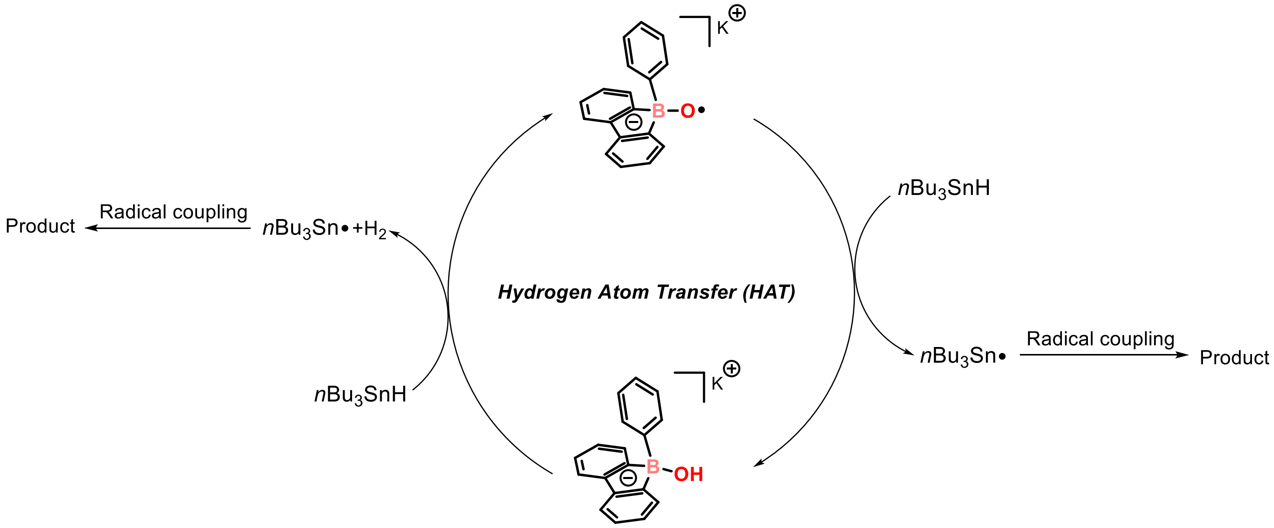

Scheme S1. Proposed mechanism of the catalytic Sn-Sn coupling cycle

To validate this mechanism, we performed a series of control experiments:

(1) A reaction using only BOH failed to generate BO·, confirming that BOH cannot undergo spontaneous dehydrogenation without *n*Bu_3SnH.

(2) Mixing 1 equivalent of compound **3** (BOH) with 100 equivalents of *n*Bu_3SnH yielded the Sn-Sn product in a yield comparable to that of the catalytic reaction. Based on the NMR data, we cannot confirm that catalyst remains intact after the completion of the catalytic reaction.

(3) Controlled experiments between **3** and *n*Bu_3SnH (Figure S37).

NMR Spectra:

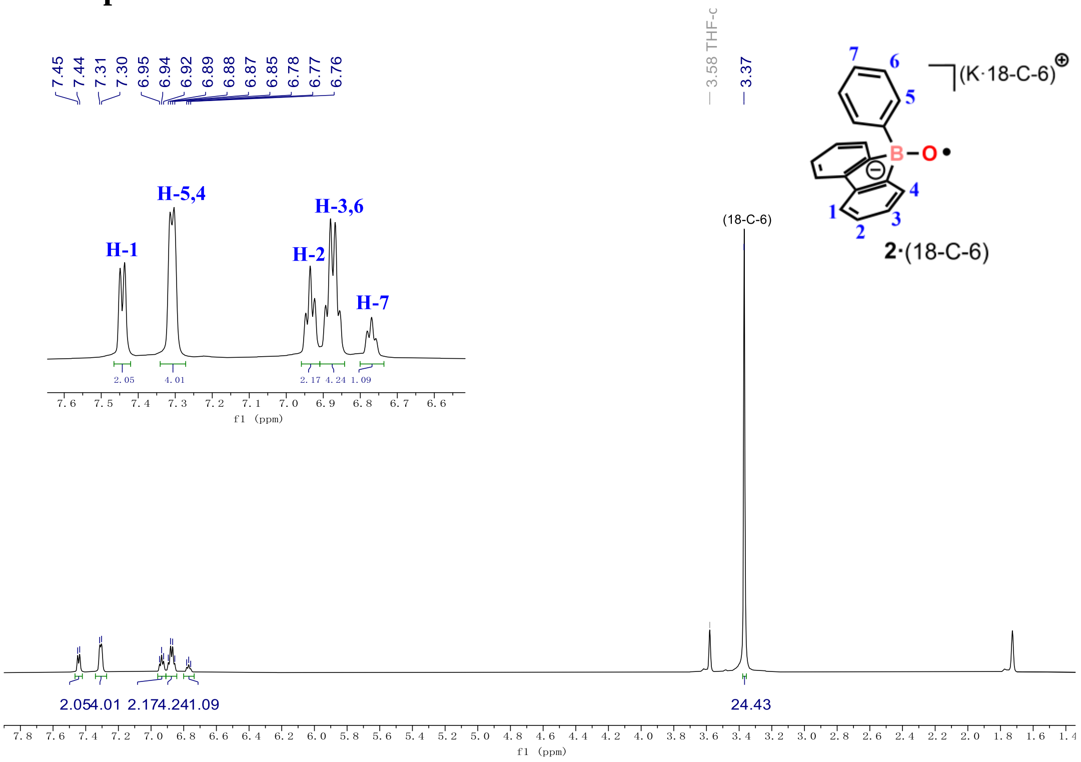

Figure S1. ^{1}H NMR spectrum of **2**·(18-C-6) (^{1}H: 600 MHz, THF-d_8)

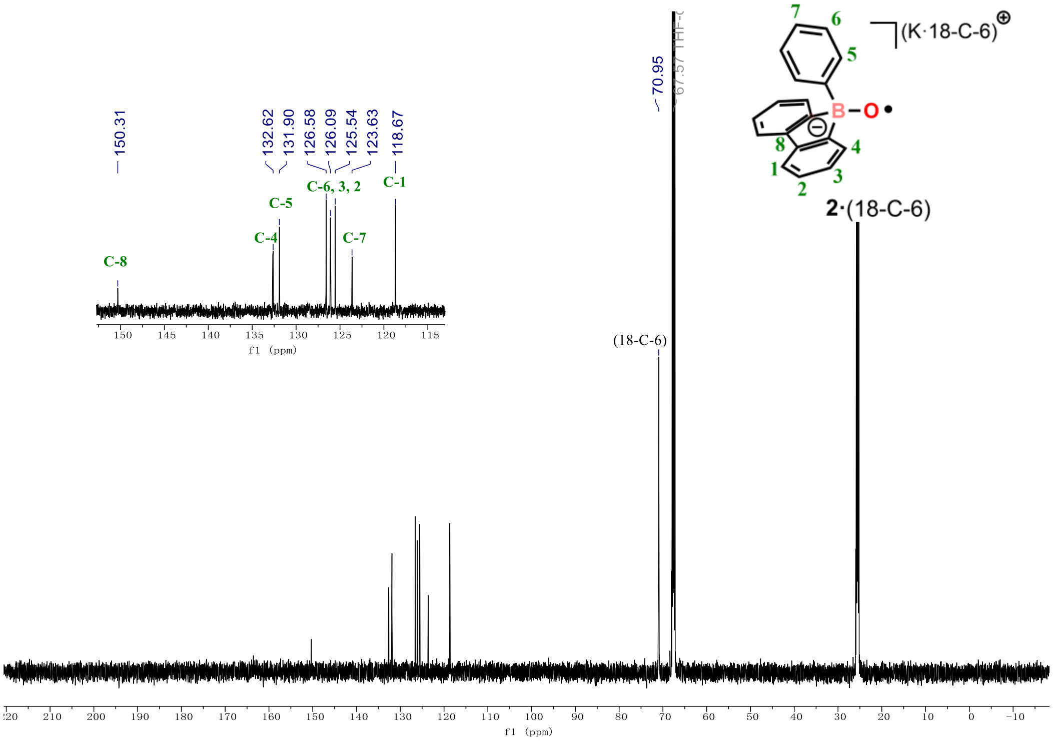

Figure S2. $^{13}C\{^{1}H\}$ NMR spectrum of **2**·(18-C-6) (^{13}C: 151 MHz, THF-d_8)

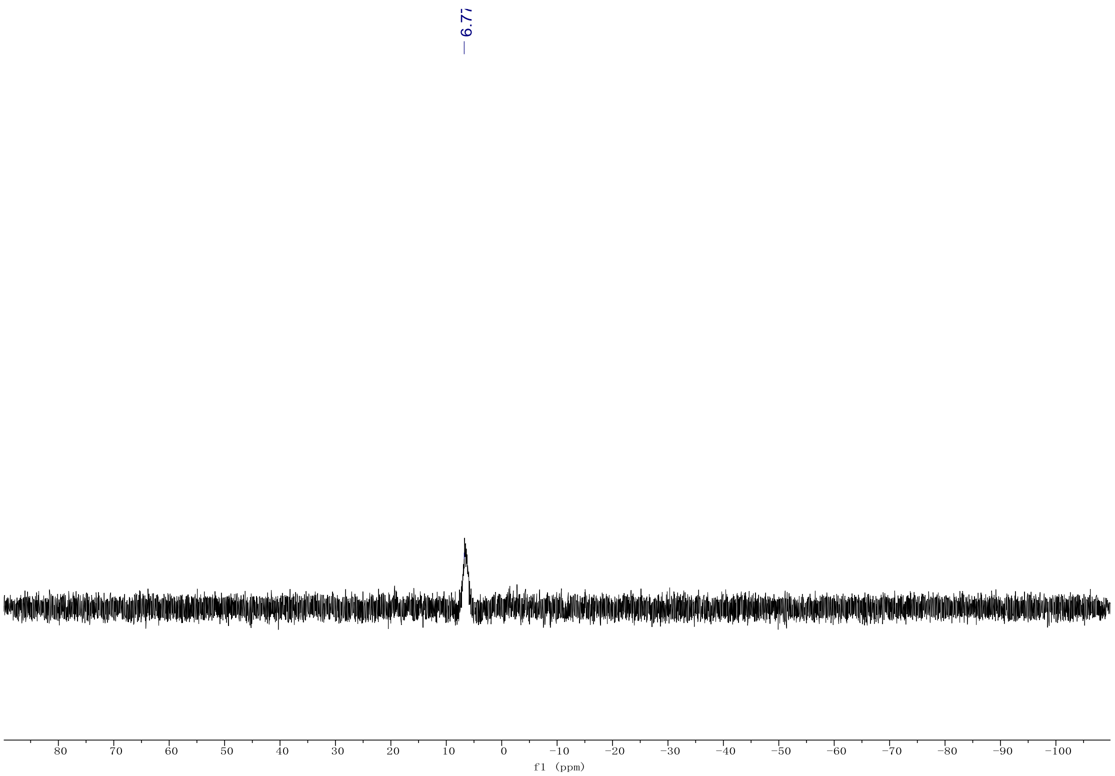

Figure S3. ^{11}B NMR spectrum of **2·**(18-C-6) (^{11}B: 193 MHz, THF-d_8)

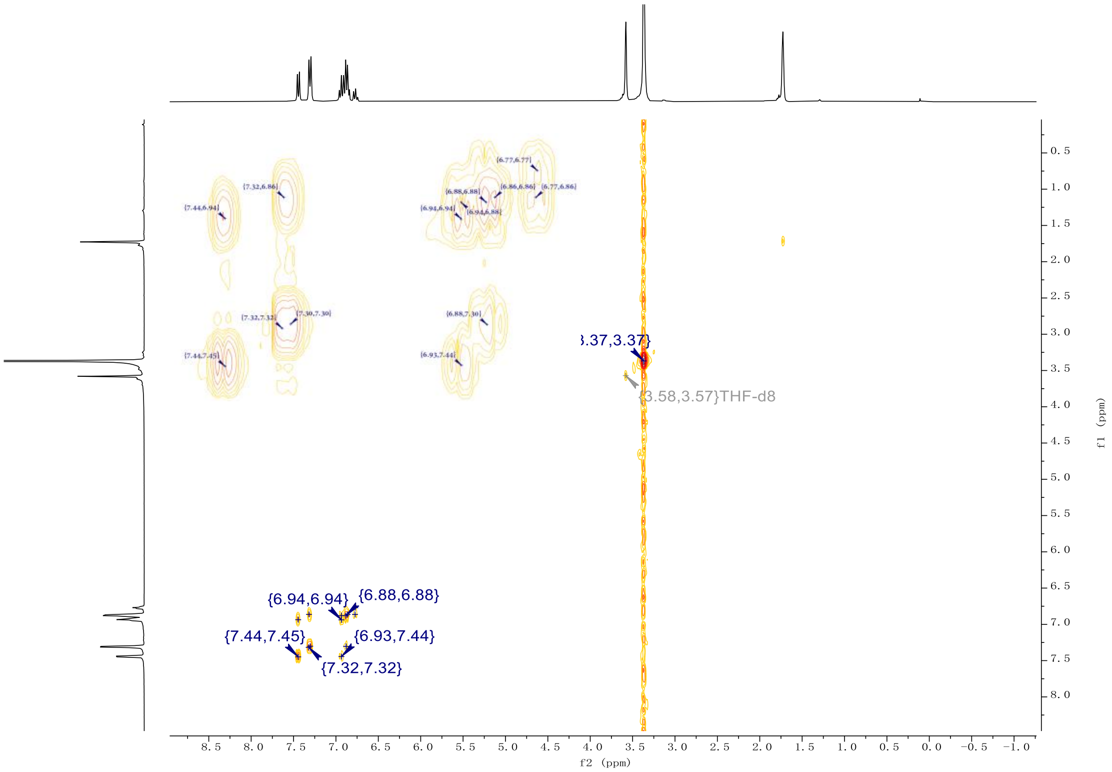

Figure S4. ^{1}H-^{1}H COSY NMR spectrum of **2**·(18-C-6) (^{1}H: 600 MHZ, THF-d_8)

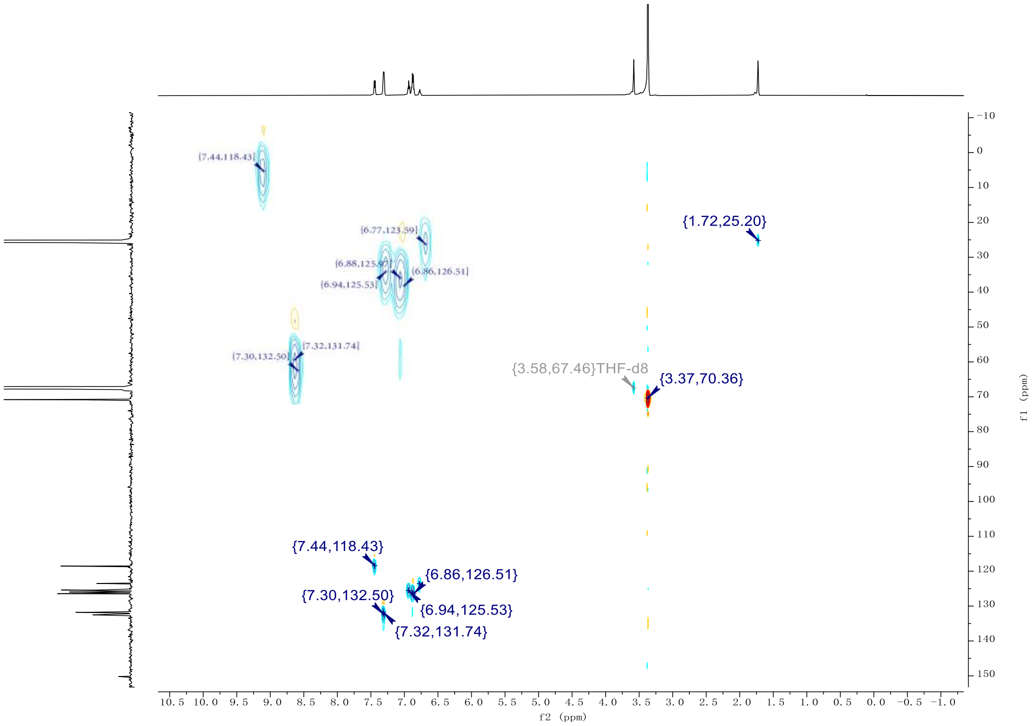

Figure S5. ^{1}H-^{13}C HSQC NMR spectrum of **2**·(18-C-6) (^{1}H: 600 MHz and ^{13}C: 151 MHz, THF-d_8)

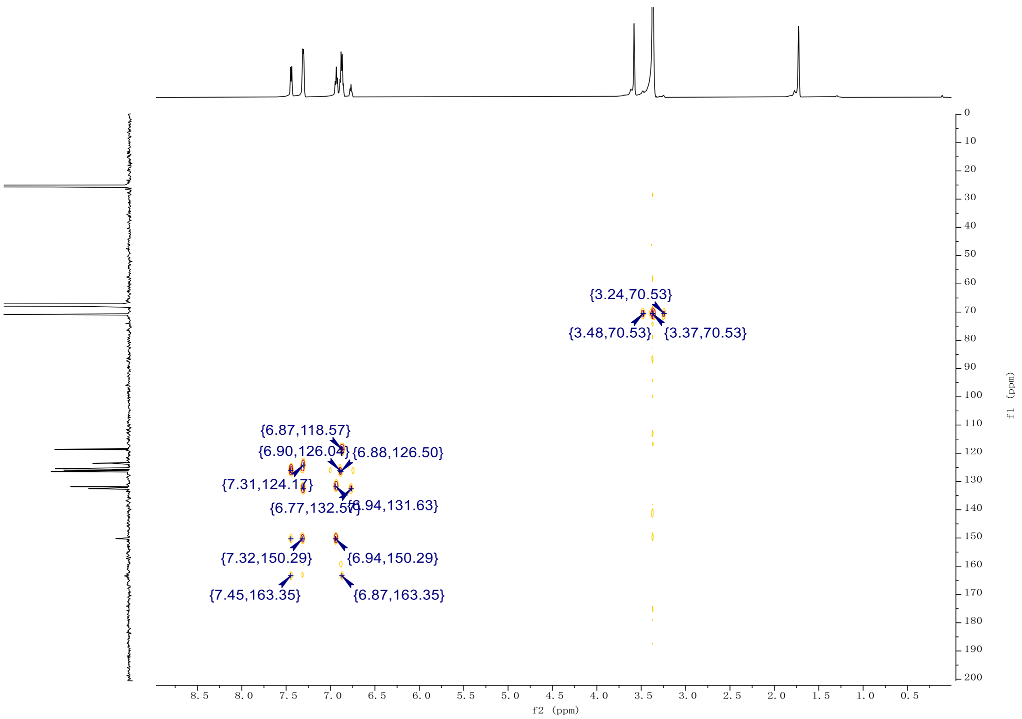

Figure S6. ^{1}H-^{13}C HMBC NMR spectrum of **2**·(18-C-6) (^{1}H: 600 MHz and ^{13}C: 151 MHz, THF-d_8)

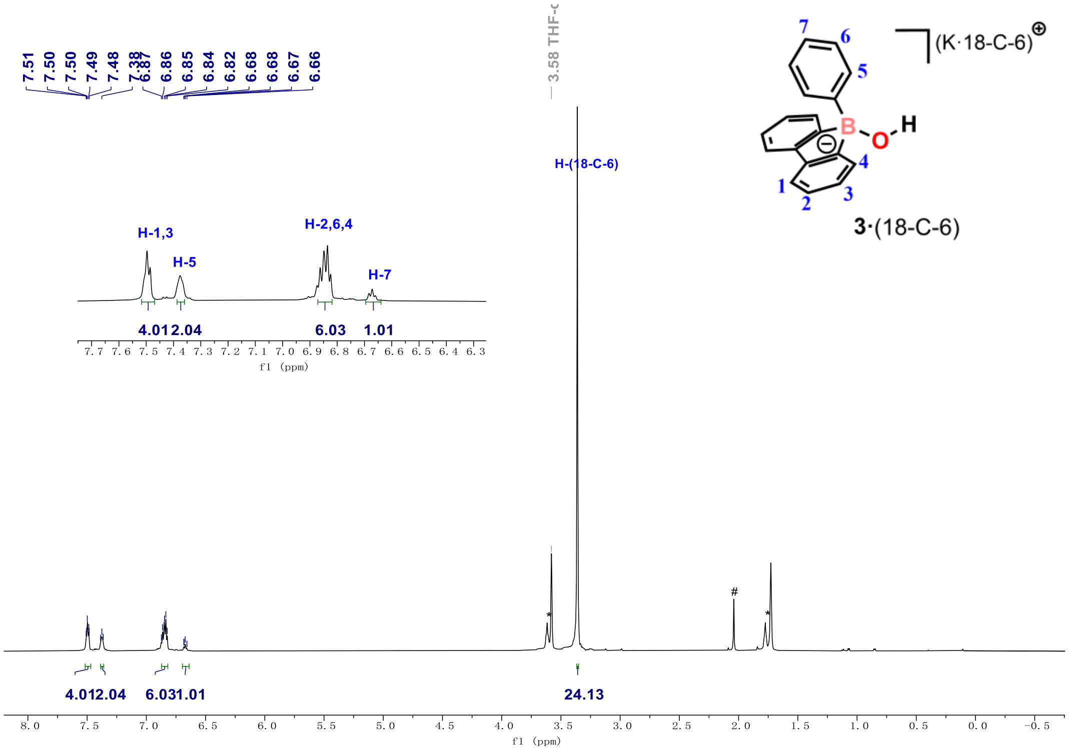

Figure S7. ^{1}H NMR spectrum of **3**·(18-C-6) (^{1}H: 600 MHz, THF-d_8)(*: THF, #: acetone from THF-d_8)

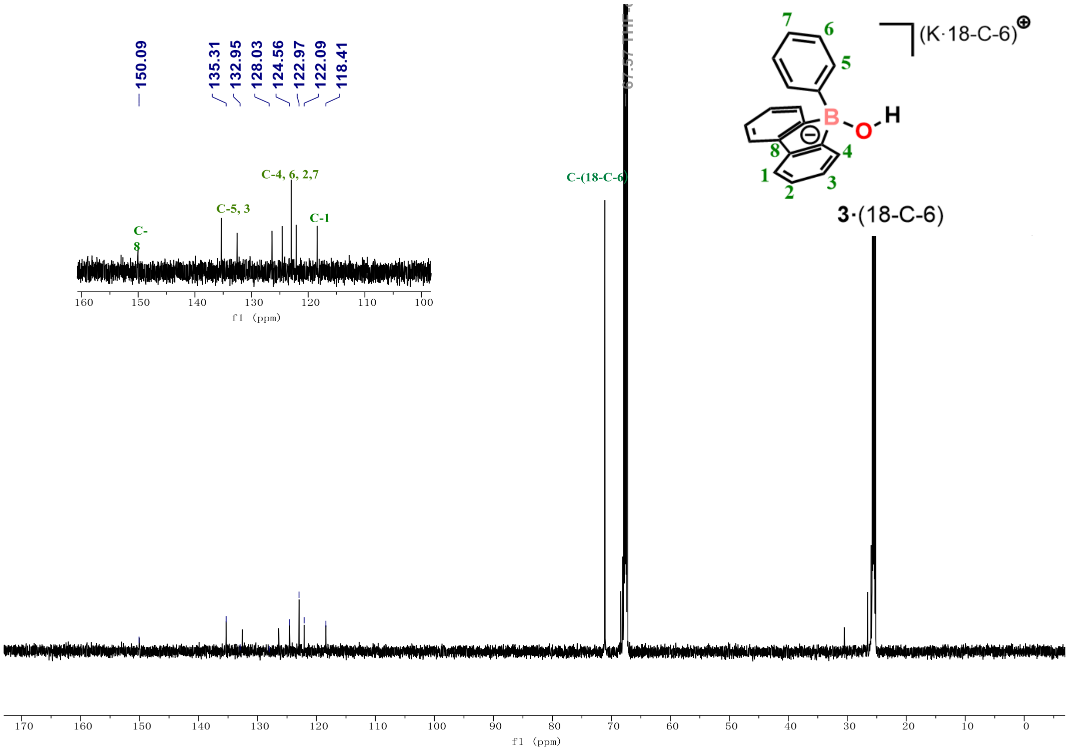

Figure S8. ^{13}C{^{1}H} NMR spectrum of **3**·(18-C-6) (^{13}C: 150 MHz, THF-d_8)

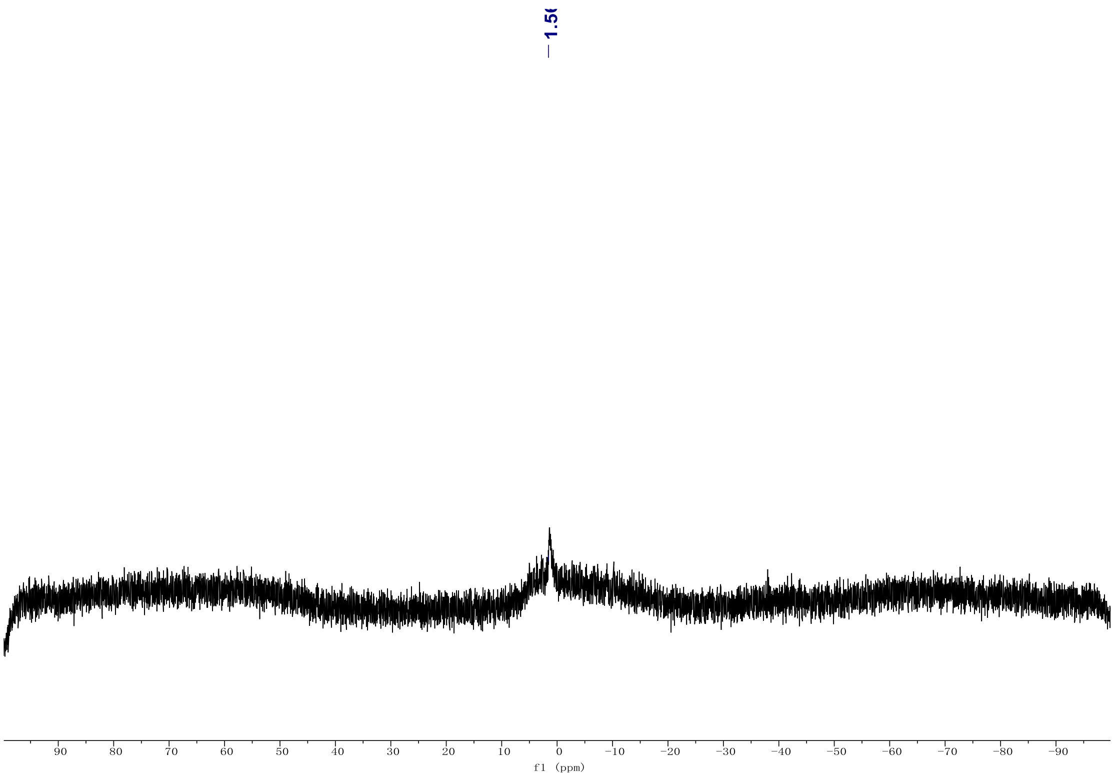

Figure S9. ^{11}B NMR spectrum of **3**·(18-C-6) (^{11}B: 193 MHz, THF-d_8)

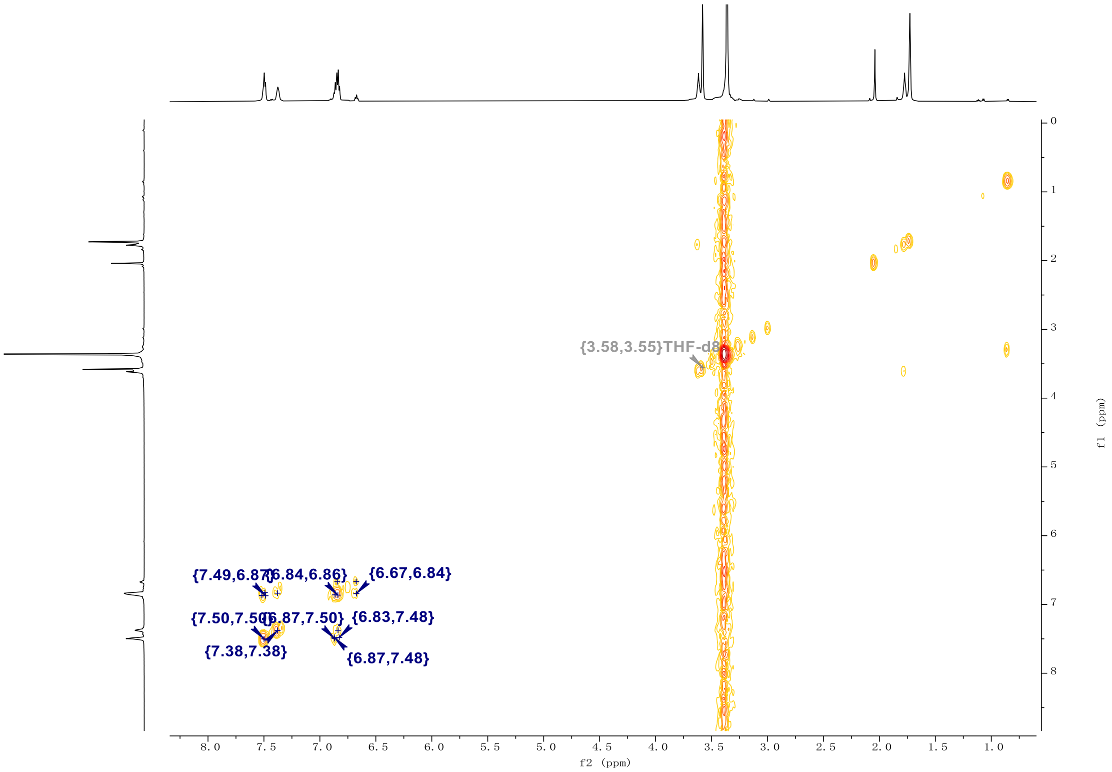

Figure S10. ^{1}H-^{1}H COSY NMR spectrum of **3**·(18-C-6) (^{1}H: 600 MHZ, THF-d_8)

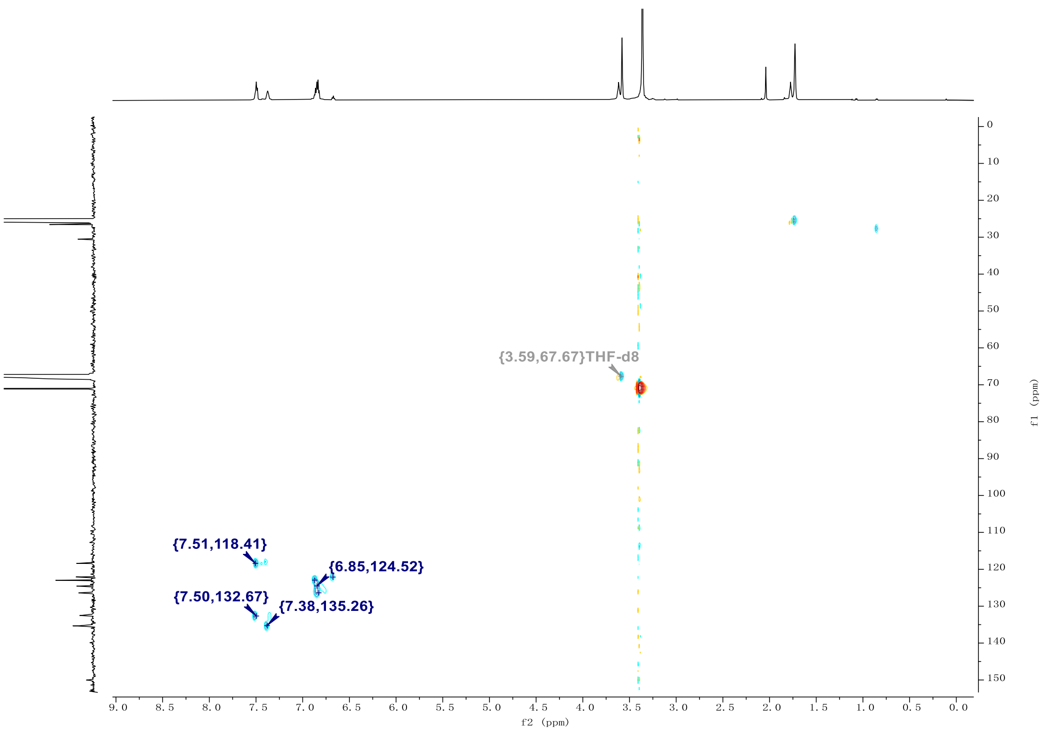

Figure S11. ^{1}H-^{13}C HSQC NMR spectrum of **3**·(18-C-6) (^{1}H: 600 MHz and ^{13}C: 151 MHz, THF-d_8)

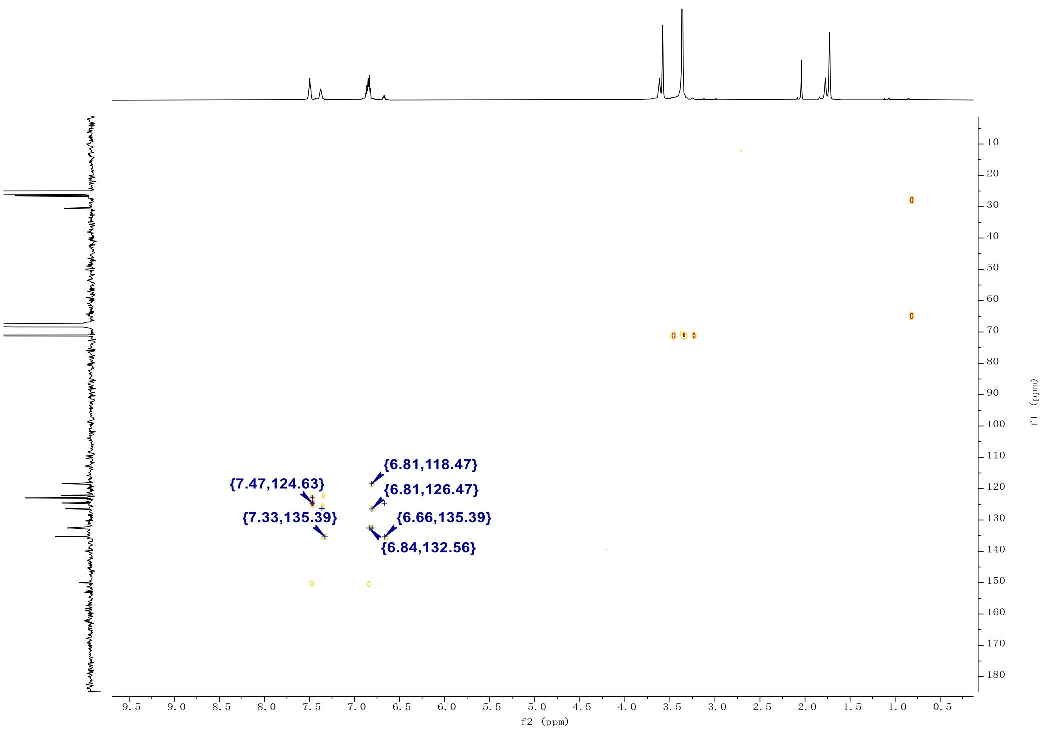

Figure S12. ^{1}H-^{13}C HMBC NMR spectrum of **3**·(18-C-6) (^{1}H: 600 MHz and ^{13}C: 151 MHz, THF-d_8)

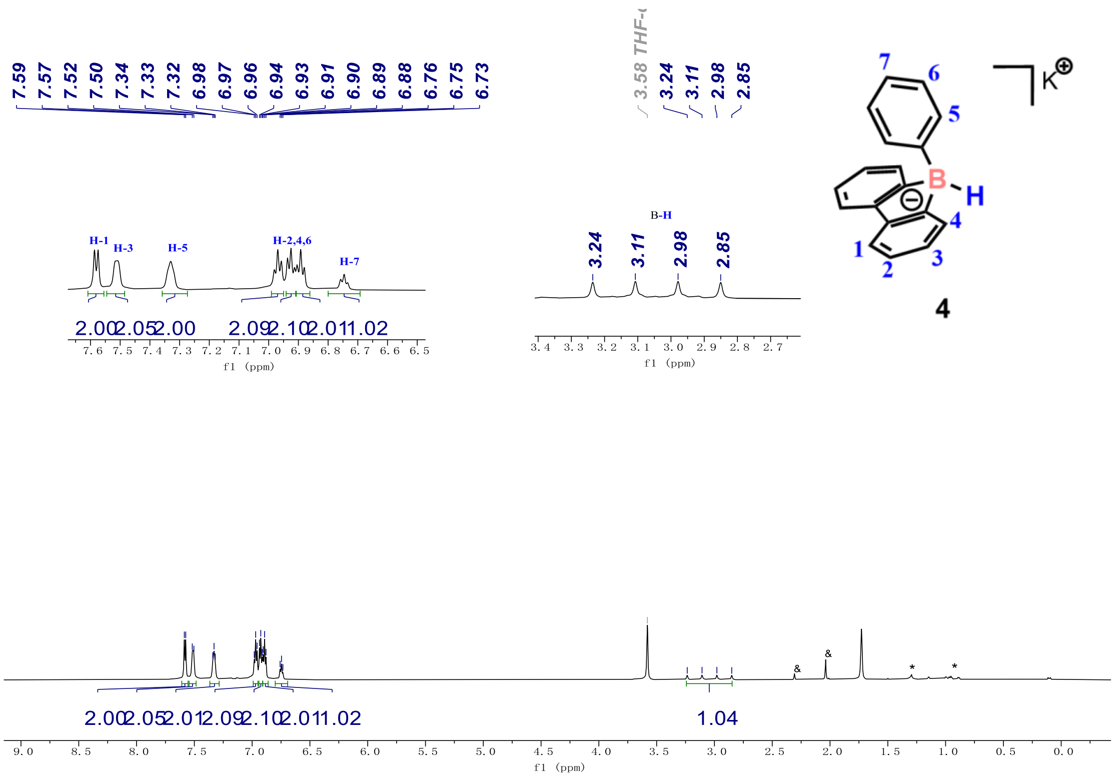

Figure S13. ^{1}H NMR spectrum of **4** (^{1}H: 600 MHz, THF-d_8) (&,*: impurity from *d*-solvent)

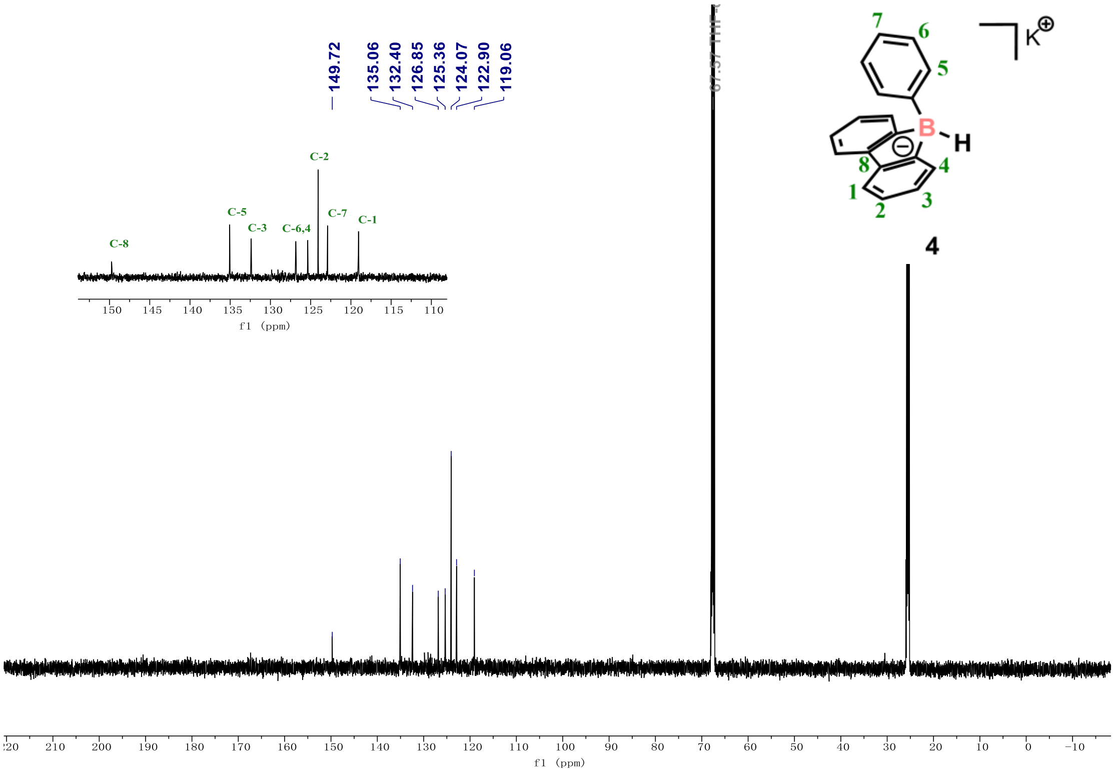

Figure S14. ^{13}C{^{1}H} NMR spectrum of **4** (^{13}C: 151 MHz, THF-d_8)

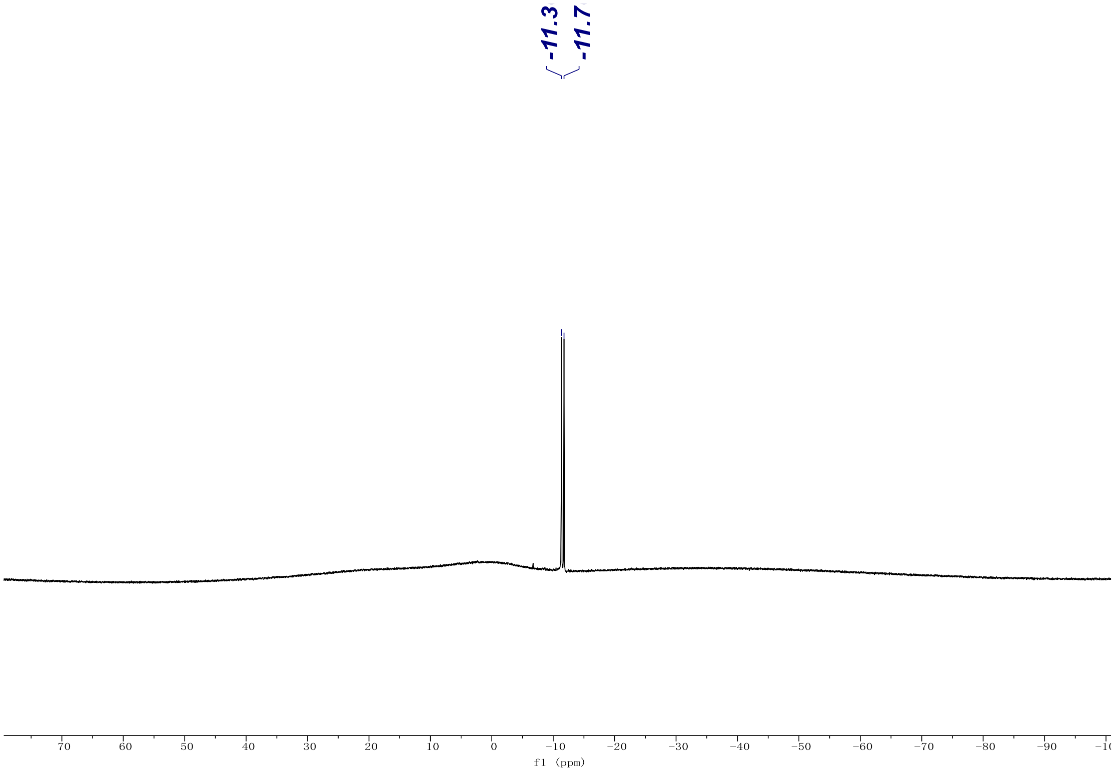

Figure S15. ^{11}B NMR spectrum of **4** (^{11}B: 193 MHZ, THF-d_8)

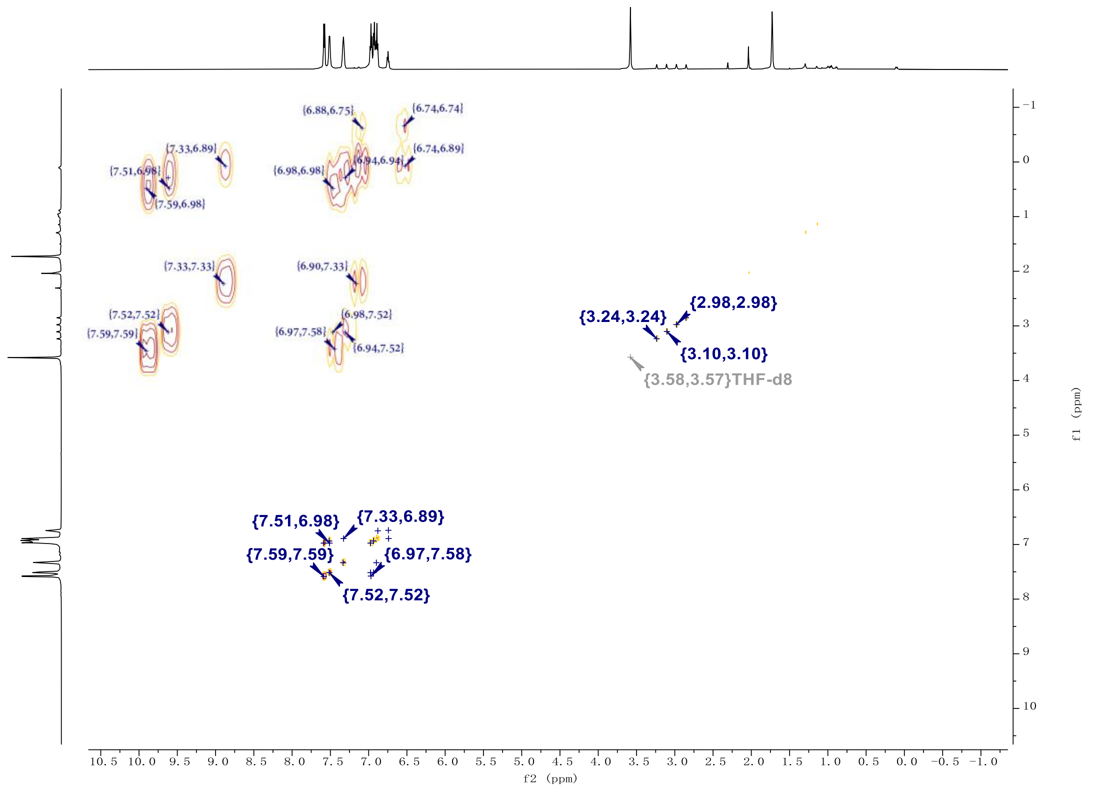

Figure S16. ^{1}H-^{1}H COSY NMR spectrum of **4** (^{1}H: 600 MHZ, THF-d_8)

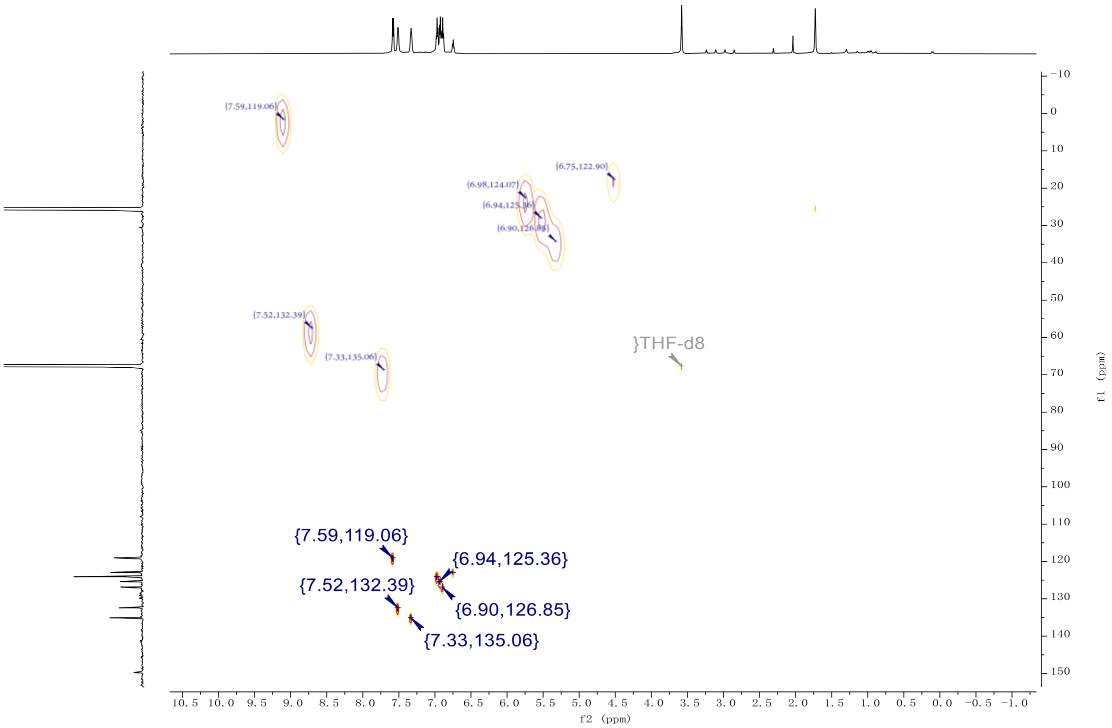

Figure S17. ^{1}H-^{13}C HSQC NMR spectrum of **4** (^{1}H: 600 MHz and ^{13}C: 151 MHz, THF-d_8)

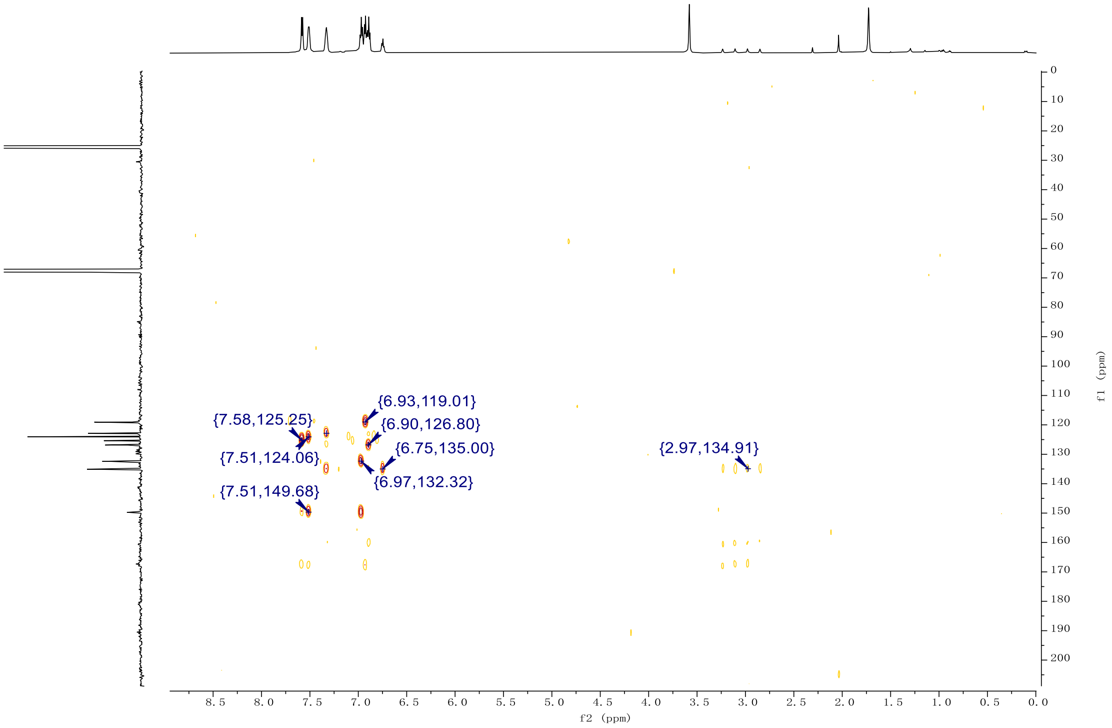

Figure S18. ^{1}H-^{13}C HMBC NMR spectrum of **4** (^{1}H: 600 MHz and ^{13}C: 151 MHz, THF-d_8)

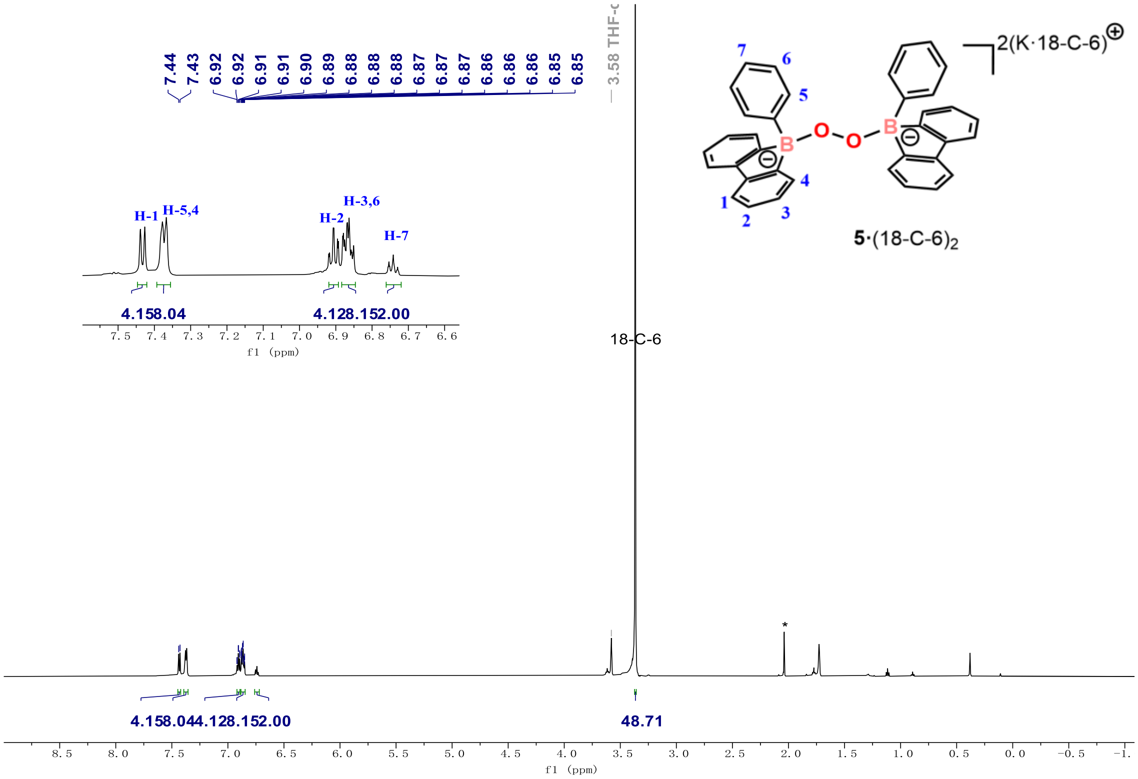

Figure S19. ^{1}H NMR spectrum of **5**·(18-C-6)$_2$ (^{1}H: 600 MH$_Z$, THF-d_8) (#: acetone from THF-d_8)

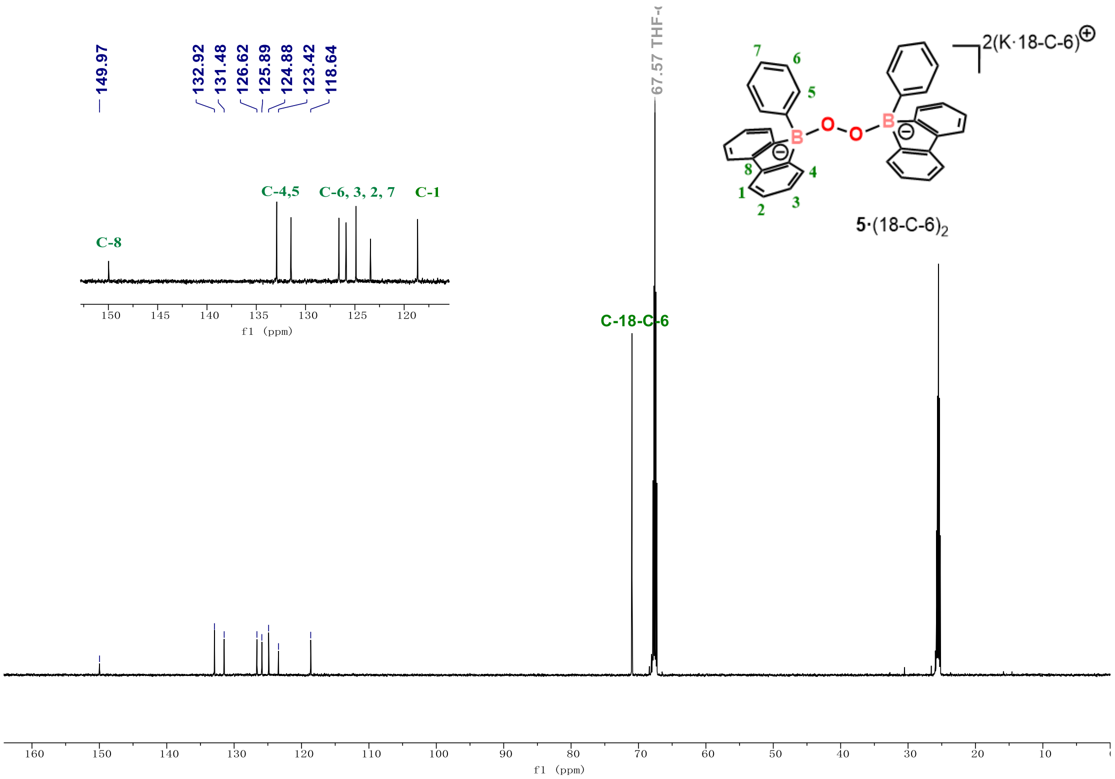

Figure S20. ^{13}C{^{1}H} NMR spectrum of **5**·(18-C-6)$_2$ (^{13}C: 151 MH$_Z$, THF-d_8)

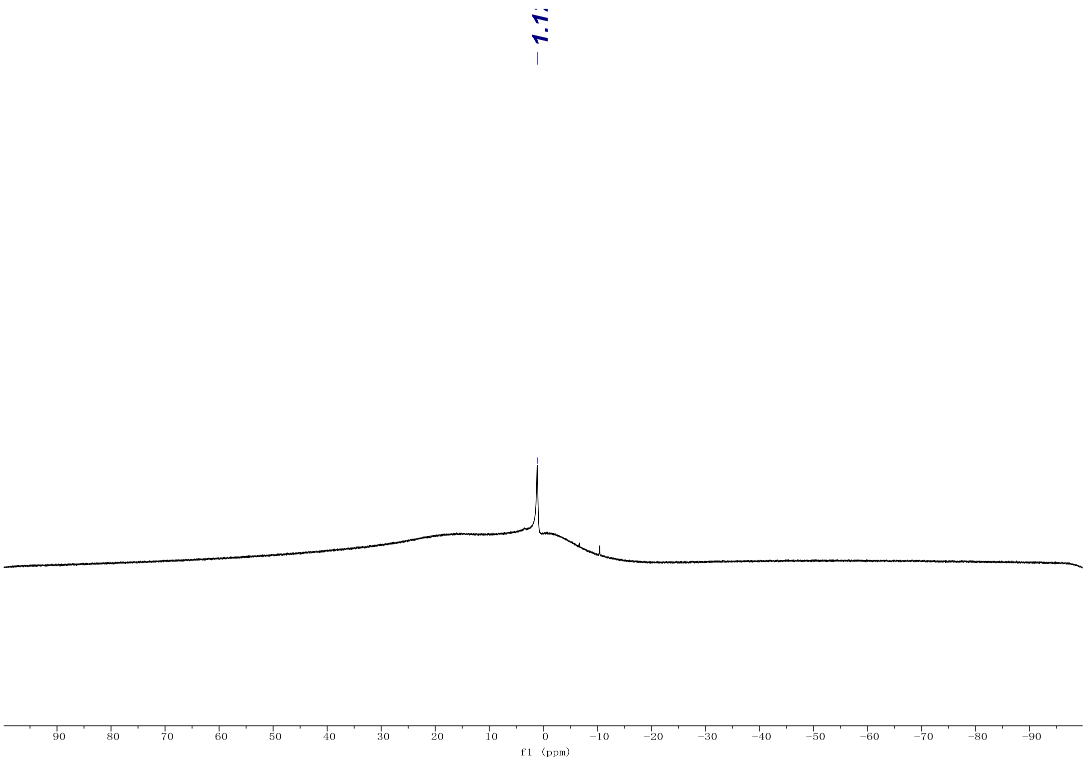

Figure S21. ^{11}B NMR spectrum of **5**·(18-C-6)$_2$ (^{11}B: 193 MHZ, THF-d_8)

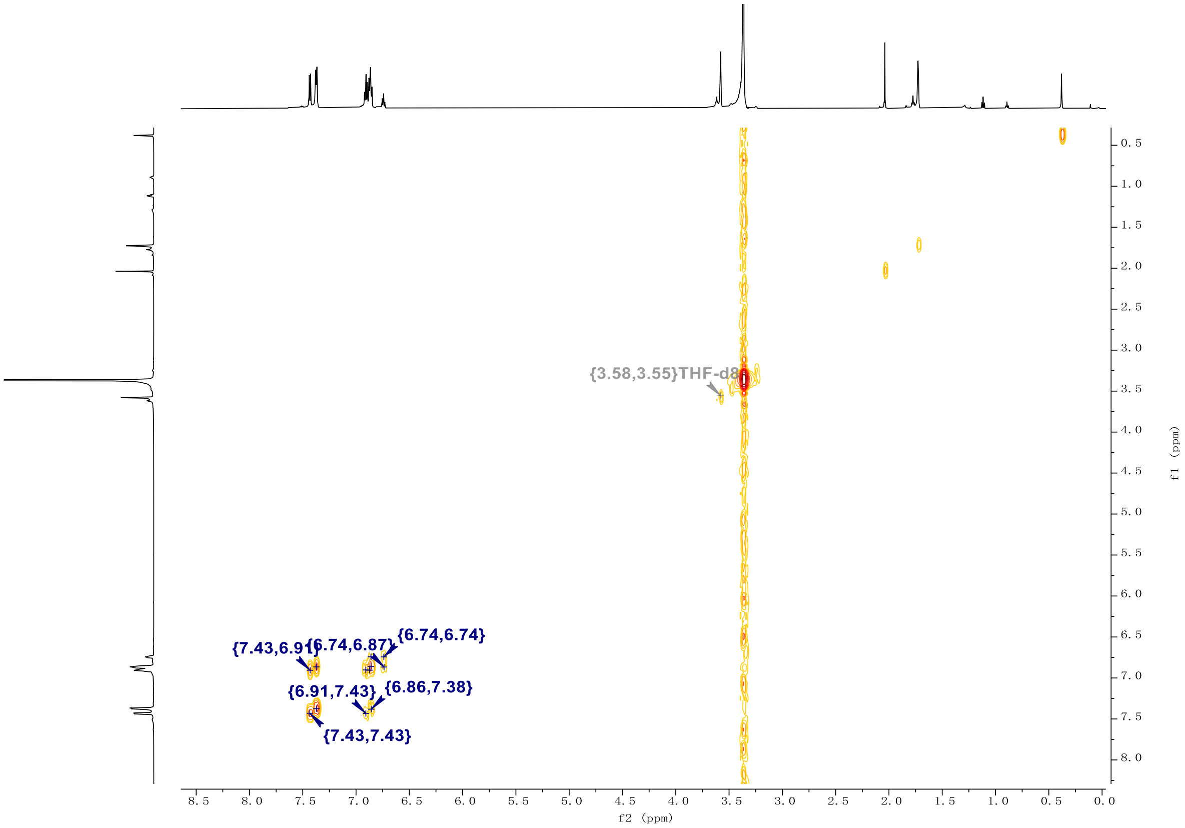

Figure S22. ^{1}H-^{1}H COSY NMR spectrum of **5**·(18-C-6)$_2$ (^{1}H: 600 MHZ, THF-d_8)

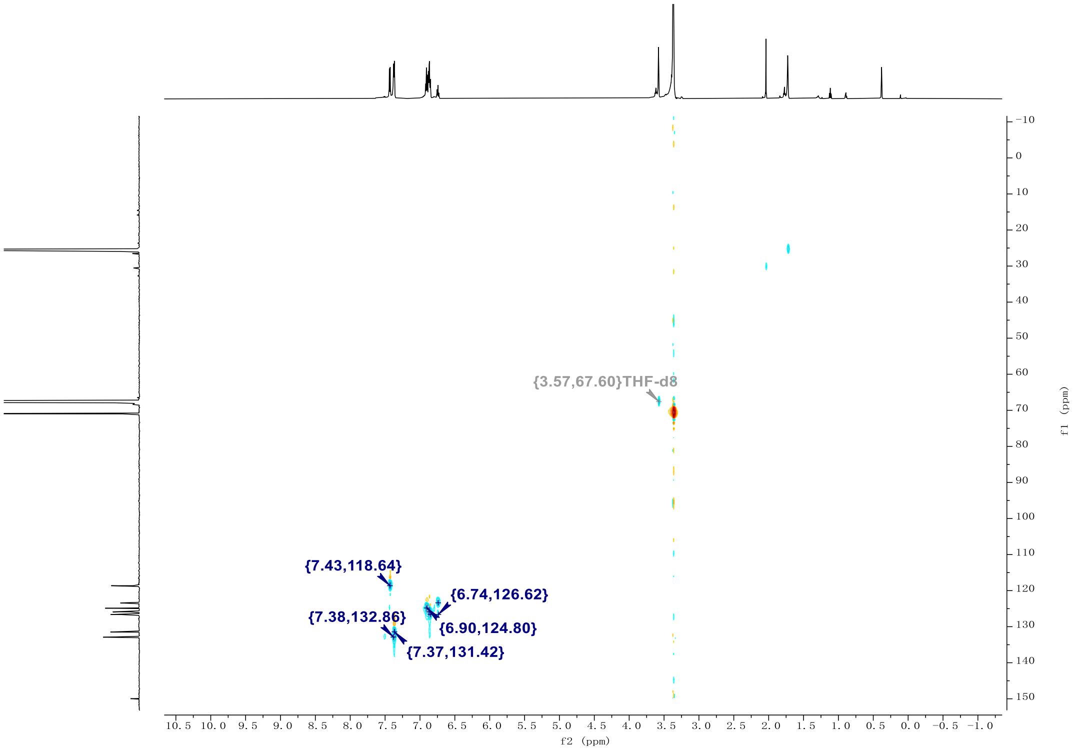

Figure S23. ^{1}H-^{13}C HSQC NMR spectrum of **5**·(18-C-6)$_2$ (^{1}H: 600 MHz and ^{13}C: 151 MHz, THF-d_8)

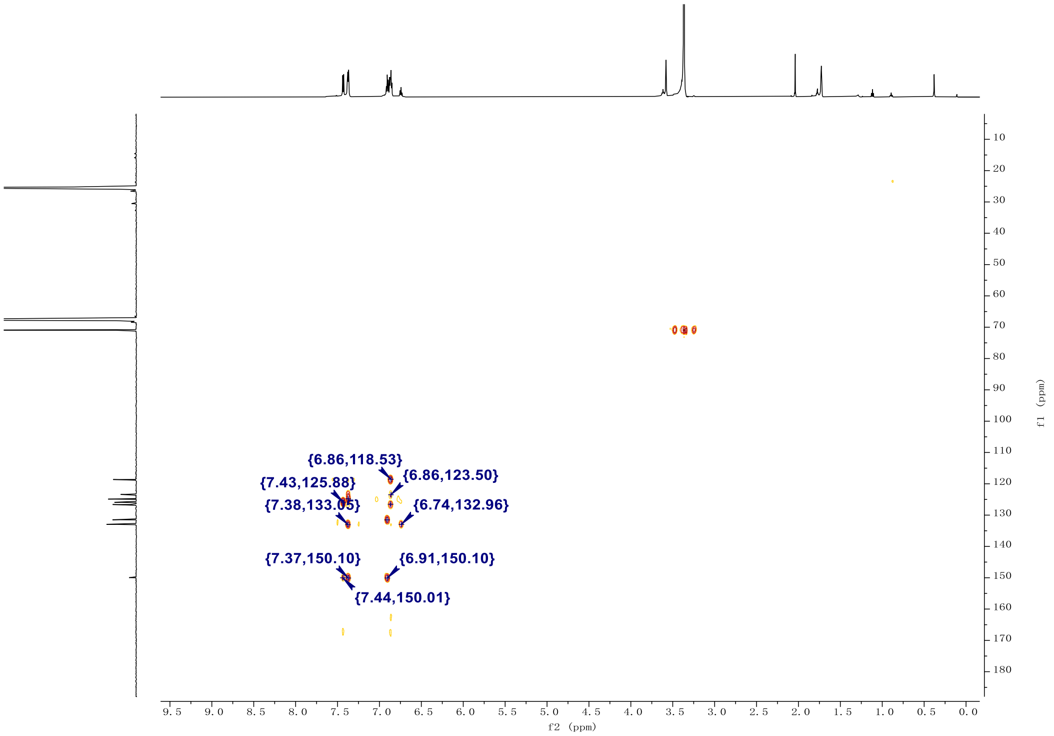

Figure S24. ^{1}H-^{13}C HMBC NMR spectrum of **5**·(18-C-6)$_2$ (^{1}H: 600 MHz and ^{13}C: 151 MHz, THF-d_8)

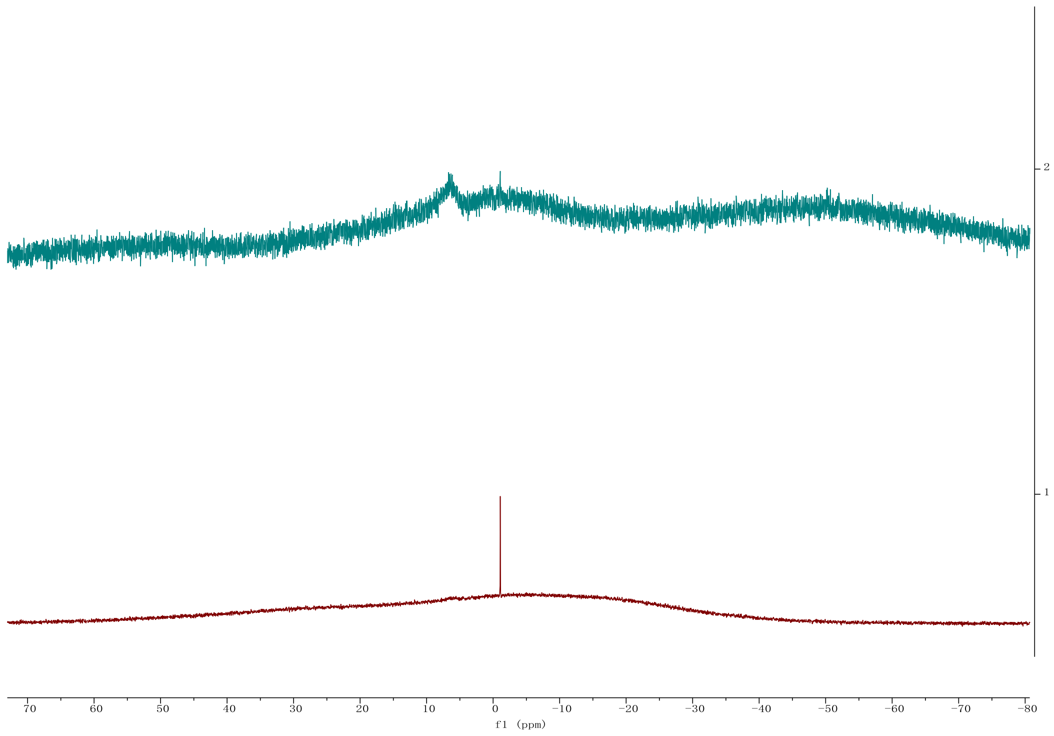

Figure S25. In situ ^{11}B NMR spectra of the transformation from 2·(18-C-6) to 5·(2,2,2-cryptand)$_2$. (^{11}B: 193 MHZ, THF-d_8)

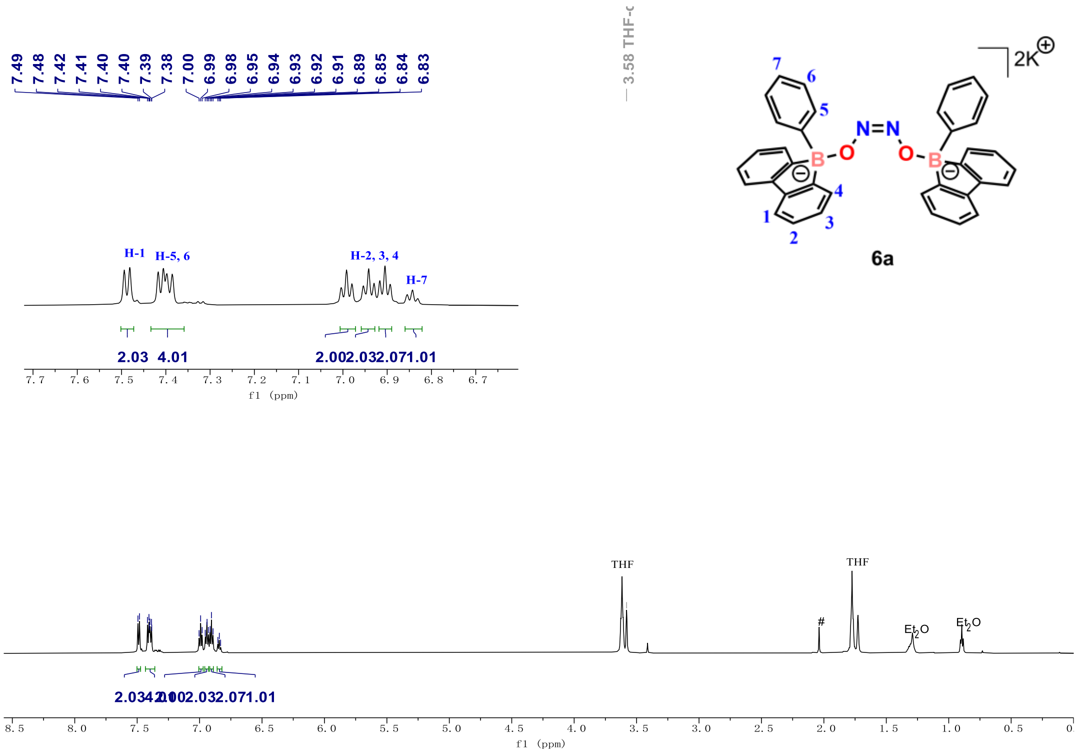

Figure S26. ^{1}H NMR spectrum of **6a** (^{1}H: 600 MHz, THF-d_8) (#: acetone from THF-d_8)

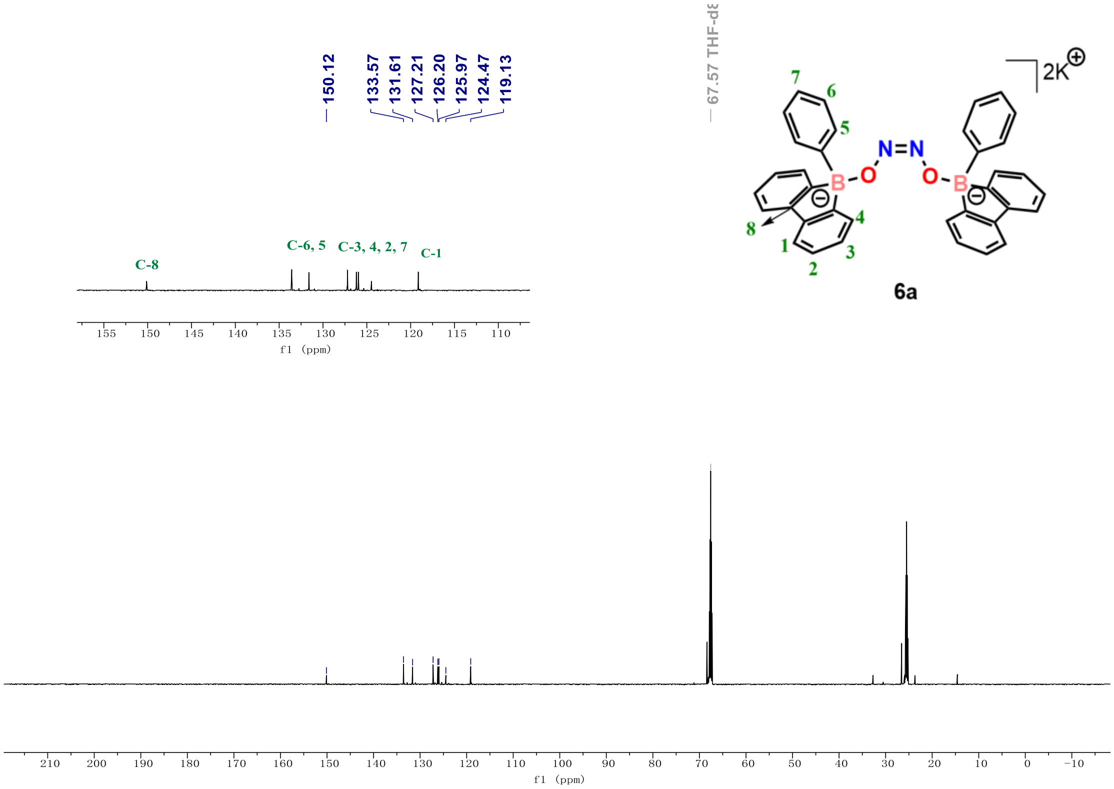

Figure S27. $^{13}C\{^{1}H\}$ NMR spectrum of **6a** (^{13}C: 151 MHz, THF-d_8)

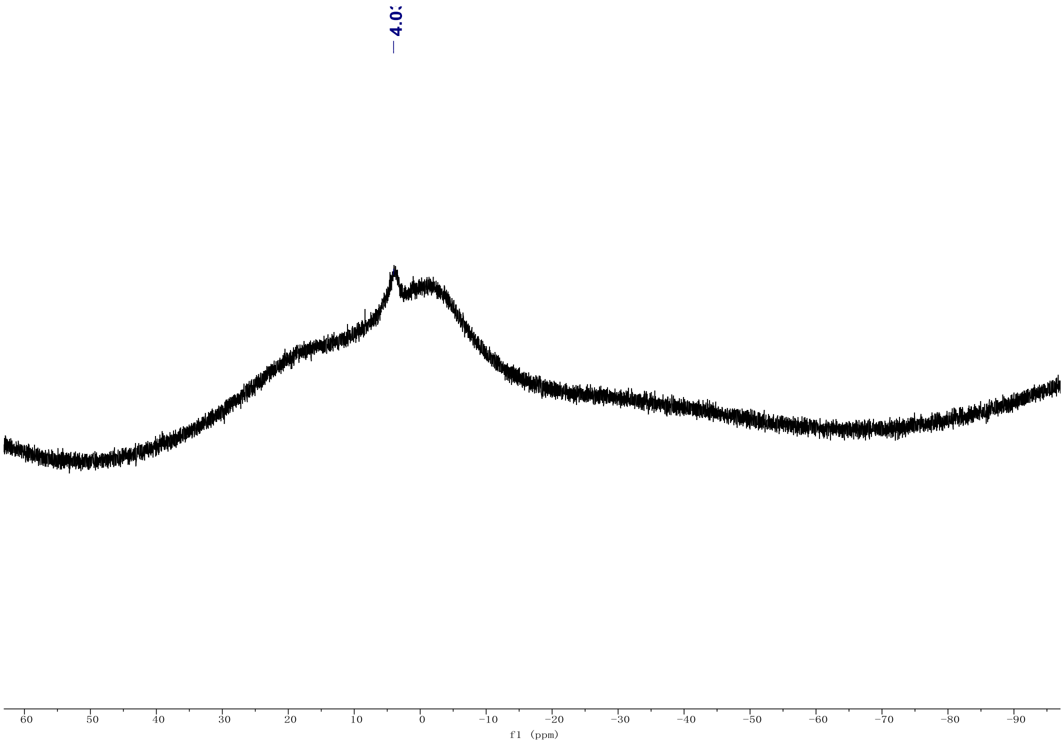

Figure S28. ^{11}B NMR spectrum of **6a** (^{11}B: 193 MHZ, THF-d_8)

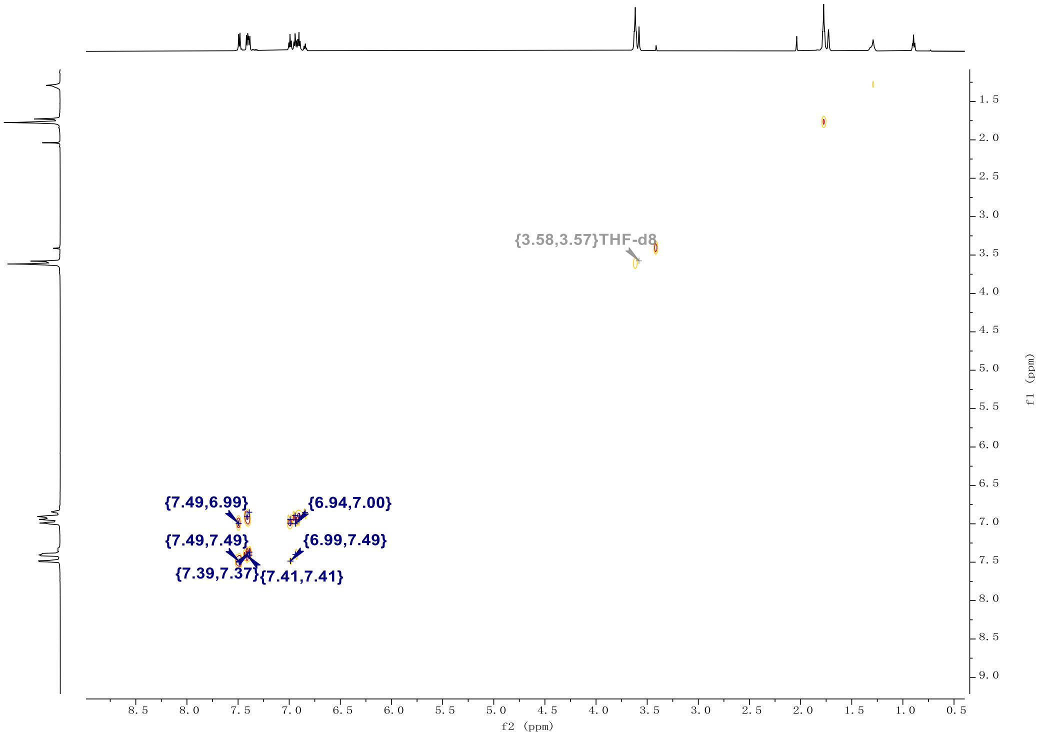

Figure S29. ^{1}H-^{1}H COSY NMR spectrum of **6a** (^{1}H: 600 MHZ, THF-d_8)

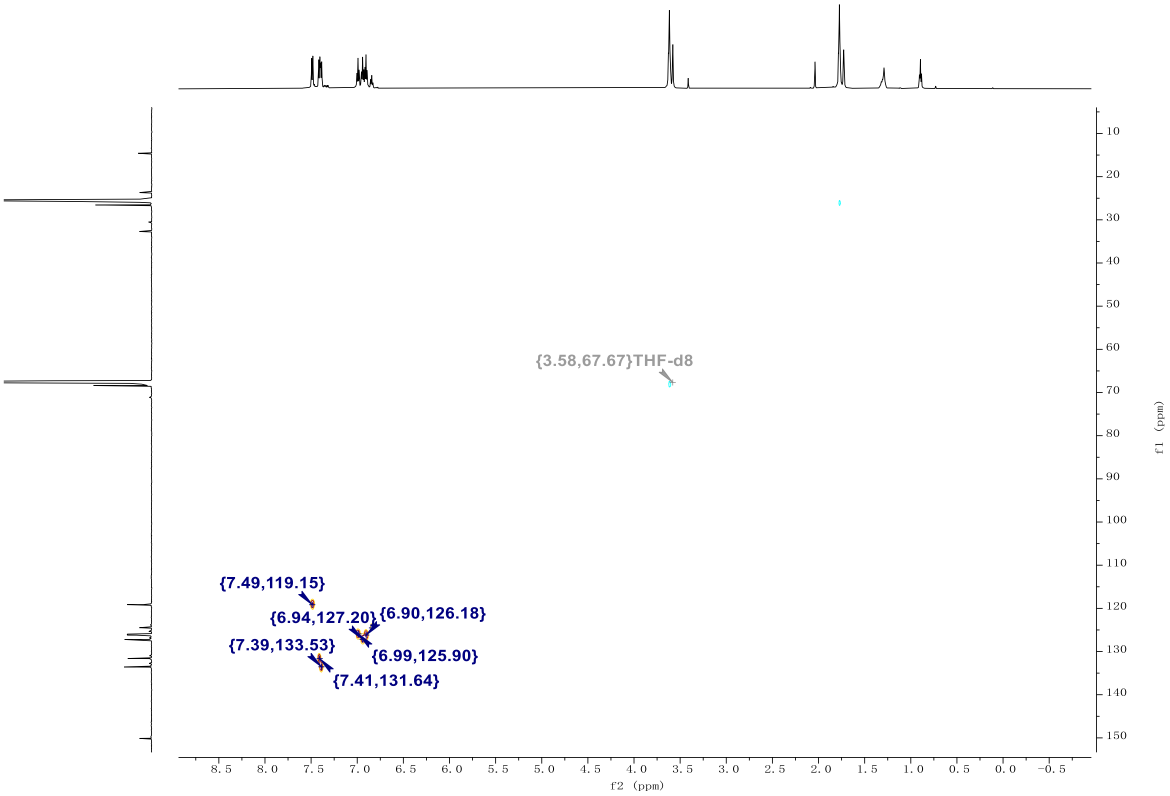

Figure S30. ^{1}H-^{13}C HSQC NMR spectrum of **6a** (^{1}H: 600 MHz and ^{13}C: 151 MHz, THF-d_8)

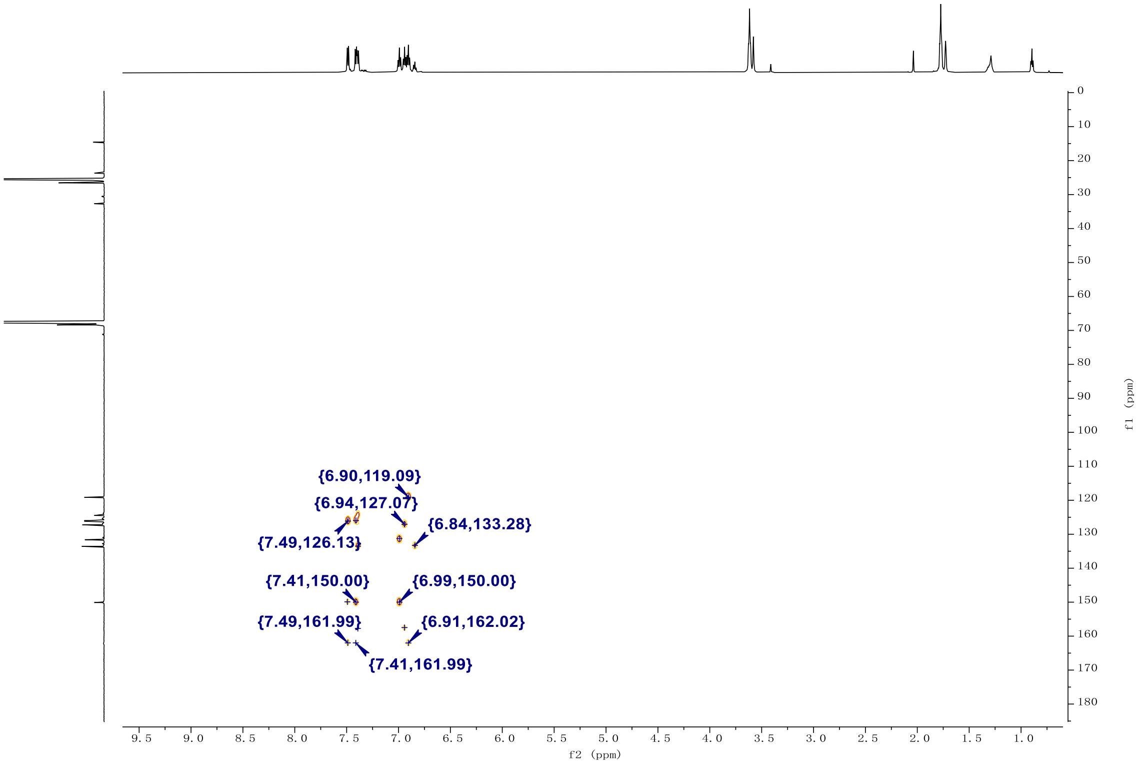

Figure S31. ^{1}H-^{13}C HMBC NMR spectrum of **6a** (^{1}H: 600 MHz and ^{13}C: 151 MHz, THF-d_8)

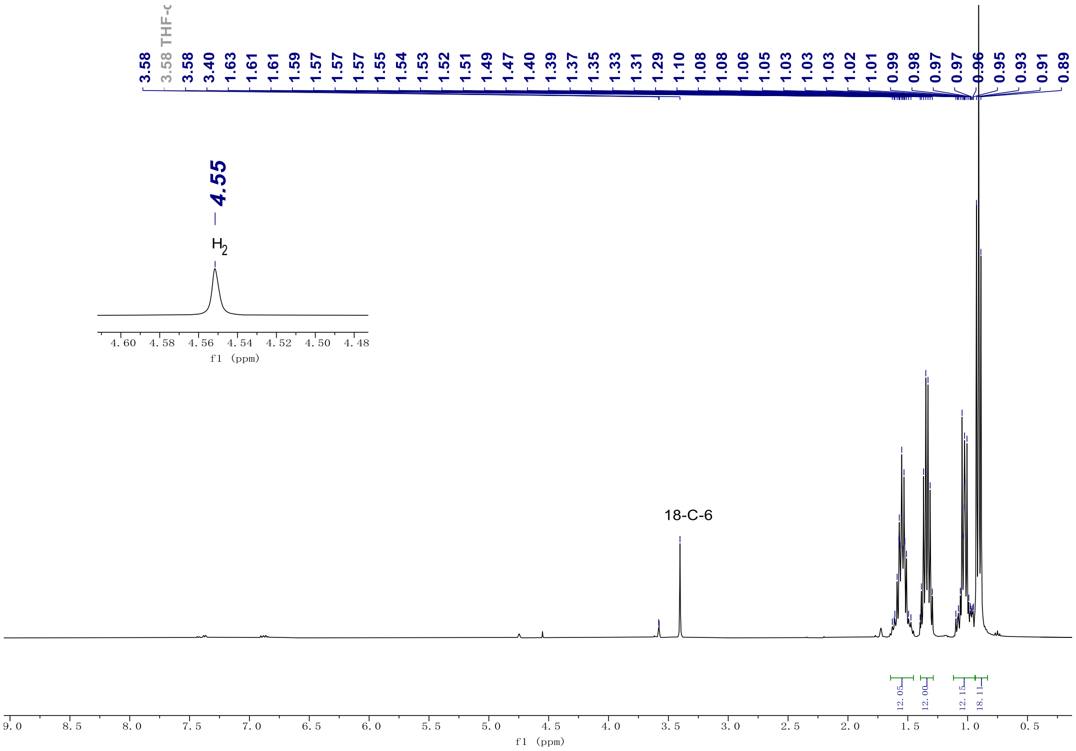

Figure S32. In Situ ^{1}H NMR spectrum of reaction of **2** and *n*Bu_3SnH (^{1}H: 400 MHz, THF-d_8)

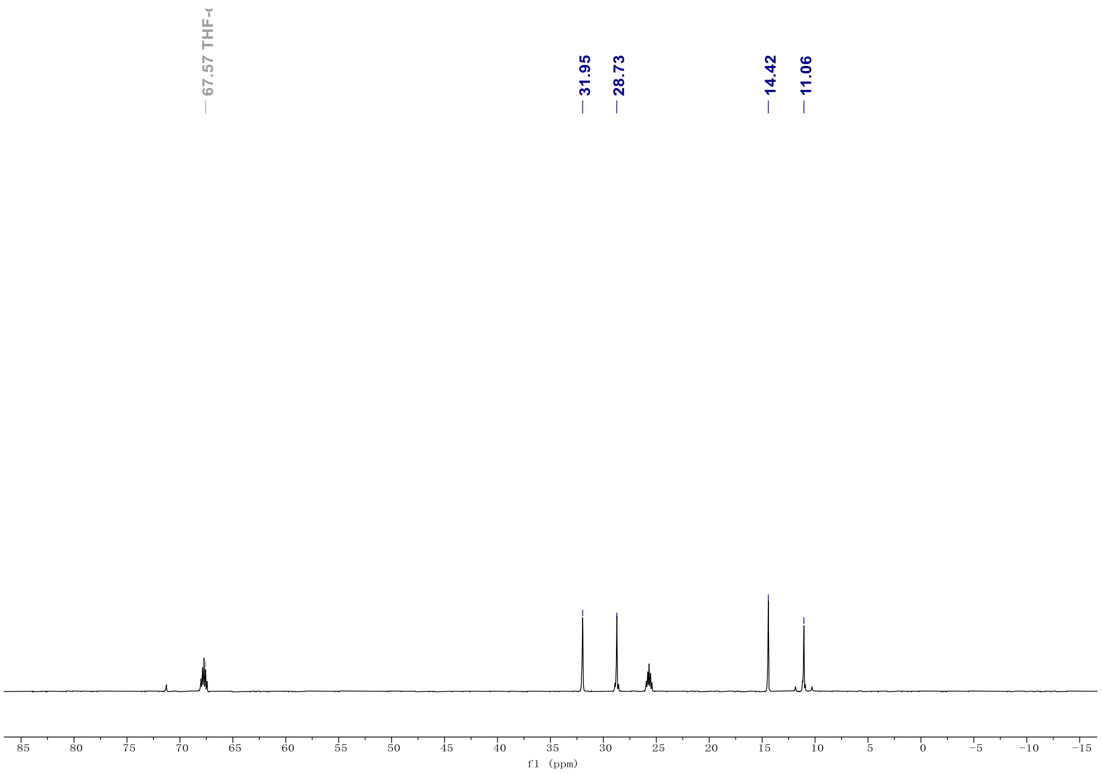

Figure S33. In Situ $^{13}C\{^{1}H\}$ NMR spectrum of reaction of **2** and *n*Bu_3SnH (^{13}C: 151 MHz, THF-d_8)

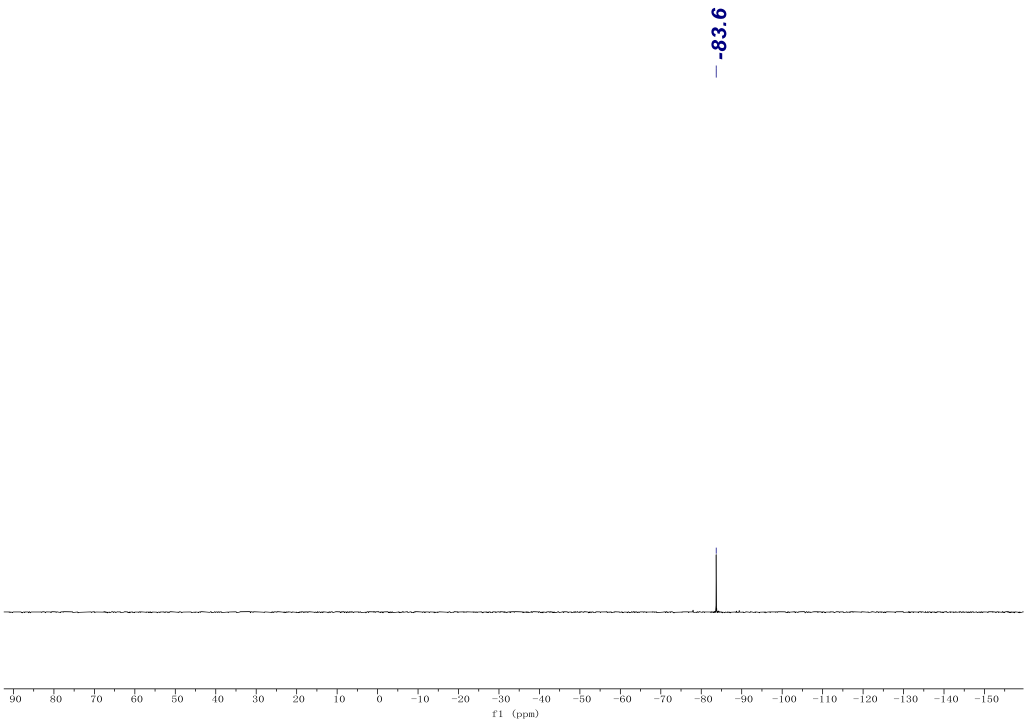

Figure S34. In Situ $^{119}Sn\{^{1}H\}$ NMR spectrum of reaction of **2** and *n*Bu_3SnH (^{119}Sn: 149 MHz, THF- d_8)

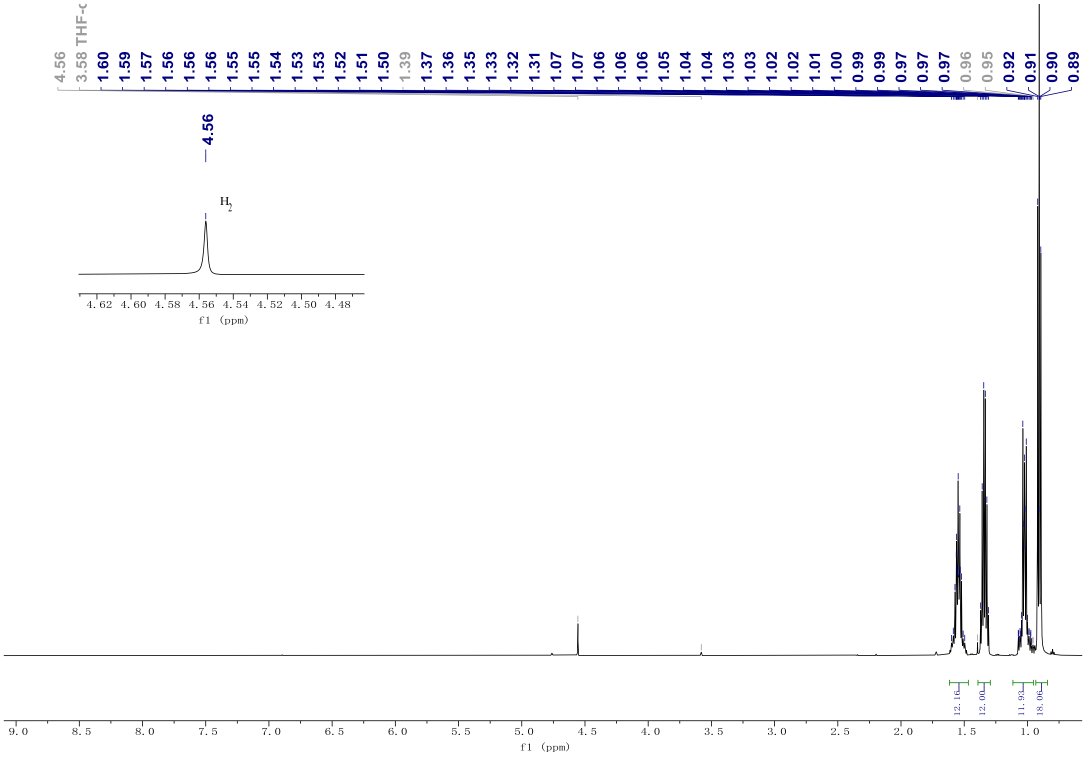

Figure S35. In Situ ^{1}H NMR spectrum of reaction of **3** and *n*Bu_3SnH (^{1}H: 400 MHz, THF-d_8)

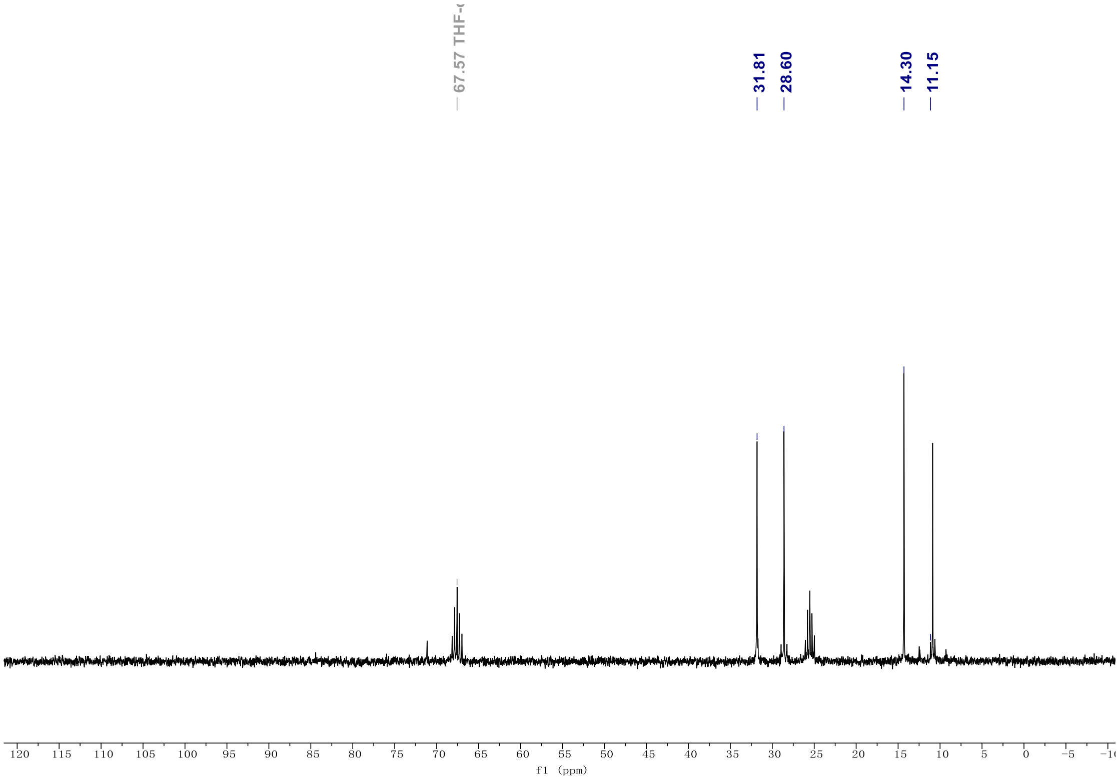

Figure S36. In Situ $^{13}C\{^{1}H\}$ NMR spectrum of reaction of **3** and *n*Bu_3SnH (^{13}C: 151 MHz, THF-d_8)

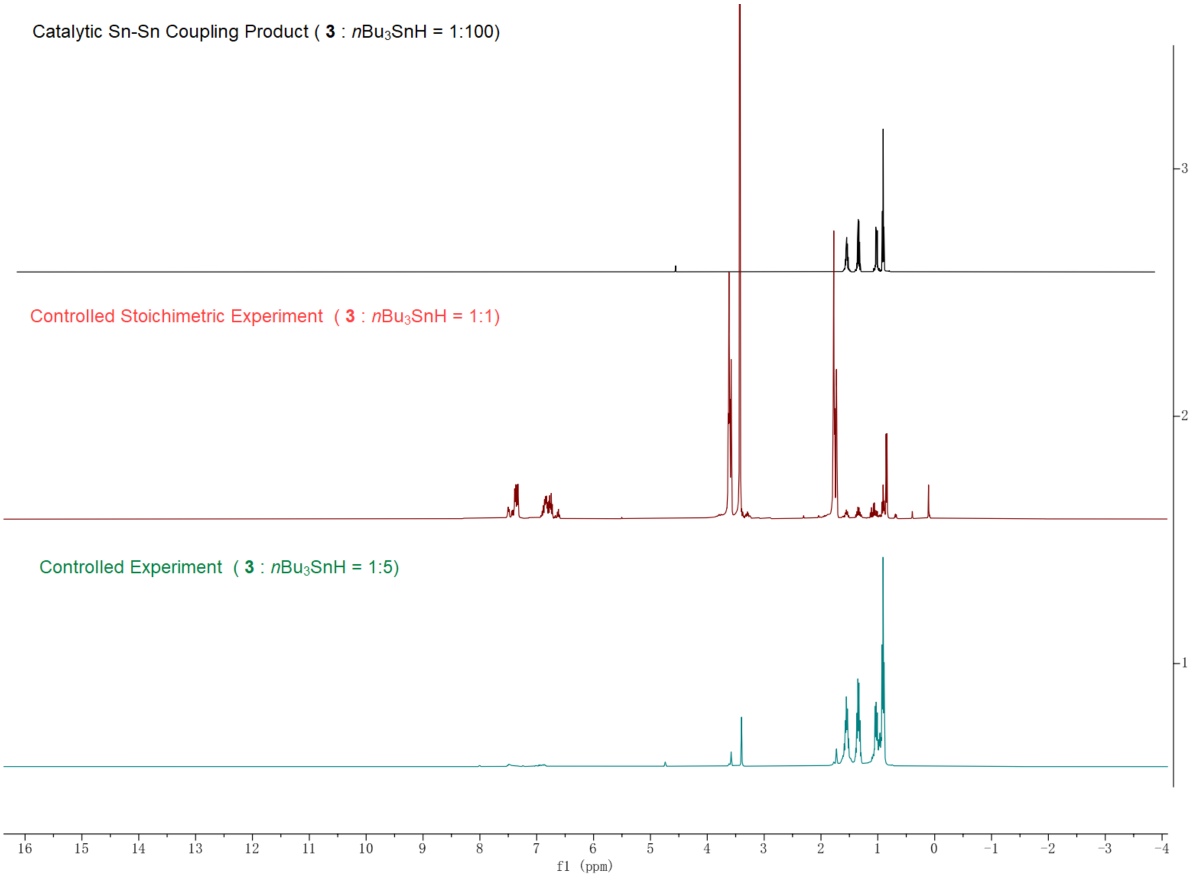

Figure S37. Controlled experiments between **3** and *n*Bu_3SnH (^{1}H: 400 MHZ, THF-d_8)

GC-MS, EPR and IR Study:

GC-MS Study

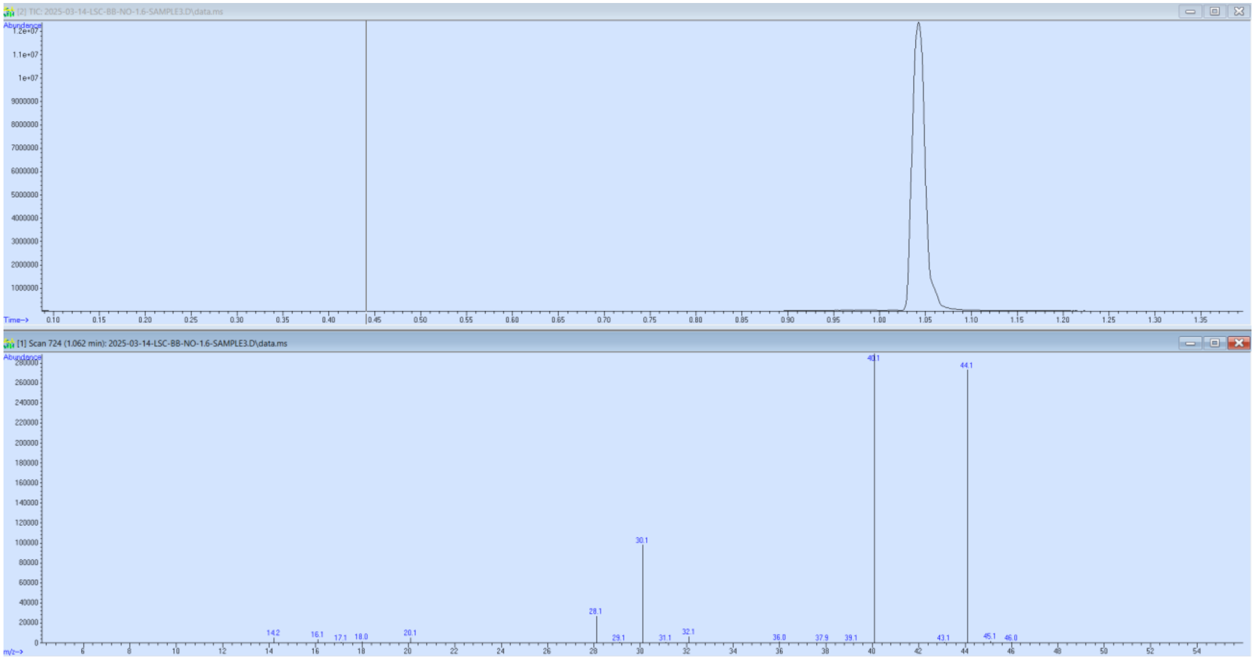

Figure S38. GC-MS spectrum for the reaction between **2** and 2 eq. NO.

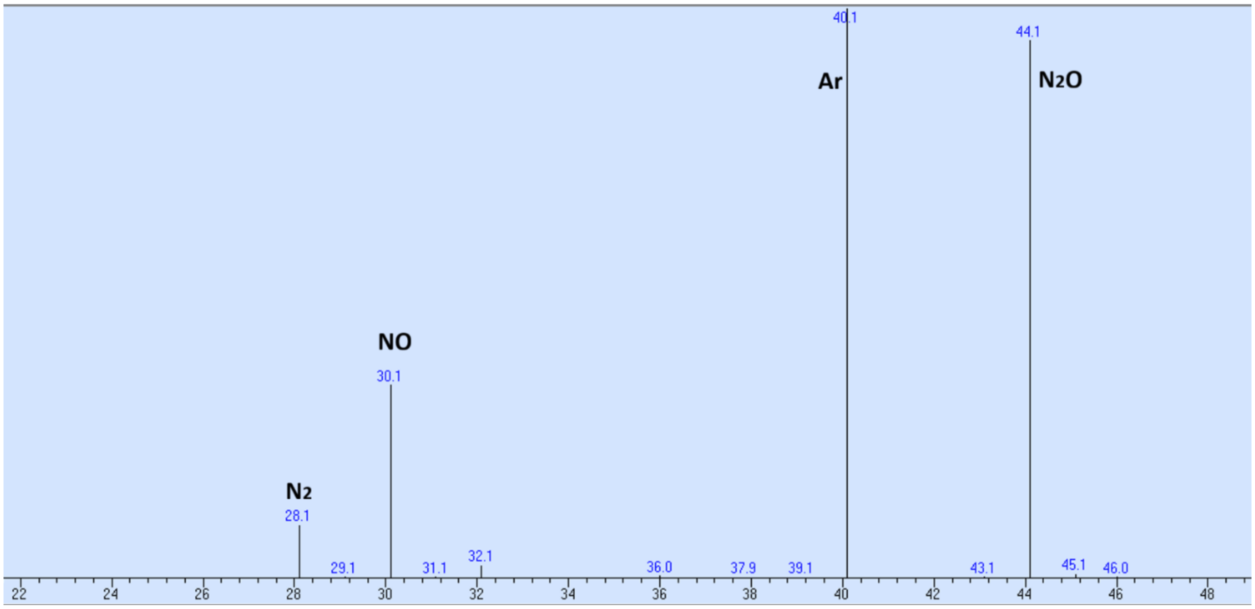

Figure S39. GC-MS spectrum for the reaction between **2** and 2 eq. NO (detailed).

EPR Study

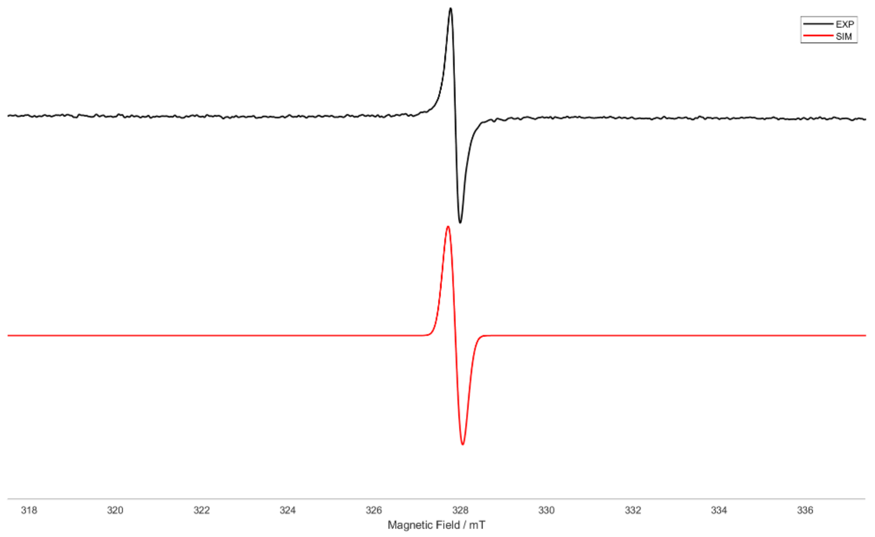

Figure S40. EPR spectra of Compound **2·(18-C-6)** (black line-- experiment spectrum (g_{iso} = 2.000), red line-- simulated spectrum. lw = 0.11 mT and g = 1.99902)

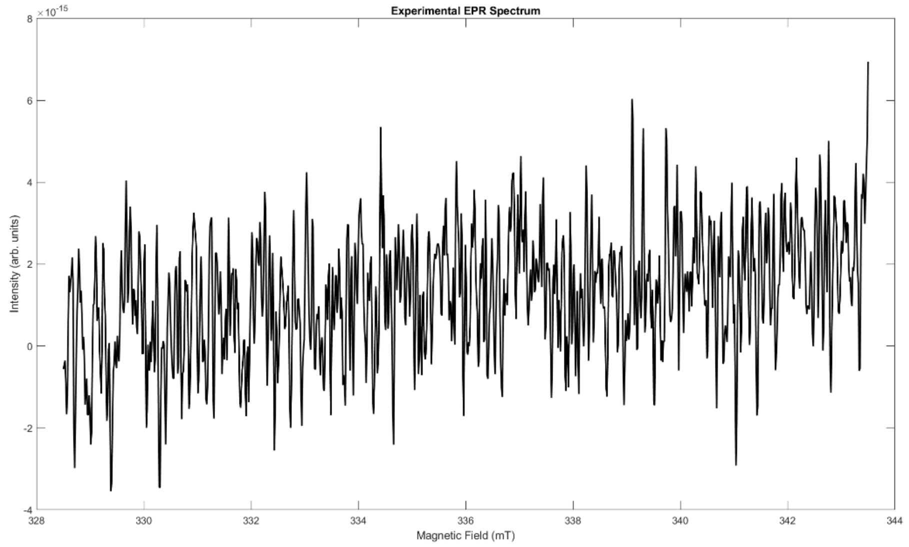

Figure S41. EPR spectrum of Compound **3·(18-C-6)**.

IR Study

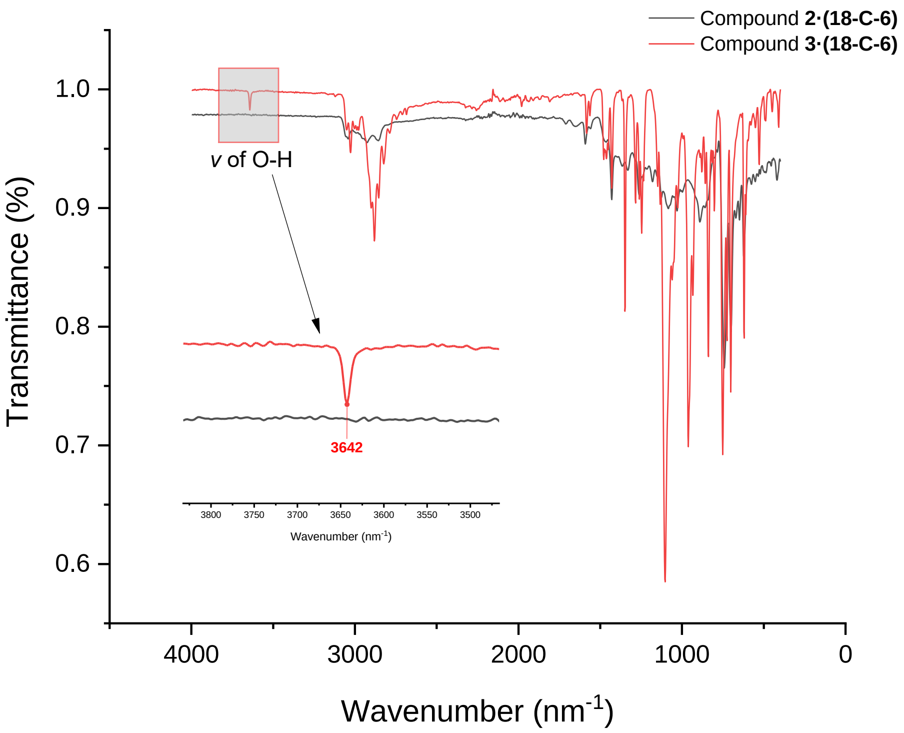

Figure S42. IR spectra of Compounds **2·(18-C-6)** and **3·(18-C-6).**

X-Ray Single-Crystal Diffraction

Single crystals of **2·(18-Crown-6)**, **3·(18-Crown-6)**, **4**, **5·(18-Crown-6)₂**, **6a**, **6b** and **7** were mounted on a Hampton loop using polybutene and diffracted on a Bruker D8 Venture diffractometer with a PHOTON 100 CMOS detector. Diffraction data were collected at 213 K, 223 K, or 233 K using either Cu-Kα radiation (λ = 1.54178 Å) or Mo-Kα radiation (λ = 0.71073 Å), using multi-scan (φ and ω scans). Data integration was performed using SAINT [4], and absorption corrections were applied using SADABS [5] with either numerical or multi-scan methods. Structures were solved with SHELXT [6] and refined using SHE5LXL [7] within the OLEX2 [8] graphical user interface program. All non-hydrogen atoms were refined anisotropically, while hydrogen atoms were placed in idealized positions and refined using the riding model, except where specifically addressed. For compound **5•(18-Crown-6)₂**, SIMU and AFIX 66 restraints were applied to model the highly disordered molecular skeleton. In compound **6a**, all the coordinated THF molecules were highly disordered, and SIMU and DFIX restraints were applied to model the coordinated THF. For compound **6b**, SIMU was used to model the disordered CH_3CN coordinated with K. For compound **7**, SIMU was also used to account for the disordered THF. In compound **4** the highly disordered toluene solvent was masked using the SQUEEZE function in the Platon[9] package. The hydrogen in O-H in compound **3·(18-Crown-6)** was located from the Fourier electron density map.

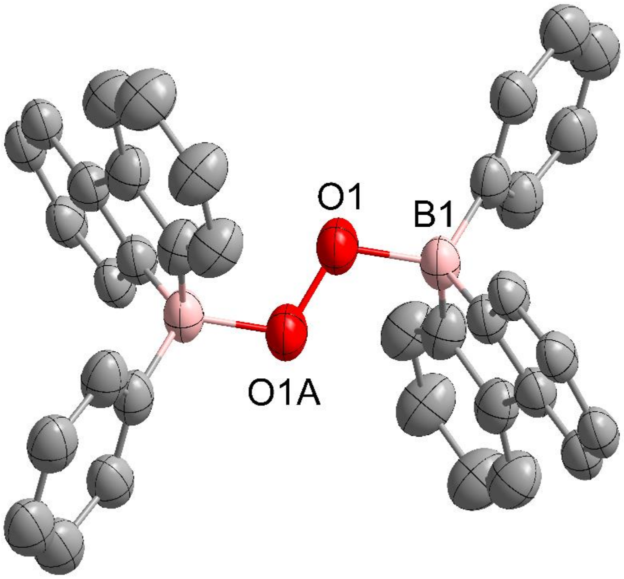

Figure S43. Molecular structures of compound **5**; key bond length and angles: O1-O1A, 1.468(6)Å, B1-O1, 1.595(18) Å, B1-O1-O1A 107.8(6). (thermal ellipsoids are set at the 30% robability level, hydrogen atoms and K cations are omitted for clarity)

**Table S1.** Crystal data and structure refinement for **2·(18-Crown-6)**.	
Identification code	2496522
Empirical formula	$C_{30}H_{37}BKO_7$
Formula weight	559.5
Temperature/K	213
Crystal system	orthorhombic
Space group	Pnma
a/Å	18.9174(6)
b/Å	14.5562(5)
c/Å	10.7345(3)
α/°	90
β/°	90
γ/°	90
Volume/Å^3	2955.91(16)
Z	4
ρ_{calc}g/cm^3	1.257
μ/mm^{-1}	0.224
F(000)	1188
Crystal size/mm^3	0.29 × 0.28 × 0.08
Radiation	MoKα (λ = 0.71073)
2Θ range for data collection/°	4.714 to 55.024
Index ranges	-24 ≤ h ≤ 24, -18 ≤ k ≤ 18, -13 ≤ l ≤ 12
Reflections collected	37729
Independent reflections	3522 [R_{int} = 0.0751, R_{sigma} = 0.0359]
Data/restraints/parameters	3522/0/193
Goodness-of-fit on F^2	1.098
Final R indexes [I>=2σ (I)]	R_1 = 0.0507, wR_2 = 0.1156
Final R indexes [all data]	R_1 = 0.0691, wR_2 = 0.1266
Largest diff. peak/hole / e Å^{-3}	0.43/-0.35

**Table S2.** Crystal data and structure refinement for **3·(18-Crown-6)**	
Identification code	2496523
Empirical formula	$C_{30}H_{38}BKO_7$
Formula weight	560.51
Temperature/K	213
Crystal system	orthorhombic
Space group	$P2_12_12_1$
a/Å	10.7077(5)
b/Å	14.6098(7)
c/Å	18.9554(10)
α/°	90
β/°	90
γ/°	90
Volume/Å^3	2965.3(3)
Z	4
ρ_{calc}g/cm^3	1.256
μ/mm^{-1}	0.223
F(000)	1192
Crystal size/mm^3	0.26 × 0.19 × 0.06
Radiation	MoKα (λ = 0.71073)
2Θ range for data collection/°	4.298 to 56.712
Index ranges	-14 ≤ h ≤ 14, -16 ≤ k ≤ 19, -25 ≤ l ≤ 25
Reflections collected	26580
Independent reflections	7364 [R_{int} = 0.0642, R_{sigma} = 0.0607]
Data/restraints/parameters	7364/0/357
Goodness-of-fit on F^2	1.019
Final R indexes [I>=2σ (I)]	R_1 = 0.0440, wR_2 = 0.0988
Final R indexes [all data]	R_1 = 0.0640, wR_2 = 0.1104
Largest diff. peak/hole / e Å^{-3}	0.19/-0.26

**Table S3.** Crystal data and structure refinement for **4**	
Identification code	2496524
Empirical formula	$C_{21.5}H_{18}BK$
Formula weight	326.27
Temperature/K	213
Crystal system	trigonal
Space group	R-3
a/Å	25.1013(6)
b/Å	25.1013(6)
c/Å	14.6147(5)
α/°	90
β/°	90
γ/°	120
Volume/Å^3	7974.7(5)
Z	18
ρ_{calc}g/cm^3	1.223
μ/mm^{-1}	2.57
F(000)	3078
Crystal size/mm^3	0.22 × 0.04 × 0.04
Radiation	CuKα (λ = 1.54178)
2Θ range for data collection/°	7.042 to 137.114
Index ranges	-26 ≤ h ≤ 30, -30 ≤ k ≤ 29, -17 ≤ l ≤ 13
Reflections collected	13146
Independent reflections	3241 [R_{int} = 0.0865, R_{sigma} = 0.0664]
Data/restraints/parameters	3241/0/185
Goodness-of-fit on F^2	1.078
Final R indexes [I>=2σ (I)]	R_1 = 0.0649, wR_2 = 0.1931
Final R indexes [all data]	R_1 = 0.0974, wR_2 = 0.2210
Largest diff. peak/hole / e Å^{-3}	0.16/-0.38

**Table S4.** Crystal data and structure refinement for **5·(18-Crown-6)$_2$**	
Identification code	2496525
Empirical formula	$C_{48}H_{50}B_2KO_8$
Formula weight	815.6
Temperature/K	213
Crystal system	monoclinic
Space group	C2/c
a/Å	15.7811(8)
b/Å	19.5898(10)
c/Å	15.4975(8)
α/°	90
β/°	112.526(3)
γ/°	90
Volume/Å^3	4425.5(4)
Z	4
ρ_{calc}g/cm^3	1.224
μ/mm^{-1}	1.47
F(000)	1724
Crystal size/mm^3	0.19 × 0.14 × 0.08
Radiation	CuKα (λ = 1.54178)
2Θ range for data collection/°	7.56 to 136.71
Index ranges	$-19 \le h \le 18$, $-23 \le k \le 23$, $-18 \le l \le 15$
Reflections collected	13003
Independent reflections	4048 [R_{int} = 0.0666, R_{sigma} = 0.0643]
Data/restraints/parameters	4048/564/391
Goodness-of-fit on F^2	1.032
Final R indexes [I>=2σ (I)]	R_1 = 0.0663, wR_2 = 0.1854
Final R indexes [all data]	R_1 = 0.1019, wR_2 = 0.2168
Largest diff. peak/hole / e Å^{-3}	0.26/-0.19

**Table S5.** Crystal data and structure refinement for **6a**	
Identification code	2496527
Empirical formula	$C_{57}H_{68}B_2K_2N_2O_7$
Formula weight	992.95
Temperature/K	223
Crystal system	orthorhombic
Space group	Pnma
a/Å	21.4853(16)
b/Å	16.1068(10)
c/Å	15.2474(11)
α/°	90
β/°	90
γ/°	90
Volume/Å^3	5276.5(6)
Z	4
ρ_{calc}g/cm^3	1.25
μ/mm^{-1}	0.233
F(000)	2112
Crystal size/mm^3	0.09 × 0.08 × 0.04
Radiation	MoKα (λ = 0.71073)
2Θ range for data collection/°	4.638 to 52.888
Index ranges	-26 ≤ h ≤ 26, -18 ≤ k ≤ 20, -19 ≤ l ≤ 19
Reflections collected	55080
Independent reflections	5591 [R_{int} = 0.0920, R_{sigma} = 0.0452]
Data/restraints/parameters	5591/288/456
Goodness-of-fit on F^2	1.028
Final R indexes [I>=2σ (I)]	R_1 = 0.0915, wR_2 = 0.2487
Final R indexes [all data]	R_1 = 0.1391, wR_2 = 0.2987
Largest diff. peak/hole / e Å^{-3}	0.86/-0.70

**Table S6.** Crystal data and structure refinement for **6b**	
Identification code	2496526
Empirical formula	$C_{48}H_{44}B_2K_2N_8O_2$
Formula weight	864.73
Temperature/K	233
Crystal system	orthorhombic
Space group	Pbca
a/Å	10.899(9)
b/Å	20.16(2)
c/Å	22.374(14)
α/°	90
β/°	90
γ/°	90
Volume/Å^3	4915(8)
Z	4
ρ_{calc}g/cm^3	1.169
μ/mm^{-1}	0.237
F(000)	1808
Crystal size/mm^3	0.19 × 0.13 × 0.06
Radiation	MoKα (λ = 0.71073)
2Θ range for data collection/°	4.432 to 54.886
Index ranges	-14 ≤ h ≤ 14, -20 ≤ k ≤ 26, -27 ≤ l ≤ 29
Reflections collected	44291
Independent reflections	5499 [R_{int} = 0.0816, R_{sigma} = 0.0486]
Data/restraints/parameters	5499/66/312
Goodness-of-fit on F^2	1.088
Final R indexes [I>=2σ (I)]	R_1 = 0.0664, wR_2 = 0.1348
Final R indexes [all data]	R_1 = 0.1096, wR_2 = 0.1582
Largest diff. peak/hole / e Å^{-3}	0.29/-0.33

**Table S7.** Crystal data and structure refinement for **7**	
Identification code	2496528
Empirical formula	$C_{52}H_{58}B_2K_2N_2O_6$
Formula weight	906.82
Temperature/K	213
Crystal system	monoclinic
Space group	$P2_1/c$
a/Å	13.9938(6)
b/Å	10.6261(5)
c/Å	17.4836(8)
α/°	90
β/°	112.235(2)
γ/°	90
Volume/Å^3	2406.48(19)
Z	2
ρ_{calc}g/cm^3	1.251
μ/mm^{-1}	2.141
F(000)	960
Crystal size/mm^3	0.25 × 0.25 × 0.1
Radiation	CuKα (λ = 1.54178)
2Θ range for data collection/°	10.472 to 137.05
Index ranges	$-16 \leq h \leq 16$, $-9 \leq k \leq 12$, $-21 \leq l \leq 21$
Reflections collected	16363
Independent reflections	4354 [R_{int} = 0.0854, R_{sigma} = 0.0768]
Data/restraints/parameters	4354/264/372
Goodness-of-fit on F^2	1.063
Final R indexes [I>=2σ (I)]	R_1 = 0.0536, wR_2 = 0.1403
Final R indexes [all data]	R_1 = 0.0647, wR_2 = 0.1506
Largest diff. peak/hole / e Å^{-3}	0.30/-0.62

Computation details

Quantum chemical calculations were all performed at the density functional theory (DFT) level using the hybrid meta-GGA M06-2X functional [10], which has been proven to give reliable results to the reaction energy barriers involving organic molecules and their geometries. The 6-31+G(d,p) basis set was employed for geometry optimization and harmonic vibrational frequency analysis. Single-point energies were performed with the 6-311++G(d,p) basis set [11, 12] to provide more accurate energetic results. All calculations were performed in the tetrahydrogen furan solution which was modelled by the polarizable continuum solvation model (IEFPCM) [13] with radii and non-electrostatic terms for Truhlar and coworkers' SMD solvation model [14]. This solvation model is by far the most reliable one in predicting solvation free energies. The convergence criteria used for geometry optimization were 4.50×10^{-4} au. for gradients, and 1.80×10^{-3} au. for displacements. Harmonic vibrational analyses were carried out to confirm if the optimized structure is a local minimum structure or a first order transition state and to provide zero-point vibrational energy corrections and thermal corrections to various thermodynamic properties. Transition states were further confirmed by IRC calculations [15, 16.] All the calculations were performed by using the Gaussian 16 software package [17.]

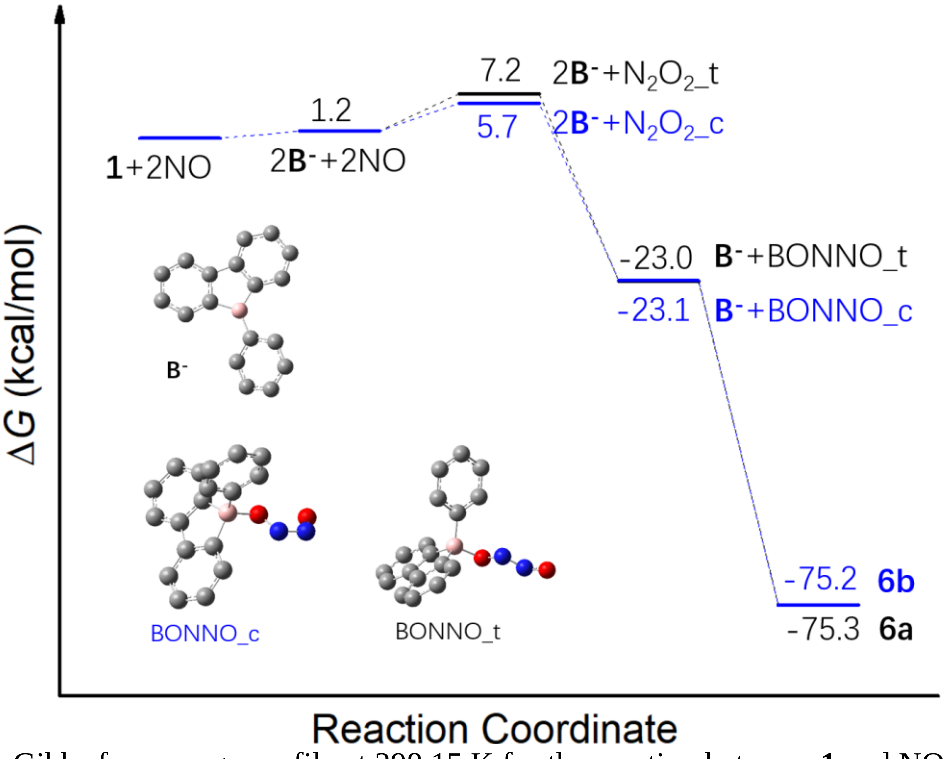

Figure S44. Gibbs free energy profile at 298.15 K for the reaction between **1** and NO to produce **6a** and **6b**.

1

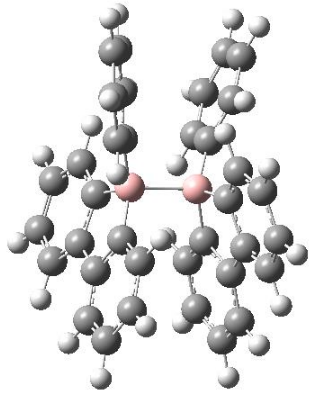

-2 1

C, 0, -2.4645460248, -1.0936559296, 0.8368141414

C, 0, -1.3626639173, -1.9146808991, 1.360916542

C, 0, 1.0568209372, -2.0350611708, -1.4706312212

C, 0, 2.238827245, -1.4228966477, -0.8431998225

C, 0, 0.9508428584, -1.7957019677, 1.9967956586

H, 0, 1.8943501487, -1.2600875488, 2.1025084749

C, 0, -1.3956558923, -3.2791408286, 1.658985753

H, 0, -2.3061479761, -3.8581721448, 1.5111373808

C, 0, -0.2487723405, 2.9462799408, 1.3009891526

H, 0, -0.9072606297, 3.092466886, 0.447687554

C, 0, -2.9979421138, 1.1446952605, 0.1481628904

H, 0, -2.7364089966, 2.184301832, -0.0387212337

C, 0, 0.9240122757, -3.354662664, -1.9077777551

H, 0, 1.7525445426, -4.0556860597, -1.8163138167

C, 0, -1.2089452004, -1.5588547417, -2.1113085038

H, 0, -2.0775658611, -0.9045193421, -2.1701626767

C, 0, 0.9211263568, -3.1557108651, 2.3198592327

H, 0, 1.8204529558, -3.6410899788, 2.6937401234

C, 0, -3.773818995, -1.5050169431, 0.5789649571

H, 0, -4.0740094847, -2.5376907434, 0.7507502075

C, 0, 3.0073299371, 0.6232469109, 0.1563675462

H, 0, 2.8559299279, 1.6526972065, 0.4782160238

C, 0, 3.4525021456, -2.0461950036, -0.5433277384

H, 0, 3.618043764, -3.0913324521, -0.8014372372

C, 0, 1.0683690086, 2.7272250092, -1.6821627931

H, 0, 2.0228768768, 2.6210634829, -1.1744087925

C, 0, 0.2459572578, 4.0762753765, 1.9520852151

H, 0, -0.0509978241, 5.0664438333, 1.6117952609

C, 0, 4.2255716978, 0.0071958822, 0.460722575

H, 0, 5.0013156373, 0.5681710434, 0.9777207871

C, 0, -0.247268186, -3.9034093145, 2.1454879524

H, 0, -0.2592138856, -4.9641181564, 2.3832623808

C, 0, 0.9780874161, 1.5390885875, 2.7786213612

H, 0, 1.2747631186, 0.5551982637, 3.1337084854

C, 0, -4.3154830001, 0.7447621392, -0.0999119575

H, 0, -5.0425436544, 1.4719088499, -0.4559040439

C, 0, 4.4525534857, -1.3275543682, 0.1108859707

H, 0, 5.399264271, -1.8037770531, 0.3533008784

C, 0, -0.2856170073, -3.7781091937, -2.4588753859

H, 0, -0.4032670685, -4.8039465403, -2.7989466534

C, 0, -4.7052037079, -0.5824132468, 0.103792493

H, 0, -5.7278555788, -0.8907594289, -0.0980341896

C, 0, -1.0484587607, 1.8824267702, -2.3614541552

H, 0, -1.8006542812, 1.0994394893, -2.3895201137

C, 0, -1.3475686852, -2.8758046345, -2.5618094071

H, 0, -2.2952739985, -3.2046140068, -2.9834350515

C, 0, 1.1268136109, 3.9433643827, 3.0267249773

H, 0, 1.5203614202, 4.8208815268, 3.5330137838

C, 0, 1.4868290747, 2.6604211086, 3.4378260442

H, 0, 2.1660708201, 2.5299139401, 4.2780715956

C, 0, 0.8042450598, 3.9345138261, -2.3304500042

H, 0, 1.5483999617, 4.728502448, -2.31336785

C, 0, -1.3296569467, 3.0834443728, -3.0134800451

H, 0, -2.2759812178, 3.202276572, -3.5376805544

C, 0, -0.4047547421, 4.1281953997, -2.9981920394

H, 0, -0.6180282148, 5.0673390539, -3.5017944855

C, 0, 0.1006225587, 1.6292265498, 1.6765885814

C, 0, -0.1848123672, -1.131519251, 1.502800244

C, 0, -2.024163672, 0.2393829853, 0.6022128527

C, 0, -0.0083740694, -1.0962033148, -1.5479905675

C, 0, 1.9745860236, -0.0689485936, -0.5003430396

C, 0, 0.1508685086, 1.6538623604, -1.6510166589

B, 0, 0.4420981464, 0.2877334812, -0.8529262728

B, 0, -0.4408072086, 0.338710862, 0.8901135985

B^-

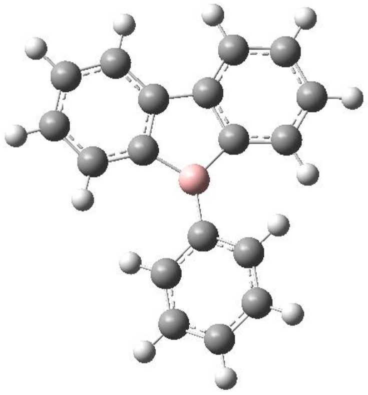

-1 2

C, 0, -0.7318767302, 0.0247361675, -1.9547371371

C, 0, -1.5954946959, 0.074024752, -3.0496445639

H, 0, -1.2017991855, 0.0667958031, -4.0648356056

C, 0, -2.6224789169, 0.1139251306, -0.4369589847

H, 0, -3.0467930032, 0.1497660277, 0.5655610988

C, 0, -2.9715510315, 0.1389363518, -2.8402392827

H, 0, -3.6501662148, 0.1774363625, -3.6879911208

C, 0, -3.4808899404, 0.1636909845, -1.5322268868

H, 0, -4.5552363639, 0.226060897, -1.375979758

C, 0, 0., 0., 4.7735083959

H, 0, 0., 0., 5.859846861

C, 0, 1.0598957069, 0.5582989581, 2.6731208348

H, 0, 1.891968462, 1.0195369178, 2.1447468546

C, 0, 1.0634787771, 0.5620801901, 4.0655890324

H, 0, 1.8961909595, 1.0102300636, 4.6023795501

C, 0, -1.0598957069, -0.5582989581, 2.6731208348

H, 0, -1.891968462, -1.0195369178, 2.1447468546

C, 0, -1.0634787771, -0.5620801901, 4.0655890324

H, 0, -1.8961909595, -1.0102300636, 4.6023795501

C, 0, -1.2253806299, 0.0326686513, -0.6073717449

C, 0, 1.2253806299, -0.0326686513, -0.6073717449

C, 0, 0., 0., 1.9230501208

B, 0, 0., 0., 0.3584474904

N_2O_2_c

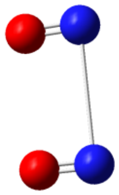

0 1

N, 0, 0., 1.47558498, 0.61117425

N, 0, 0., -1.47558498, 0.61117425

O, 0, 0., 1.44370115, -0.53503975

O, 0, 0., -1.44370115, -0.53503975

N_2O_2_t

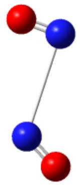

0 1

N, 0, 0.5339750071, 1.5246431794, -0.0004815313

N, 0, -0.3899721288, -1.3044958519, 0.000669713

O, 0, -0.5392345146, 1.9263635043, 0.0001689385

O, 0, 0.4043316363, -2.1322108317, 0.0002128798

BONNO_c

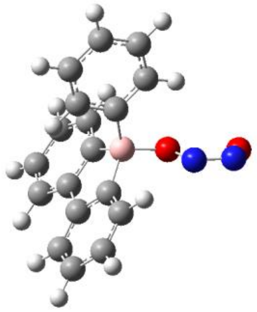

-1 2

C, 0, -0.2258746544, -1.9245213754, 0.6635696976

C, 0, -0.7547553506, -2.891290768, 1.5174783175

H, 0, -1.2799948747, -3.7584849404, 1.1231429794

C, 0, 0.5853990489, -0.6518267172, 2.5423628842

H, 0, 1.0971663946, 0.2169106566, 2.9532610053

C, 0, -0.6044994353, -2.7330141696, 2.8966515785

H, 0, -1.0124465808, -3.4791310104, 3.5733233891

C, 0, 0.0617615662, -1.6191666007, 3.4087939443

H, 0, 0.1710609174, -1.5039481143, 4.4840923989

C, 0, -0.2813582158, -1.8954532283, -0.8188912682

C, 0, -0.8624519829, -2.8307371889, -1.6735618707

H, 0, -1.3525368753, -3.7192936631, -1.281388575

C, 0, 0.3950114294, -0.5418095358, -2.6954212887

H, 0, 0.8723782672, 0.3474681072, -3.1060268334

C, 0, -0.8047264392, -2.6159908703, -3.0529072701

H, 0, -1.2521789127, -3.3378789813, -3.7308762638

C, 0, -0.1783222113, -1.4788169737, -3.5635496011

H, 0, -0.1389605469, -1.321967882, -4.63836291

C, 0, -0.8286393868, 4.2644499944, 0.0082666064

H, 0, -1.2643033923, 5.2593797574, 0.0306952784

C, 0, -1.0598542547, 1.8669125058, 0.189450595

H, 0, -1.6979169949, 0.9989452017, 0.3569734097

C, 0, -1.6278046994, 3.1384018907, 0.2151462292

H, 0, -2.6927065896, 3.2535826607, 0.4011567924

C, 0, 1.0917225331, 2.8159833846, -0.246979055

H, 0, 2.1603643829, 2.7193111451, -0.4295718257

C, 0, 0.5338563921, 4.097415622, -0.2235717945

H, 0, 1.1679837512, 4.9653801676, -0.3860497311

C, 0, 0.4549398039, -0.791791109, 1.1619259068

C, 0, 0.3545469649, -0.7382433443, -1.3169962975

C, 0, 0.3130728466, 1.6680838915, -0.0390770093

B, 0, 0.8991869622, 0.1607776222, -0.0791712898

N, 0, 3.0656253541, 0.6002859101, 0.8339861858

O, 0, 2.445939627, 0.2354263283, -0.2298747376

N, 0, 4.4056110695, 0.7192871516, 0.6589023497

O, 0, 4.8516056966, 0.3780648551, -0.4379235067

BONNO_t

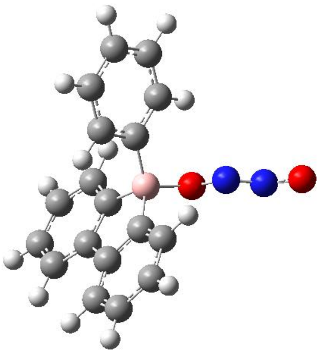

-1 2

C, 0, -0.2241303163, -1.9439523913, 0.6502309774

C, 0, -0.7538354746, -2.9287642227, 1.4829565087

H, 0, -1.2633874025, -3.7965884166, 1.0690024081

C, 0, 0.5461107905, -0.6907252956, 2.555745329

H, 0, 1.0385621779, 0.1792956778, 2.9892486394

C, 0, -0.6253514593, -2.7892654289, 2.866857415

H, 0, -1.0341819847, -3.5493865646, 3.5273387251

C, 0, 0.0222024926, -1.6757539022, 3.4026855443

H, 0, 0.1142930226, -1.5736182391, 4.4810873979

C, 0, -0.2602779377, -1.8956923521, -0.8318380285

C, 0, -0.8319832065, -2.8211755982, -1.7036089608

H, 0, -1.3238517197, -3.7153573961, -1.3261763491

C, 0, 0.4247908987, -0.5162393626, -2.6840905385

H, 0, 0.9028705903, 0.3793422456, -3.0805227196

C, 0, -0.7685765674, -2.5873451819, -3.0794120561

H, 0, -1.2104966409, -3.3009893469, -3.7697786561

C, 0, -0.1426957856, -1.440744889, -3.5696054096

H, 0, -0.0999199852, -1.2667764918, -4.6417847001

C, 0, -0.799520857, 4.2756729491, -0.0440319333

H, 0, -1.220744302, 5.2772748145, -0.0480190179

C, 0, -1.0016467559, 1.9130795414, 0.4243793386

H, 0, -1.6048330839, 1.0829536278, 0.7921603836

C, 0, -1.552683542, 3.1942413364, 0.4150690455

H, 0, -2.5673982473, 3.3521113224, 0.7724678746

C, 0, 1.0379109621, 2.7671383332, -0.4815618957

H, 0, 2.0576337717, 2.6173825543, -0.8336970726

C, 0, 0.5010785479, 4.0560993469, -0.4939268055

H, 0, 1.1006254082, 4.8902527736, -0.8506512555

C, 0, 0.4363788658, -0.808281574, 1.1705687697

C, 0, 0.380911085, -0.7307969829, -1.308006494

C, 0, 0.3062617214, 1.6598575534, -0.0220992918

B, 0, 0.9201568144, 0.1584420513, -0.0535178787

N, 0, 2.9957829099, 0.7526253099, 0.9239556626

O, 0, 2.4443528987, 0.208393843, -0.1798142125

N, 0, 4.2285191613, 0.7107233777, 0.9024886185

O, 0, 5.0455666096, 1.1180702185, 1.7247882177

TS2

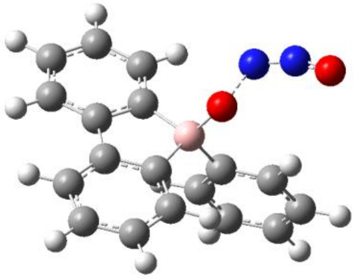

-1 2

C, 0, -2.1677532917, 0.6251004326, -0.38288978

C, 0, -3.240475783, 1.341189427, -0.9139251976

H, 0, -4.2471637299, 0.9282308994, -0.9070060325

C, 0, -0.6496939293, 2.4051328703, -0.9425901987

H, 0, 0.3526401309, 2.8318191269, -0.9716304865

C, 0, -3.0090743992, 2.6048626632, -1.4613233707

H, 0, -3.8357682467, 3.1734693111, -1.8788625184

C, 0, -1.7187790626, 3.1356057388, -1.4771125838

H, 0, -1.5457124715, 4.1185139231, -1.9083868832

C, 0, -2.1996120438, -0.7233287751, 0.2300211902

C, 0, -3.2984283091, -1.5684819136, 0.3823602335

H, 0, -4.2859903668, -1.2674088593, 0.038508311

C, 0, -0.7573408913, -2.3595164245, 1.2609524789

H, 0, 0.2262890421, -2.6813347106, 1.6017176554

C, 0, -3.1199698322, -2.8172245631, 0.9823954051

H, 0, -3.9691994501, -3.4840401988, 1.1048771707

C, 0, -1.8559023903, -3.2125850281, 1.4213304199

H, 0, -1.7266063952, -4.1866665144, 1.8862497765

C, 0, 3.4968751671, -1.3602867636, -2.3069652686

H, 0, 4.3079734282, -1.7066331698, -2.9415974884

C, 0, 1.2387554945, -0.535848731, -2.046423276
H, 0, 0.2937437175, -0.2441743722, -2.5045304903
C, 0, 2.2732663957, -0.9896036507, -2.8658063172
H, 0, 2.1286628048, -1.0476054389, -3.9420731351
C, 0, 2.6200747997, -0.8244093913, -0.1189142434
H, 0, 2.7676219155, -0.7688783186, 0.959467005
C, 0, 3.666401429, -1.2766264, -0.9252328412
H, 0, 4.6151446934, -1.5602007694, -0.4756033138
C, 0, -0.8534348487, 1.1438341809, -0.3823563507
C, 0, -0.9131051392, -1.1111521416, 0.662999578
C, 0, 1.3797420285, -0.4360871763, -0.6524352888
B, 0, 0.1753138227, 0.0747026938, 0.316316741
N, 0, 1.6926529123, 1.8429624127, 1.4071672115
O, 0, 0.6591438608, 0.4818563601, 1.6530387581
N, 0, 2.6062755488, 1.7605011398, 2.1173611624
O, 0, 3.2801618439, 1.2432339062, 2.9783781823

6a

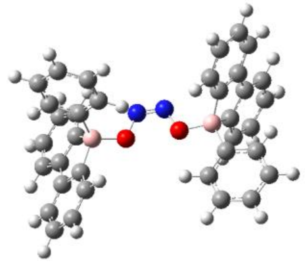

-2 1
C, 0, -4.4942568096, -1.4288854659, -0.5235574088
C, 0, -3.3657476205, -2.0692483942, 1.5031625524
C, 0, -4.4825593042, -0.3854650384, -1.5766160277
C, 0, -3.3484859664, 1.6351483011, -2.2207359052
C, 0, -4.091373492, 2.0267058796, 1.4137575948
C, 0, -1.850807046, 1.6243243, 2.1397940205
C, 0, -5.3442709817, -2.5299171444, -0.4258734501

H, 0, -2.604959392, -1.9003499238, 2.2651349503

C, 0, -4.207449616, -3.183746512, 1.6078021385

C, 0, -5.3259142307, -0.2939269661, -2.6836449026

H, 0, -2.591030087, 2.3985427575, -2.0493042805

C, 0, -4.1863038621, 1.7359298705, -3.3386643588

H, 0, -4.9183380873, 1.8337505636, 0.7294660109

C, 0, -4.2664951075, 2.9602207643, 2.4348591384

H, 0, -0.8957524636, 1.1119604733, 2.0292707305

C, 0, -2.0105270769, 2.5570257549, 3.1669880378

H, 0, -6.1148996863, -2.7099523023, -1.172716897

C, 0, -5.1948961885, -3.4104001845, 0.6477286837

H, 0, -4.0956642042, -3.8763969678, 2.4383215684

H, 0, -6.0976249577, -1.0402690611, -2.8620894127

C, 0, -5.1714506316, 0.7744283327, -3.5689057312

H, 0, -4.0723893874, 2.5662543129, -4.0314157738

C, 0, -3.2215697754, 3.2306713089, 3.319846341

H, 0, -5.2149849329, 3.4824449851, 2.5384895729

H, 0, -1.1859216137, 2.7620355653, 3.8459260578

H, 0, -5.8490841317, -4.273985753, 0.7347703251

H, 0, -5.8203821333, 0.8576266788, -4.4369592166

H, 0, -3.3486559124, 3.961830421, 4.1136722982

C, 0, 4.2911728885, 1.539553975, -0.1868308689

C, 0, 3.2220604545, 1.4711808531, 1.9686646505

C, 0, 4.2934915968, 0.8513237071, -1.5013746121

C, 0, 3.2377942525, -0.96587153, -2.6738469613

C, 0, 4.4221741362, -2.1057843327, 0.9410055323

C, 0, 2.1126050672, -2.6969660245, 1.1216131945

C, 0, 5.049312747, 2.6451447948, 0.1987123665

H, 0, 2.515943645, 1.0251958238, 2.6698708003

C, 0, 3.9686454597, 2.5882591483, 2.364137291

C, 0, 5.0475306501, 1.1716365472, -2.630531386

H, 0, 2.5371219957, -1.8005864174, -2.7066702489

C, 0, 3.9805656608, -0.6476993939, -3.8179196554

H, 0, 5.2028199278, -1.4077356958, 0.634857779

C, 0, 4.7887122781, -3.3019065429, 1.5545217047

H, 0, 1.0619614411, -2.467817189, 0.9559495884

C, 0, 2.4661855804, -3.9003500213, 1.7387638388

H, 0, 5.7585570787, 3.1051393734, -0.487021317

C, 0, 4.8826048505, 3.1685358271, 1.4830951345

H, 0, 3.8385116687, 3.0085779575, 3.358488864

H, 0, 5.7493810079, 2.0034493024, -2.6171677457

C, 0, 4.8849976102, 0.4153280093, -3.7932345841

H, 0, 3.8536683271, -1.2256356425, -4.7302425227

C, 0, 3.8071394103, -4.2092701647, 1.9575148628

H, 0, 5.8391906333, -3.5293811367, 1.7207274332

H, 0, 1.6911182238, -4.5978549811, 2.0486459656

H, 0, 5.4633588554, 4.0329522509, 1.794489148

H, 0, 5.4618321757, 0.6578076965, -4.6819539338

H, 0, 4.0861688177, -5.1442020859, 2.4364365437

N, 0, -0.545609004, 0.8914950616, -0.6358273988

N, 0, 0.690351182, 0.7848254515, -0.6224935175

O, 0, -1.2370322592, -0.1392738879, -0.0920713272

B, 0, -2.6956171963, 0.2034818868, 0.0717240711

C, 0, -3.4927563549, -1.1767335775, 0.4392786058

C, 0, -3.4743616145, 0.5725816052, -1.3236800204

C, 0, -2.8834468456, 1.3313168473, 1.2335762462

O, 0, 1.176003324, -0.3384843329, -0.0486546175

B, 0, 2.6777359291, -0.370820497, -0.026776572

C, 0, 3.3708099982, 0.9288230455, 0.6928284688

C, 0, 3.3800678316, -0.225382599, -1.5012476476

C, 0, 3.0779593432, -1.7670072815, 0.7047228452

6b

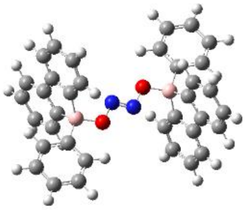

-2 1

C, 0, -2.1617711129, 0.6007404396, 3.6759410675

C, 0, -1.8899648868, -0.8556551592, 3.7666234663

C, 0, -0.1817212519, -2.480740306, 3.2807484219

H, 0, 0.8151149534, -2.7382734219, 2.921941379

C, 0, -1.1413880972, 2.6599814849, 2.9604122379

H, 0, -0.2958748261, 3.2128052561, 2.5500745135

C, 0, 1.300235286, 0.7153051655, 5.2394956759

H, 0, 0.2916237963, 0.7096207443, 5.6546903851

C, 0, -2.7524412707, -1.8607577945, 4.2047471165

H, 0, -3.7542851271, -1.6247746436, 4.5587559658

C, 0, -3.3357454826, 1.2742158755, 4.0140967791

H, 0, -4.1922479062, 0.7392831612, 4.4204692448

C, 0, -1.0427459697, -3.5002427076, 3.7053727364

H, 0, -0.7199776089, -4.5381071439, 3.6719016612

C, 0, 2.3799953168, 0.9210025735, 6.0959007455

H, 0, 2.2098657608, 1.0735972975, 7.1591775628

C, 0, -2.3187136079, 3.3460707089, 3.2827490105

H, 0, -2.3886782775, 4.4189757647, 3.1204054772

C, 0, 3.6808021303, 0.9335563195, 5.5888674224

H, 0, 4.5275260106, 1.0948983793, 6.2508042855

C, 0, 2.7858124814, 0.5319869784, 3.3766928989

H, 0, 2.956644505, 0.3824141789, 2.3131670543

C, 0, -2.3209347768, -3.1890984999, 4.1717306126

H, 0, -2.9859029479, -3.9828321118, 4.5024097053

C, 0, -3.4092409791, 2.6546103951, 3.812729672

H, 0, -4.3206978158, 3.1907902655, 4.0641232664

C, 0, 3.8782546554, 0.7385720758, 4.2236405183

H, 0, 4.8859515743, 0.7478323566, 3.8143885037

C, 0, 2.1617711129, -0.6007404396, -3.6759410675

C, 0, 1.8899648868, 0.8556551592, -3.7666234663

C, 0, 0.1817212519, 2.480740306, -3.2807484219

H, 0, -0.8151149534, 2.7382734219, -2.921941379

C, 0, 1.1413880972, -2.6599814849, -2.9604122379

H, 0, 0.2958748261, -3.2128052561, -2.5500745135

C, 0, -1.300235286, -0.7153051655, -5.2394956759

H, 0, -0.2916237963, -0.7096207443, -5.6546903851

C, 0, 2.7524412707, 1.8607577945, -4.2047471165

H, 0, 3.7542851271, 1.6247746436, -4.5587559658

C, 0, 3.3357454826, -1.2742158755, -4.0140967791

H, 0, 4.1922479062, -0.7392831612, -4.4204692448

C, 0, 1.0427459697, 3.5002427076, -3.7053727364

H, 0, 0.7199776089, 4.5381071439, -3.6719016612

C, 0, -2.3799953168, -0.9210025735, -6.0959007455

H, 0, -2.2098657608, -1.0735972975, -7.1591775628

C, 0, 2.3187136079, -3.3460707089, -3.2827490105

H, 0, 2.3886782775, -4.4189757647, -3.1204054772

C, 0, -3.6808021303, -0.9335563195, -5.5888674224

H, 0, -4.5275260106, -1.0948983793, -6.2508042855

C, 0, -2.7858124814, -0.5319869784, -3.3766928989

H, 0, -2.956644505, -0.3824141789, -2.3131670543

C, 0, 2.3209347768, 3.1890984999, -4.1717306126

H, 0, 2.9859029479, 3.9828321118, -4.5024097053

C, 0, 3.4092409791, -2.6546103951, -3.812729672

H, 0, 4.3206978158, -3.1907902655, -4.0641232664

C, 0, -3.8782546554, -0.7385720758, -4.2236405183

H, 0, -4.8859515743, -0.7478323566, -3.8143885037

O, 0, 0.7249875215, 0.2452851955, 1.4867156054

N, 0, -0.2376007298, -0.0319040256, 0.5681553078

C, 0, -0.5888537994, -1.1477244379, 3.3024293416

C, 0, 1.4671336109, 0.5133268304, 3.8576284756

C, 0, -1.0425616227, 1.2827947814, 3.1504437042

B, 0, 0.1899643417, 0.2343614949, 2.889800754

O, 0, -0.7249875215, -0.2452851955, -1.4867156054

N, 0, 0.2376007298, 0.0319040256, -0.5681553078

C, 0, 0.5888537994, 1.1477244379, -3.3024293416

C, 0, -1.4671336109, -0.5133268304, -3.8576284756

C, 0, 1.0425616227, -1.2827947814, -3.1504437042

B, 0, -0.1899643417, -0.2343614949, -2.889800754

Cartesian coordinates optimized at the M06-2X level in the THF solution:

2

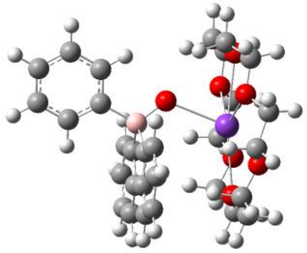

0 2

O, 0, 11.0854931328, 1.2432880479, 5.4788959509

O, 0, 10.4941898346, 1.2114435005, 2.748554309

C, 0, 14.7942921403, 2.4606174405, 3.3807614142

C, 0, 15.3435072792, 2.8986910785, 4.6053916668

C, 0, 15.8036425861, 1.9816651628, 5.5532827297

H, 0, 16.2488803443, 2.3226692631, 6.4857464298

C, 0, 14.6671652445, 1.0937704093, 3.1455966695

H, 0, 14.2346336917, 0.7390874639, 2.2115683938

C, 0, 15.6962311503, 0.613720335, 5.2899037351

H, 0, 16.0576001209, -0.1076377152, 6.0179546585

C, 0, 12.1869598267, 1.287694464, 6.3710856246

H, 0, 13.1247308694, 1.3437181062, 5.7989636515

H, 0, 12.2113703344, 0.3798314132, 6.9931736588

C, 0, 15.1224848836, 0.1699818435, 4.0959526213

H, 0, 15.0366416583, -0.8964305291, 3.9021745865

C, 0, 12.0574611337, 2.4917129461, 7.2653428021

H, 0, 11.1020997618, 2.4661942276, 7.8110236549

H, 0, 12.8786833547, 2.4798374625, 7.9981783035

C, 0, 10.0853391649, 0.1634799205, 3.6040639256

H, 0, 10.0522944628, -0.7897168701, 3.0557539281

H, 0, 9.0811615204, 0.3665242594, 4.0058513931

C, 0, 11.0947172116, 0.0491180492, 4.7159844428

H, 0, 10.8484669117, -0.8111453991, 5.3564095578
H, 0, 12.0938301375, -0.1099354734, 4.2847116614
C, 0, 9.7783923906, 1.2435926465, 1.5292357545
H, 0, 9.9568168366, 0.3191653304, 0.9602053974
C, 0, 10.2614634903, 2.4249143088, 0.729569746
H, 0, 11.3566793283, 2.3930226587, 0.6343047937
H, 0, 9.8090234762, 2.3942547917, -0.2732523193
H, 0, 8.6985813585, 1.3309392366, 1.7227924633
O, 0, 11.133710401, 6.062550665, 5.4175650282
O, 0, 10.5241538748, 6.029788931, 2.6883400688
C, 0, 14.8851404001, 4.9478622987, 3.4840418282
C, 0, 15.384139138, 4.3762926377, 4.676300873
C, 0, 15.8550071486, 5.1626999095, 5.7301674647
H, 0, 16.245264778, 4.7062916697, 6.6376513137
C, 0, 14.8674064496, 6.3411222972, 3.3857978256
H, 0, 14.5003865809, 6.8173468142, 2.4771869682
C, 0, 15.8294220239, 6.5538987579, 5.6035634454
H, 0, 16.1977931578, 7.1790385032, 6.4126483691
C, 0, 12.2559591994, 6.01282343, 6.2841765186
H, 0, 13.1753495805, 5.9007915885, 5.6918368449
H, 0, 12.3278534817, 6.9413336998, 6.8707253019
C, 0, 15.3342610798, 7.1419779959, 4.4366059543
H, 0, 15.3186053291, 8.2254366528, 4.3447464343
C, 0, 12.1058895922, 4.8469290469, 7.224353409
H, 0, 11.1607884567, 4.9208319536, 7.7833108167
H, 0, 12.9393223425, 4.8573582264, 7.9430724516
C, 0, 10.1343553183, 7.1034558179, 3.5206499619
H, 0, 10.1060457468, 8.0429258976, 2.9488446035
H, 0, 9.1321122941, 6.920597266, 3.9367976844
C, 0, 11.1559933951, 7.2335612451, 4.6196623312
H, 0, 10.9304780844, 8.1165244924, 5.2361320942

H, 0, 12.1530885435, 7.362587649, 4.1739475195

C, 0, 9.806987365, 5.9779521072, 1.4711847915

H, 0, 9.9950975903, 6.8871047387, 0.8811751739

C, 0, 10.2760344442, 4.7718314855, 0.7003733588

H, 0, 11.3716512385, 4.7894019833, 0.6020472713

H, 0, 9.8251269681, 4.7830838554, -0.3034967096

H, 0, 8.7262934049, 5.9051339802, 1.6660841481

K, 0, 11.2465046465, 3.6308555922, 3.8907378537

O, 0, 13.11652756, 3.6241316879, 2.0576577083

O, 0, 12.1321060896, 3.6544283236, 6.4661517841

O, 0, 9.8755788196, 3.609757444, 1.4028568606

C, 0, 15.4942314964, 3.7567499266, 1.0944269337

C, 0, 17.1959817981, 3.6188492895, -1.1742117587

H, 0, 17.8477955409, 3.5670010933, -2.0421852819

C, 0, 16.8601722833, 4.0760060267, 1.177287176

H, 0, 17.276480048, 4.3848039567, 2.1360534459

C, 0, 17.7036999367, 4.0060451669, 0.0677847603

H, 0, 18.7571862249, 4.2554426341, 0.1688776307

C, 0, 15.0114312364, 3.3721185849, -0.1655261215

H, 0, 13.9560593958, 3.1225600715, -0.2605002997

C, 0, 15.8416532093, 3.3031155063, -1.2875379858

H, 0, 15.4334962476, 3.0038430536, -2.2502611792

B, 0, 14.4977444224, 3.7779876665, 2.3919137237

BO-

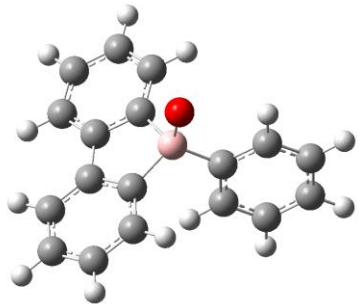

-1 2

C, 0, 0.5014441609, -0.6093098219, 1.1858849769

C, 0, -0.1004918252, -1.8013252506, 0.7283334348

C, 0, -0.5663572946, -2.7632271149, 1.627356893

H, 0, -1.0334523938, -3.6789091923, 1.2703611838

C, 0, 0.6359490478, -0.4053744761, 2.5571287129

H, 0, 1.097406646, 0.5076277569, 2.9308426329

C, 0, -0.4264820884, -2.5399500159, 2.9998236076

H, 0, -0.7850461573, -3.2827976919, 3.707418655

C, 0, 0.1713861167, -1.3662551276, 3.4647354158

H, 0, 0.2742489907, -1.2001658139, 4.5342344112

C, 0, 0.3962525988, -0.6556406082, -1.298380058

C, 0, -0.1549980523, -1.8362314328, -0.7515983419

C, 0, -0.6661718273, -2.853090616, -1.5604422205

H, 0, -1.0904935355, -3.7556584976, -1.1250718485

C, 0, 0.4155353121, -0.5265030084, -2.6876515565

H, 0, 0.8243732044, 0.3745002377, -3.1443121582

C, 0, -0.6284407066, -2.6999766934, -2.9489909808

H, 0, -1.0206645792, -3.4837817753, -3.5917752912

C, 0, -0.0896169098, -1.5405564272, -3.5119241048

H, 0, -0.0663311111, -1.427758161, -4.593252215

O, 0, 2.3715081999, 0.4088916975, 0.0216607163

C, 0, 0.206164252, 1.8025777837, -0.0685964687

C, 0, -1.0456998965, 4.3492770454, 0.0820750018

H, 0, -1.5249870193, 5.3231832402, 0.1376371085

C, 0, -1.151893701, 1.9781604753, -0.3875457403

H, 0, -1.7385939065, 1.1147758146, -0.7015225425

C, 0, -1.7752669318, 3.2246123021, -0.3112637528

H, 0, -2.8291395756, 3.3223636378, -0.5617577222

C, 0, 0.9129670822, 2.9498699266, 0.3238475691

H, 0, 1.9672086531, 2.84620378, 0.5736778396

C, 0, 0.3054305205, 4.206328552, 0.3995012865

H, 0, 0.8859407484, 5.0744419741, 0.7038340679

B, 0, 0.9397749784, 0.340642501, -0.1045685115

K^{+}

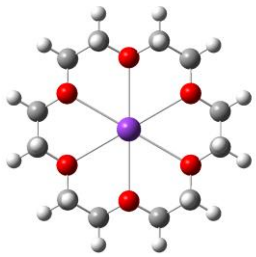

1 1

O, 0, 2.397657842, -1.3890653007, 0.1603173307

O, 0, 0.0000265811, -2.7789054819, -0.1685937868

C, 0, 3.5419709972, -0.6902006631, -0.3005261931

H, 0, 3.5298038516, -0.6276539008, -1.3986967971

H, 0, 4.4568979304, -1.2158971754, 0.0077889346

C, 0, 3.5420023439, 0.6902394607, 0.300461087

H, 0, 3.5300560244, 0.6276877556, 1.3986312904

H, 0, 4.4568057083, 1.2160353941, -0.008042829

C, 0, 1.1749061642, -3.4220584952, 0.2961424082

H, 0, 1.1777646148, -4.4766972118, -0.0132726144

H, 0, 1.2173819132, -3.3820012076, 1.394559028

C, 0, 2.3734404683, -2.7309852739, -0.2968132023

H, 0, 3.2847037271, -3.2570440888, 0.0215912538

H, 0, 2.3240070016, -2.7549492472, -1.3952433474

C, 0, -1.1749135869, -3.4219861775, 0.2961456467

H, 0, -1.1777928391, -4.4766534158, -0.0131589725

C, 0, -2.3733743531, -2.7309236995, -0.2969526173

H, 0, -2.3238133115, -2.7549017224, -1.3953743306

H, 0, -3.2847059511, -3.2569010756, 0.021371

H, 0, -1.2174531433, -3.3818043545, 1.394555691

O, 0, -0.0000265811, 2.7789054819, 0.1685937868

O, 0, -2.397657842, 1.3890653007, -0.1603173308

C, 0, 1.1749135869, 3.4219861775, -0.2961456467

H, 0, 1.2174531433, 3.3818043545, -1.394555691

H, 0, 1.1777928391, 4.4766534158, 0.0131589724

C, 0, 2.3733743531, 2.7309236995, 0.2969526172

H, 0, 2.3238133115, 2.7549017224, 1.3953743306

H, 0, 3.2847059511, 3.2569010756, -0.0213710001

C, 0, -2.3734404683, 2.7309852739, 0.2968132023

H, 0, -3.2847037271, 3.2570440888, -0.0215912539

H, 0, -2.3240070016, 2.7549492472, 1.3952433473

C, 0, -1.1749061642, 3.4220584952, -0.2961424082

H, 0, -1.1777646148, 4.4766972118, 0.0132726144

H, 0, -1.2173819132, 3.3820012076, -1.394559028

C, 0, -3.5419709972, 0.6902006631, 0.3005261931

H, 0, -4.4568979304, 1.2158971754, -0.0077889346

C, 0, -3.5420023439, -0.6902394607, -0.3004610871

H, 0, -3.5300560244, -0.6276877556, -1.3986312904

H, 0, -4.4568057083, -1.2160353941, 0.008042829

H, 0, -3.5298038516, 0.6276539008, 1.3986967971

K, 0, 0., 0., 0.

O, 0, 2.3975429931, 1.3889888518, -0.1601559315

O, 0, -2.3975429931, -1.3889888518, 0.1601559315

TS

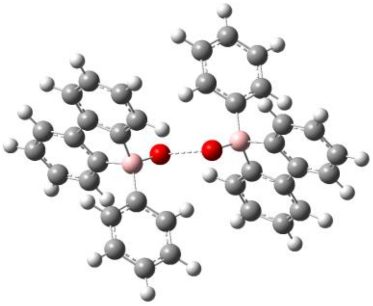

-2 1

O, 0, 1.0463404305, 0.0616573888, 1.029485565

O, 0, -0.8690427762, -0.0514512817, -0.8492638214

C, 0, 4.4072137226, -0.5692827384, 0.8062923969

C, 0, 2.7063538669, -0.6686374291, -0.8564522635

C, 0, 3.060721629, 2.5658753897, -1.1649839961

H, 0, 3.9871769191, 2.0172290322, -1.341597311

C, 0, 2.933198602, 3.8548942771, -1.6873061262

H, 0, 3.74758151, 4.2956493859, -2.2584527142

C, 0, 1.7563847799, 4.5783920706, -1.4778407759

H, 0, 1.6480588503, 5.5806862866, -1.8848483644

C, 0, 0.7200476901, 3.9952104609, -0.7470525156

H, 0, -0.2059246393, 4.5433686575, -0.5858106625

C, 0, 0.8640837783, 2.704129463, -0.2308026045

H, 0, 0.045284805, 2.2402791412, 0.3152091018

C, 0, 2.0357476894, 1.9559276989, -0.421248045

C, 0, -3.7807217625, 1.2031437717, 0.6395367155

C, 0, -2.5297498818, 0.6794320557, 1.0359023308

C, 0, -4.4431802881, 2.1786969765, 1.3883478366

H, 0, -5.4075093546, 2.5707156076, 1.0701738349

C, 0, 3.9574640096, -1.1924316988, -0.4606216433

C, 0, 2.132750362, -1.161528104, -2.0306622326

H, 0, 1.1593912482, -0.7873080903, -2.3444364597

C, 0, 3.7205903933, 1.0483072826, 2.4593727822

H, 0, 3.0038354967, 1.7762625883, 2.8367620994

C, 0, 3.4581625346, 0.3671465291, 1.2719951141

C, 0, 2.7831534648, -2.1450978778, -2.7874290103

H, 0, 2.3230381141, -2.5270281943, -3.6960864959

C, 0, -3.847688765, 2.6553855019, 2.5587352851

H, 0, -4.3481169016, 3.4177714908, 3.1506675402

C, 0, 5.5916586364, -0.8094967418, 1.5064202572

H, 0, 6.3207126145, -1.5291188494, 1.1387241707

C, 0, -5.4141647024, 0.8200262834, -1.3280473448

H, 0, -6.1433448917, 1.5396782527, -0.9606604387

C, 0, -2.6072602041, 2.1562737974, 2.9665734002

H, 0, -2.1474697315, 2.5383888496, 3.8753163084

C, 0, 4.6196386543, -2.1678521728, -1.2098597328

H, 0, 5.5840852176, -2.5599348661, -0.8921144356

C, 0, 4.0237219741, -2.6443001121, -2.3801325364

H, 0, 4.5239339644, -3.4065647424, -2.9724042774

C, 0, -1.956589594, 1.1725480894, 2.2102371036

H, 0, -0.9833497378, 0.7983814026, 2.5244583824

C, 0, -4.2299966444, 0.5798293122, -0.6274543986

C, 0, -1.8591780904, -1.9452671737, 0.6014748453

C, 0, -2.8844009998, -2.5550029624, 1.3450402985

H, 0, -3.810871509, -2.0062683529, 1.5213081565

C, 0, -2.7571031858, -3.8439383312, 1.8676339871

H, 0, -3.5716758475, -4.2845387239, 2.4386307034

C, 0, -1.5802812354, -4.5675517735, 1.6586260288

H, 0, -1.472137001, -5.5697808308, 2.0658415963

C, 0, -0.5437046843, -3.9845740163, 0.928021601

H, 0, 0.3822746173, -4.5328223451, 0.7671208987

C, 0, -0.6875106389, -2.6935750587, 0.4115013547

H, 0, 0.1314784437, -2.22989519, -0.1343728968

C, 0, -4.7261271469, -0.8009892224, -2.9917931106

H, 0, -4.9235385515, -1.3381875038, -3.9162532635

C, 0, 5.8354644723, -0.1149562576, 2.6945177187

H, 0, 6.7532928513, -0.2954829132, 3.2482287533

C, 0, 4.9042894847, 0.8115083024, 3.1704639746

H, 0, 5.1020800607, 1.3487170232, 4.0948366294

C, 0, -3.2807844973, -0.3566502324, -1.0927151312

C, 0, -5.6575055919, 0.1254648076, -2.5162227435

H, 0, -6.5751126889, 0.3059878008, -3.0703017615

C, 0, -3.5427192985, -1.0377949697, -2.2802201396

H, 0, -2.8258122432, -1.7657488256, -2.6573243005

B, 0, 2.1754007039, 0.4581494848, 0.2215082568

B, 0, -1.9984636075, -0.4476056365, -0.0416529098

5

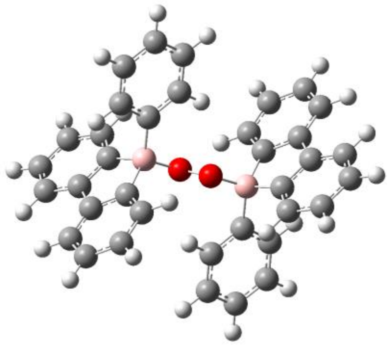

-2 1

O, 0, 0.511684241, 0.0508565397, 0.5164820358

O, 0, -0.4985474718, -0.0674721046, -0.5142760252

C, 0, 3.9712183697, -0.3566699469, 0.5786663737

C, 0, 2.4094972208, -0.467456629, -1.2192814712

C, 0, 2.4864326432, 2.7224566677, -1.5157041156

H, 0, 3.3620904641, 2.1643317432, -1.8497556838

C, 0, 2.3377063401, 4.0487323774, -1.9296169784

H, 0, 3.0867746302, 4.5067301082, -2.5723040068

C, 0, 1.2246453972, 4.7863264228, -1.5212895937

H, 0, 1.0992565802, 5.81735297, -1.8425181257

C, 0, 0.2723226777, 4.1793265443, -0.6990599217

H, 0, -0.6041699345, 4.7398569189, -0.3804611877

C, 0, 0.4373871088, 2.8531812167, -0.2927365377

H, 0, -0.3153543504, 2.3776823532, 0.3344036368

C, 0, 1.5477146906, 2.0862238945, -0.6864293627

C, 0, -3.6396872257, 0.9296275817, 0.7482806532

C, 0, -2.3960792788, 0.4510229363, 1.2216848276

C, 0, -4.4103926822, 1.8375347987, 1.4784623385

H, 0, -5.3653574526, 2.1953374372, 1.0975270838

C, 0, 3.6530022601, -0.9460993022, -0.7456429305

C, 0, 1.9683935255, -0.931345518, -2.4612580991

H, 0, 1.0161209624, -0.5811804799, -2.8554098185

C, 0, 3.0723989965, 1.1351279601, 2.2401013555

H, 0, 2.2914401006, 1.8067265556, 2.5968107881

C, 0, 2.9310782296, 0.498823177, 1.0063275884

C, 0, 2.7284405613, -1.8460393126, -3.2025440932

H, 0, 2.3664376291, -2.2032803406, -4.163955221

C, 0, -3.9393875848, 2.2899818673, 2.7137919518

H, 0, -4.5260764597, 2.9993677289, 3.2922015201

C, 0, 5.1131709193, -0.5675912057, 1.3540823794

H, 0, 5.9091366007, -1.2263583677, 1.0115578693

C, 0, -5.1003874309, 0.550864137, -1.3510150082

H, 0, -5.8962951076, 1.2096166999, -1.0083286631

C, 0, -2.7144515333, 1.8300208401, 3.2047544047

H, 0, -2.3521712862, 2.1874628841, 4.1659869855

C, 0, 4.423917933, -1.8538523579, -1.475794599

H, 0, 5.3788043589, -2.2116856196, -1.0946907002

C, 0, 3.9532507123, -2.3060714545, -2.7113386566

H, 0, 4.5401142562, -3.015323907, -3.2897347902

C, 0, -1.9546209429, 0.9151635991, 2.4634464165

H, 0, -1.0022234375, 0.5650970047, 2.8573843175

C, 0, -3.9582093814, 0.3400763411, -0.5758974679

C, 0, -1.5345679714, -2.1027539501, 0.6887781775

C, 0, -2.4733922189, -2.7390031699, 1.5179227661

H, 0, -3.3491159111, -2.1808963734, 1.8518326313

C, 0, -2.3246875218, -4.0652666015, 1.9318786149

H, 0, -3.073837105, -4.523278699, 2.5744607635

C, 0, -1.2115431867, -4.8028339935, 1.5237239794

H, 0, -1.0861723011, -5.833852899, 1.8449842865

C, 0, -0.2591204277, -4.1958199175, 0.7016233142

H, 0, 0.6174320191, -4.7563312407, 0.3831563348

C, 0, -0.4241605229, -2.8696830198, 0.2952573654

H, 0, 0.3286563333, -2.3941760061, -0.3317863292

C, 0, -4.196654116, -0.9416196584, -3.0298369952

H, 0, -4.2915926366, -1.4396183818, -3.9921275868

C, 0, 5.2274461175, 0.0779614731, 2.5889278158

H, 0, 6.1100065677, -0.0803111922, 3.20374781

C, 0, 4.2090343849, 0.924766151, 3.0328060232

H, 0, 4.3037250645, 1.4226564504, 3.9951772544

C, 0, -2.9181357215, -0.5153951978, -1.0037667335

C, 0, -5.2149791524, -0.0948129398, -2.5857640376

H, 0, -6.0977186107, 0.0633603544, -3.2003527982

C, 0, - 3.0597899917, -1.1518516909, -2.2374269143

H, 0, -2.2789153511, -1.8234669688, -2.5942870829

B ,0, 1.7390929013, 0.5629737295, -0.1233222085

B, 0, -1.7259274904, -0.5795040175, 0.1256560806

Reference

1. Fulmer, G. R.; Miller, A. J. M.; Sherden, N. H.; Gottlieb, H. E.; Nudelman, A.; Stoltz, B. M.; Bercaw, J. E.; Goldberg, K. I., NMR Chemical Shifts of Trace Impurities: Common Laboratory Solvents, Organics, and Gases in Deuterated Solvents Relevant to the Organometallic Chemist. *Organometallics* **2010**, *29* (9), 2176-2179.

2. Li, S.; Shiri, F.; Xu, G.; Yiu, S.-M.; Lee, H. K.; Ng, T. H.; Lin, Z.; Lu, Z., Reactivity of a Hexaaryldiboron(6) Dianion as Boryl Radical Anions. *J. Am. Chem. Soc.* **2024**, *146* (25), 17348-17354.

3. Elangovan, S.; Randt, T.; Irran, E.; Klare, H. F. T.; Oestreich, M., Synthesis of a Cationic Cobalt-Selenolate Complex for Cooperative Sn-H Bond Activation: Solvent-Dependent Stereoselectivity in Alkyne Hydrostannylation. *Organometallics* **2024**, *43* (14), 1619-1624.

4. Bruker **2012**. SAINT. Bruker AXS Inc., Madison, Wisconsin, USA.

5. G. Sheldrick, SADABS—Bruker AXS Area Detector Scaling and Absorption **2008**.

6. Sheldrick, G. M., SHELXT - integrated space-group and crystal-structure determination. *Acta Crystallogr A Found Adv.* **2015**, *71* (Pt 1), 3-8.

7. Sheldrick, G. M., Crystal structure refinement with SHELXL. *Acta Cryst.* C. **2015**, *71* (Pt 1), 3-8.

8. Dolomanov, O. V.; Bourhis, L. J.; Gildea, R. J.; Howard, J. A. K.; Puschmann, H., OLEX2: a complete structure solution, refinement and analysis program. *J. Appl. Cryst.* **2009**, *42* (2), 339-341.

9. Spek, A., Single-crystal structure validation with the program PLATON. *J. Appl. Cryst.* **2003**, *36* (1), 7-13.

10. Zhao, Y.; Truhlar, D. G., Density Functional for Spectroscopy: No Long-Range Self-Interaction Error, Good Performance for Rydberg and Charge-Transfer States, and Better Performance on Average than B3LYP for Ground States. *Phys. Chem. A.* **2006**, *110* (49), 13126-13130.

11. McLean, A. D.; Chandler, G. S., Contracted Gaussian basis sets for molecular calculations. I. Second row atoms, Z=11-18. *Chem. Phys.* **1980**, *72* (10), 5639-5648.

12. Krishnan, R.; Binkley, J. S.; Seeger, R.; Pople, J. A., Self-consistent molecular orbital methods. XX. A basis set for correlated wave functions. *Chem. Phys.* **1980**, *72* (1), 650-654.

13. Scalmani, G.; Frisch, M. J., Continuous surface charge polarizable continuum models of solvation. I. General formalism. *Chem. Phys.* **2010**, *132* (11).

14. Kaim, W.; Hosmane, N. S.; Záliš, S.; Maguire, J. A.; Lipscomb, W. N., Boron Atoms as

Spin Carriers in Two- and Three-Dimensional Systems. *Angew. Chem. Int. Ed.* **2009**, *48* (28), 5082-5091.

15. Hratchian, H. P.; Schlegel, H. B., Accurate reaction paths using a Hessian based predictor-corrector integrator. *Chem. Phys.* **2004**, *120* (21), 9918-9924.

16. Hratchian, H. P.; Schlegel, H. B., Using Hessian Updating To Increase the Efficiency of a Hessian Based Predictor-Corrector Reaction Path Following Method. *J. Chem. Theory Comput.* **2005**, *1* (1), 61-69.

17. Frisch, M. J.; Trucks, G. W.; Schlegel, H. B.; Scuseria, G. E.; Robb, M. A.; Cheeseman, J. R.; Scalmani, G.; Barone, V.; Petersson, G. A.; Nakatsuji, H.; Li, X.; Caricato, M.; Marenich, A. V.; Bloino, J.; Janesko, B. G.; Gomperts, R.; Mennucci, B.; Hratchian, H. P.; Ortiz, J. V.; Izmaylov, A. F.; Sonnenberg, J. L.; Williams; Ding, F.; Lipparini, F.; Egidi, F.; Goings, J.; Peng, B.; Petrone, A.; Henderson, T.; Ranasinghe, D.; Zakrzewski, V. G.; Gao, J.; Rega, N.; Zheng, G.; Liang, W.; Hada, M.; Ehara, M.; Toyota, K.; Fukuda, R.; Hasegawa, J.; Ishida, M.; Nakajima, T.; Honda, Y.; Kitao, O.; Nakai, H.; Vreven, T.; Throssell, K.; Montgomery Jr., J. A.; Peralta, J. E.; Ogliaro, F., M. J.; Heyd, J. J.; Brothers, E. N.; Kudin, K. N.; Staroverov, V. N.; Keith, T. A.; Kobayashi, R.; Normand, J.; Raghavachari, K.; Rendell, A. P.; Burant, J. C.; Iyengar, S. S.; Tomasi, J.; Cossi, M.; Millam, J. M.; Klene, M.; Adamo, C.; Cammi, R.; Ochterski, J. W.; Martin, R. L.; Morokuma, K.; Farkas, O.; Foresman, J. B.; Fox, D. J. Gaussian 16 Rev. A.03, Wallingford, CT, **2016**.
